# Rules for the design of aza-glycine stabilized triple-helical collagen peptides†

**DOI:** 10.1039/d0sc03003a

**Published:** 2020-07-21

**Authors:** Samuel D. Melton, Emily A. E. Brackhahn, Samuel J. Orlin, Pengfei Jin, David M. Chenoweth

**Affiliations:** Department of Chemistry, University of Pennsylvania 231 South 34th Street Philadelphia Pennsylvania 19104 USA dcheno@sas.upenn.edu

## Abstract

The stability of the triple-helical structure of collagen is modulated by a delicate balance of effects including polypeptide backbone geometry, a buried hydrogen bond network, dispersive interfacial interactions, and subtle stereoelectronic effects. Although the different amino acid propensities for the Xaa and Yaa positions of collagen's repeating (Glycine–Xaa–Yaa) primary structure have been described, our understanding of the impact of incorporating aza-glycine (azGly) residues adjacent to varied Xaa and Yaa position residues has been limited to specific sequences. Here, we detail the impact of variation in the Xaa position adjacent to an azGly residue and compare these results to our study on the impact of the Yaa position. For the first time, we present a set of design rules for azGly-stabilized triple-helical collagen peptides, accounting for all canonical amino acids in the Xaa and Yaa positions adjacent to an azGly residue, and extend these rules using multiple azGly residues. To gain atomic level insight into these new rules we present two high-resolution crystal structures of collagen triple helices, with the first peptoid-containing collagen peptide structure. In conjunction with biophysical and computational data, we highlight the critical importance of preserving the triple helix geometry and protecting the hydrogen bonding network proximal to the azGly residue from solvent. Our results provide a set of design guidelines for azGly-stabilized triple-helical collagen peptides and fundamental insight into collagen structure and stability.

## Abstract 2

Guidelines for incorporating aza-glycine residues in collagen peptides are presented, detailing their effects on triple-helical thermal stability.

## Graphical abstract

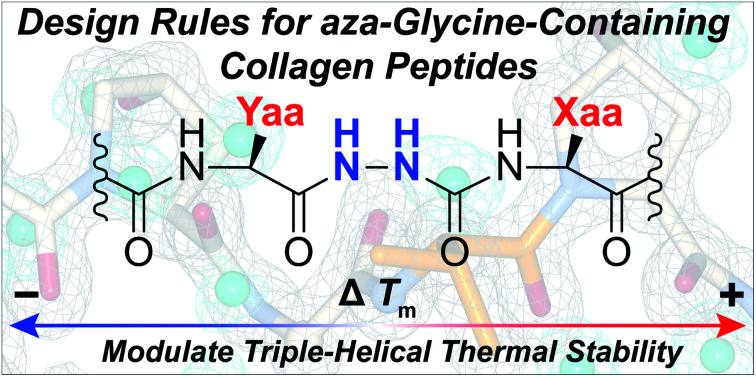

## Introduction

Collagen, the most abundant protein in the human body, serves as a critical structural element in skin, blood vessels, muscles, bones, and cartilage.^1–3^ Collagen proteins play key roles in several fields acting as pharmaceutical agents, matrices for tissue engineering, scaffolds for tissue regrowth, wound healing, and the development of biomaterials.^2,4–6^ Collagen's structure involves the assembly of three polyproline II helices, with each individual chain consisting of a three residue repeat sequence with the general form (Glycine–Xaa–Yaa). Within collagen's trademark triple-helical structure, three polyproline II helices are stitched together by a network of interstrand hydrogen bonding along the central axis of the right-handed triple helix. The primary sequence consists of two variable positions, Xaa and Yaa, and a conserved glycine (Gly, G) residue. The Xaa and Yaa positions are most often occupied by proline (Pro, P) and hydroxyproline (Hyp, O), respectively, resulting in the most stabilizing and triple helix promoting triplet sequence (Gly–Pro–Hyp).^7^ The conserved Gly residues are an essential part of collagen's primary sequence, as the small size and conformational flexibility of the residues enable the tight packing of the triple helix and optimal interstrand hydrogen bond formation.^8^ The propensity of a residue to be found in either the Xaa or Yaa position is influenced by the inherently different steric properties of the two positions in the collagen triple helix. The side chains of residues found in the Xaa position are more exposed to solvent than those in the Yaa position and more removed from side chains of neighboring strands.^9–11^ Variability in the Xaa and Yaa positions is essential for controlling collagen–protein interactions as binding is determined, in part, by the presence of recognition sequences within the collagen chains.^12,13^ Beyond this, the triple-helical thermal stability of collagen mimetic peptides (CMPs) containing these protein recognition sites is essential as triple-helical topology is often required for binding.^14,15^ The triple helix is stabilized by the backbone NH groups of the Gly residues acting as hydrogen bond donors to the carbonyl oxygen of Xaa position residues in neighboring chains. In addition to this, evidence exists for hydrogen bonds between the CH-α of Gly or Yaa residues with Gly and Xaa position residues in neighboring chains.^16,17^ Furthermore, an extensive network of hydrogen bonds mediate interactions between the three chains along the surface of the helix bundle.^18^ For example, the NH groups of Xaa position residues form hydrogen bonds with carbonyl groups of Gly residues through a single water molecule.^19,20^

**Fig. 1:**
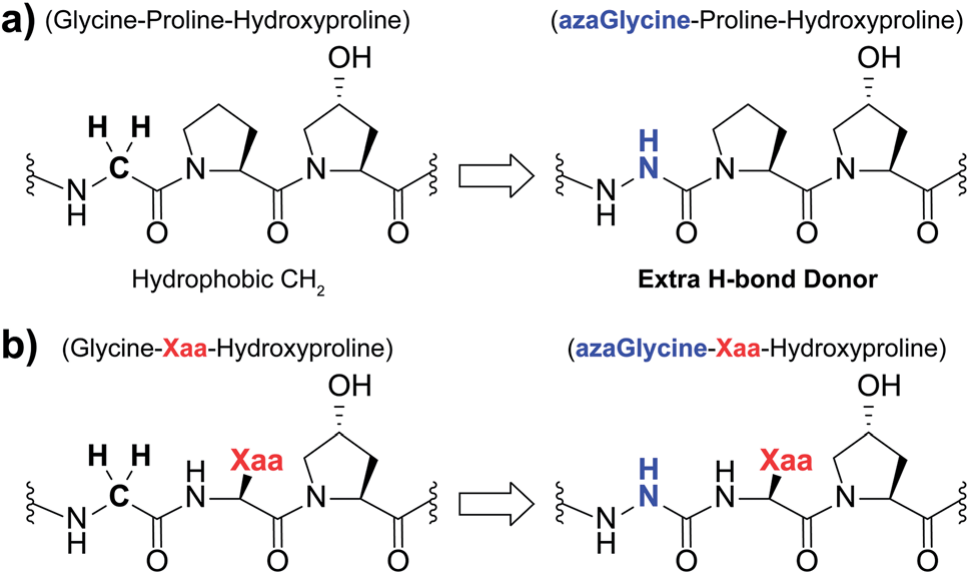
(a) Structural representation of an azGly substitution within a (Gly–Hyp–Pro) triplet. (b) Structural representation of an azGly substitution adjacent to a variable Xaa position residue.

Aza-amino acids, characterized by substitution of the residue's Cα for N, have seen extensive use in the literature for applications including structural studies and therapeutics.^21–28^ Aza-amino acids and aza-peptides exhibit several advantageous qualities including resistance to enzymatic degradation.^28,29^ Our group has reported the use of aza-glycine (azGly, azG) to serve as a glycine mimic within the collagen triple helix (Fig. 1a).^30–33^ In most cases, incorporation of this aza-amino acid within a simple model system results in increased triple-helical thermal stability as assessed by thermal melting studies. The azGly residues stabilize the collagen triple helix in two primary methods: the formation of additional interstrand hydrogen bonds *via* the azGly residue's NH-α and the restricted conformational flexibility compared to Gly residues. Incorporation of an azGly residue adjacent to a central, variable Yaa position in CMPs provides a reliable replacement for glycine residues.^33^ The identity of the Yaa position can significantly affect azGly's impact on triple-helical thermal stability, ranging from high increases in the melting point of the collagen triple helix (*T*_m_) to essentially no difference when compared to the control sequence. A sequence independent strategy for stabilizing CMPs using azGly residues positioned in triplet repeats positioned away from central substitution site provides a general tool for stabilizing triple-helical collagen peptides. Until now, our understanding of azGly's affect in collagen peptides has been limited to these specific cases. In this current work, we develop a deeper understanding by detailing the impact of neighboring Xaa residues on the ability of an azGly residue to act as a stabilizing agent (Fig. 1b). Along with our previous studies, this work culminates in the development of a new set of guidelines for the incorporation of azGly residues in collagen peptides for any given peptide sequence. This work facilitates, advances, and informs the design of novel, ultra-stable azGly-containing CMPs for applications in a diversity of fields including biomaterials, therapeutics, peptidomimetics, and beyond.

## Results and discussion

### Single-site incorporation of aza-glycine

**Fig. 2:**
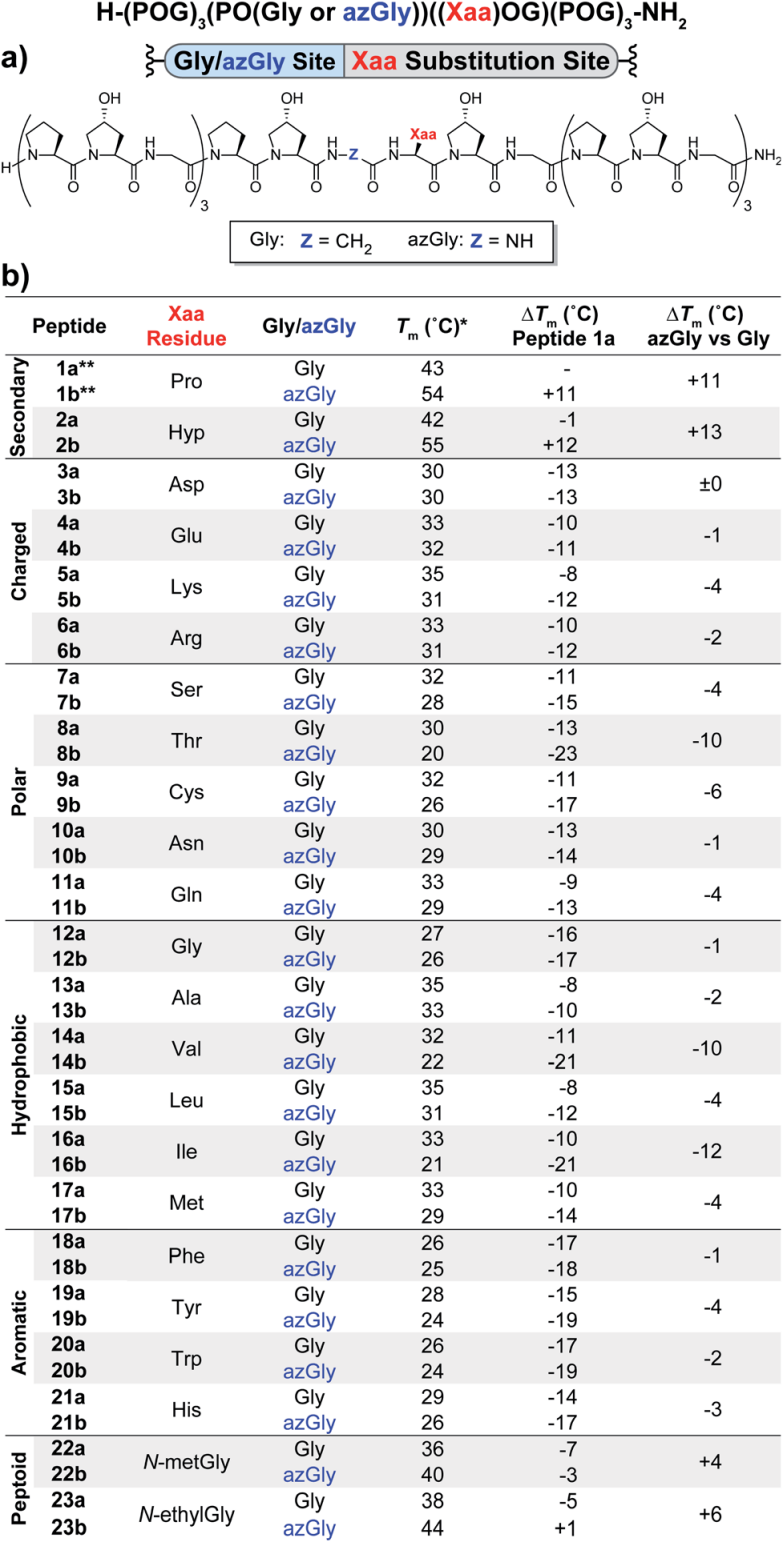
(Top) Sequence representation of CMPs **1a–23a** and **1b–23b**. (a) Graphical depiction showing azGly substitution sites surrounding a central Xaa position substitution. (b) *T*_m_ values of each CMP compared to **1a**, the POG control sequence, and the degree of azGly thermal stabilization within each pair of CMPs with the same Xaa substitution, *e.g.***10a** and **10b**. CMPs are grouped based on physical properties, *e.g.* charged residues. *For all peptides, the standard deviation between three triple-helical thermal stability measurements for each entry was ≤1 °C. **Data for CMPs **1a** and **1b** are taken from ref. 33.

The first set of CMPs were synthesized with the general form [H–(POG)_4_(Xaa–Hyp–Gly)(POG)_3_–NH_2_]. This series was used to evaluate the effect of a central Xaa position substitution compared to CMP **1a**, [H–(POG)_8_–NH_2_], the most stable natural CMP in this library, and to serve as baseline controls for the corresponding azGly-substituted CMPs. This second set of CMPs, with the general form [H–(POG)_3_(POazG)(Xaa–Hyp–Gly)(POG)_3_–NH_2_], was used to evaluate the consequences of incorporating an azGly residue adjacent to the variable Xaa position. Fig. 2 shows the triple-helical thermal stability of each CMP, *i.e.* the *T*_m_ value, which was determined using circular dichroism (CD) and monitoring the characteristic signal of the collagen triple helix at 225 nm (see ESI for Experimental details†). Each CMP is individually compared to **1a**, and within each pair of CMPs the triple-helical thermal stability of the azGly-containing CMP is compared to the Gly-containing control (*e.g.* CMP **10b** is compared to both **1a** and **10a**). The results can be broadly divided into groups based on the properties of the Xaa position residue: secondary, charged, polar, hydrophobic, or aromatic.

Previously, we established that incorporating an azGly residue adjacent to an Xaa position Pro residue resulted in an increase of its triple-helical *T*_m_ value by approximately 11 °C for a central substitution.^30,31^ Indeed, both of the secondary amino acids typically found in collagen, proline and hydroxyproline, exhibited increased triple-helical thermal stability when an azGly residue was adjacently incorporated. The Xaa position Hyp CMP (**2b**) displayed a substantial increase of 13 °C in its triple-helical *T*_m_ value, slightly exceeding the stability gained when Pro is present in the Xaa position (Fig. 2). The azGly afforded increase in triple-helical thermal stability is maximized when a secondary amino acid is present in the adjacent Xaa position.

The azGly-containing CMPs that contained charged residues in the Xaa position (**3b** (aspartate, Asp, D), **4b** (glutamate, Glu, E), **5b** (lysine, Lys, K), and **6b** (arginine, Arg, R)) universally exhibited decreases in their triple-helical thermal stability compared to their non-azGly-containing controls. The azGly-containing CMPs with the acidic side chains Asp and Glu exhibited marginal destabilization, with reduced *T*_m_ values of 0 to −1 °C compared to their controls. The azGly-containing CMPs with the basic side chains Lys (**5b**) and Arg (**6b**) exhibited moderate destabilization, with reduced *T*_m_ values of −4 to −2 °C, respectively, compared to their controls.

The azGly-containing CMPs that contained non-charged polar side chains in the Xaa position (**7b** (serine, Ser, S), **9b** (cystine, Cys, C), **10b** (asparagine, Asn, N), and **11b** (glutamine, Gln, Q)) displayed varied degrees of reduced triple-helical *T*_m_ values compared to their controls, ranging from −1 °C in the case of Asn, −4 °C with Gln or Ser, to −6 °C with Cys. However, the threonine (Thr, T) containing CMP **8b** exhibited a substantial decrease in its triple-helical *T*_m_ value of −10 °C, almost double the average decrease observed with the other polar residues.

The azGly-containing CMPs with hydrophobic residues followed a trend similar to that which was observed with charged residues, albeit with two notable exceptions. The azGly-containing CMPs that contained non-β-branched residues (**12b** (glycine, Gly, G), **13b** (alanine, Ala, A), **15b** (leucine, Leu, L), and **17b** (methionine, Met, M)) all exhibited small to moderate decreases in triple-helical thermal stability compared to their control counterparts. These *T*_m_ reductions ranged from −1 °C in the case of Gly to −4 °C in the case of Met. Drawing a parallel to our results with Yaa position substitutions, the CMPs containing β-branched side chains (**14b** (valine, Val, V) and **16b** (isoleucine, Ile, I)) exhibited the highest magnitude of decreased thermal stability with *T*_m_ value reductions ranging from −12 to −10 °C. This behavior is analogous to the result observed with the Thr-containing CMP **8b**, reinforcing the conclusion that azGly residues positioned adjacent to β-branched residues are detrimental to triple-helical thermal stability. The attenuated thermal stability of β-branched residues compared to other residues indicates steric bulk around Cβ plays an important role in weakening the triple-helical assembly.

The azGly-containing CMPs that containined aromatic residues (**18b** (phenylalanine, Phe, F), **19b** (tyrosine, Tyr, Y), **20b** (tryptophan, Trp, W), and **21b** (histidine, His, H)) exhibited decreased triple-helical thermal stability compared to their non-azGly-containing controls. The reductions in *T*_m_ values ranged from −1 °C in the case of Phe to −4 °C in the case of Tyr. Overall, the trend of reduced thermal stability with CMPs containing aromatic side chains was consistent with most other residues, with all CMPs exhibiting small to moderate decreases in triple-helical thermal stability.

### Incorporation of multiple aza-glycine residues

**Fig. 3:**
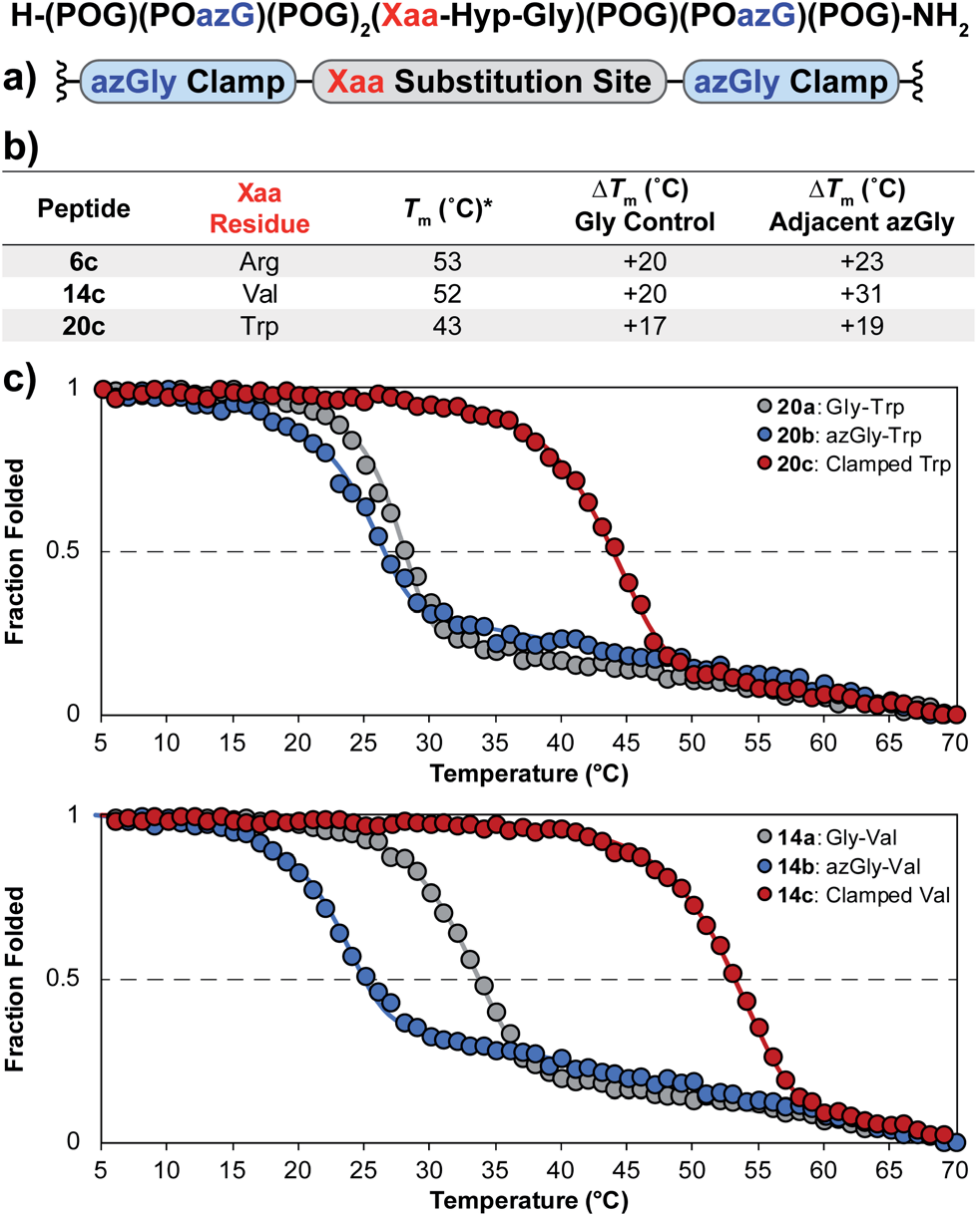
(top) Sequence representation of CMPs **6c**, **14c**, and **20c**. (a) Graphical depiction showing azGly substitution sites surrounding a central Xaa position substitution. (b) *T*_m_ values of clamped CMPs compared to **1a**, the POG control sequence, the corresponding Gly controls (CMPs **6a**, **14a**, and **20a**), and the adjacent azGly sequence (CMPs **6b**, **14b**, and **20b**). The sequences and data for these comparison CMPs are available in Chart 1 and Fig. S2.† (c) CD thermal melting data for Trp-containing CMPs **20a**, **20b**, and **20c** and for Val-containing CMPs **14a**, **14b**, and **14c**. *For all peptides, the standard deviation between three triple-helical thermal stability measurements for each entry was ≤1 °C.

Using the general sequence independent method for increasing the triple-helical thermal stability of CMPs, we synthesized several peptides with different central Xaa substitutions.^33^ The CMPs were synthesized with the general form [H–(POG)(POazG)(POG)_2_(Xaa–Hyp–Gly)(POG)(POazG)(POG)–NH_2_], with the two azGly residues acting as “clamps” on the ends of the triple helix surrounding the substitution site. Keeping in line with our previous Yaa position study, and to provide a range of side chain diversity, we selected the Xaa position substitutions Arg, Val, and Trp. Valine is a particularly relevant choice due to the extremely large degree of instability observed with the azGly-containing CMP **14b** compared to its natural control. The Trp-containing CMP, on the other hand, exhibited a minor decrease in thermal stability when the azGly substitution was made. However, Trp was one of the most significantly destabilizing residues in the Xaa position when compared to the [H–(POG)_8_–NH_2_] control. For each “clamped” peptide, we compared it to the non-azGly-containing control peptide and the single-site azGly-containing peptide of the corresponding residue (*e.g.* CMP **6c** is compared to **6a** and **6b**). In all three cases, the triple-helical thermal stability of the “clamped” CMPs significantly surpassed their corresponding control and, unsurprisingly, their corresponding single-site azGly-containing CMP (Fig. 3). Both the Arg and Val “clamped” CMPs exhibited triple-helical *T*_m_ values increases of 20 °C while the Trp “clamped” CMP exhibited an increase of 17 °C. These results readily agree with our previous work with similarly “clamped” CMPs bearing central Yaa position substitutions with the same three residues (Arg, Val, and Trp), further supporting this strategy as a highly generalizable and widely applicable method.

### Peptoid incorporation

In the context of amino acid variation at the Yaa position, the effect of azGly on the triple-helical *T*_m_ ranges from +0 to +11 °C, depending on the residue. When comparing these results to those from amino acid variation at the Xaa position, the following question arises: why does the incorporation of an azGly residue next to most amino acids in the Xaa position result in decreased triple-helical thermal stability? Interestingly, the backbone NH of the Xaa positions in the collagen triple helix are oriented toward solvent and can better participate in solvent interactions. Comparatively, the backbone NH of the Yaa positions are oriented more toward the center of the triple helix and thus are more shielded from solvent.^34,35^ Increasing the solvent accessibility of the peptide backbone can weaken and disrupt the interstrand hydrogen bonds essential for maintaining collagen's triple-helical structure. We hypothesize that the degree of peptide backbone solvent exposure, in the local region of the Xaa position, is critical when an adjacent azGly residue is present. Exposure of the azGly residue's NH-α, capable of forming an additional interstrand hydrogen bond, to solvent will negatively affect its ability to form productive interstrand H-bonds. Disruption of this local interstrand hydrogen bond network would potentially explain the observed decrease in triple-helical thermal stability upon incorporation of an azGly residue adjacent to most Xaa position amino acids and why the presence of a secondary amino acid (*i.e.* Pro and Hyp) resulted in increased stability.

To further investigate this, we took inspiration from work done by Goodman *et. al.* with incorporation of the peptoid residue *N*-isobutylglycine (Nleu) in collagen peptides.^36–39^ They incorporated Nleu in the Xaa and Yaa positions of collagen-like peptides, demonstrating (Gly–Nleu–Pro) sequences exhibited increased thermal stability and faster refolding times compared to (Gly–Pro–Nleu) sequences. Using computational models, the authors explained the increased triple-helical thermal stability resulted from the Xaa position Nleu residues being better positioned to form interchain hydrophobic interactions.^36,37^ Additionally, the *N*-substitution of peptoid residues removed the ability of the backbone N to act as a hydrogen bond donor. In our Xaa model system, we incorporated the two peptoid residues *N*-methylglycine (NmetG) in CMPs **22a** and **22b**, and *N*-ethylglycine (NethylG) in CMPs **23a** and **23b**. The peptoid-substituted non-azGly-containing control CMPs (**22a** and **23a**) surpassed most of the canonical amino acids in terms of triple-helical thermal stability, with the only exceptions being the CMPs containing Xaa substitutions of either Pro or Hyp. Excitingly, in both cases the azGly-containing CMPs **22b** and **23b** exhibited enhanced triple-helical thermal stability compared to their controls with increases in their *T*_m_ values of +4 °C for NmetG and +6 °C for NethylG. We suspect the slightly increased triple-helical thermal stability observed with NethylG is a result of the increased steric bulk and hydrophobicity compared to NmetG, further increasing favorable interchain hydrophobic interactions.

### Crystallography

**Fig. 4:**
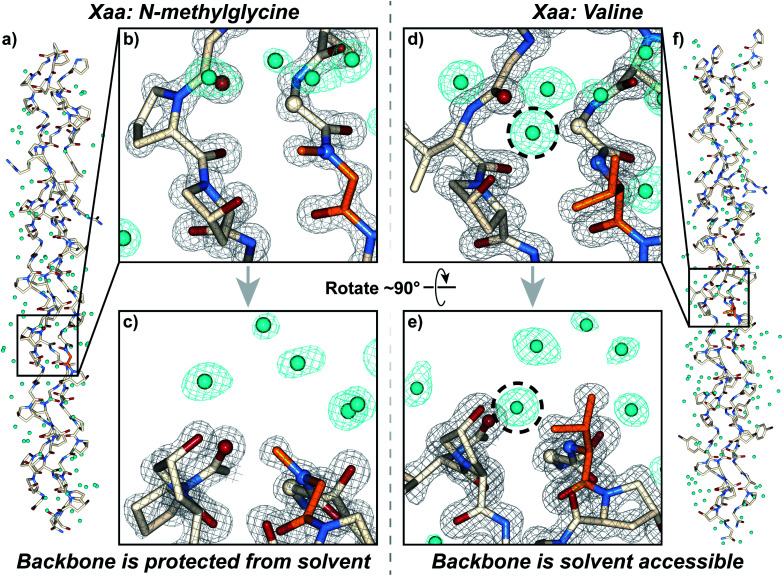
Crystal structures of CMPs **24** (PDB: 6W46) and **25** (PDB: 6W47) solved to 1.25 Å and 1.15 Å resolution, respectively. (a) Full triple helix structure of CMP **25**, with water molecules visible. (b) Close-up, head-on view of the local region around an Xaa position *N*-methylglycine residue (orange) with electron density maps visible. (c) Close-up, side view of the local region around the *N*-methylglycine residue. (d) Close-up, head-on view of the local region around an Xaa position valine residue (orange) with electron density maps visible. (e) Close-up, side view of the local region around the valine residue. (f) Full triple helix structure of CMP **24**, with water molecules visible. In all views, water molecules and their electron density are colored aqua. In views (b–e), the electron density map (2mF0-DFc) is contoured at 1.5 *σ* for the peptide and at 1.0 *σ* for water molecules. In views (b–e), some of the peptide chains and water molecules are hidden for clarity. In views (d) and (e), the dashed circle indicates the water molecule capable of participating in H-bond interactions with the peptide backbone in the local region of the Xaa residue; notably, this specific water molecule is absent when the Xaa position is occupied by *N*-methylglycine as seen in (b) and (c).

We synthesized and crystallized two peptides with either Val (CMP **24**, PDB: 6W46, Table S1†) or NmetG (CMP **25**, PDB: 6W47, Table S2†) in the Xaa position to gain structural understanding of these results (Fig. 4a and f). Val was chosen due to the substantial decrease in triple-helical thermal stability observed when paired with azGly and NmetG was chosen due to the increase in thermal stability observed when paired with azGly. A (PRG) triplet was incorporated in both CMPs, as our laboratory has found its inclusion to assist with crystal formation. For one CMP we included a 4-bromophenylalanine (BrF) residue for phasing using single-wavelength anomalous diffraction, but molecular replacement was successful for both structures. We solved the structure of CMP **24**, bearing the Xaa position Val residue, with the sequence [H–(POG)_2_(PRG)(POG)_2_(VOG)(POG)_2_(BrF–OG)(POG)–NH_2_] to 1.25 Å resolution. We solved the structure of the CMP **25**, bearing the Xaa position NmetG residue, with the sequence [H–(POG)_2_(PRG)(POG)_3_(NmetG–OG)(POG)_2_–NH_2_] to 1.15 Å resolution.

Analysis of these structures demonstrates the solvent accessibility of the peptide backbone is dependent on the Xaa residue (Fig. 4d and e). In the structure with the Xaa position Val residue, a void is present along the surface of the triple helix at the site of Val incorporation (Fig. 4d and e). This is a result of Val's secondary backbone amine being sterically unencumbered compared to the tertiary backbone amine of *N*-substituted residues like Pro, Hyp, or peptoids. The consequence of this hole is solvent exposure, clearly indicated the presence of a water molecule next to the Val residues in our structure (Fig. 4d and e). This water molecule is well-positioned to hydrogen bond with the NH group of the Val residue at approximately 3.0 Å away. Importantly, this water is also positioned in close proximity with the Cα of the Gly residue adjacent to the Xaa Val approximately 3.3 Å away. This Gly Cα corresponds to the azGly substitution site in our model system. In the NmetG structure, the void in space observed along the surface of the triple helix in the Val structure is filled by the *N*-methyl group of NmetG (Fig. 4b and c). This prevents the NH backbone–water interaction from occurring and better protects the Gly Cα from solvent exposure, with the nearest water being positioned approximately 3.7 Å away from the backbone NH and approximately 3.5 Å away from the Gly Cα.

Reviewing available structures of collagen triple helices in the PBD reveals similar behaviors. We analyzed ∼50 structures with varied sequences, containing a variety of residues in the Xaa position. Across all structures, it is consistently observed when the Xaa position is not Pro or Hyp the backbone N is able to participate in hydrogen bond interactions with solvent, with a water molecule positioned an average of 3.1 ± 0.2 Å away. When a Pro or Hyp is present, the backbone N is unable to participate in hydrogen bonding and the nearest water molecule is forced further away, at an average distance of 3.8 ± 0.1 Å. Similarly, when the Xaa position is not Pro or Hyp the solvent accessibility of the adjacent Gly Cα increases as well. Across all structures, if the Xaa position is either Pro or Hyp the average distance to the nearest water molecule for any given Gly Cα that is not located at either terminus is 3.9 ± 0.1 Å or 3.9 ± 0.2 Å, respectively. If there is any other residue present in the Xaa position, this average drops to 3.4 ± 0.2 Å. These observations are consistent with our structures, with the NmetG residue blocking solvent to a greater extent than the Val residue. As previously mentioned, we hypothesize that the loss or gain in triple-helical thermal stability observed with our Xaa CMP library is, in part, dependent on the ability of a specific Xaa residue to block solvent. If azGly is present in the adjacent position and the Xaa residue is unable to block solvent, azGly's ability to form productive interstrand H-bonds will be disturbed as solvent interactions occur. This behavior would explain the loss in triple-helical thermal stability observed upon azGly incorporation. Conversely, if the Xaa residue is able to block solvent, azGly's ability to form productive interstrand H-bonds will be enhanced, and triple-helical thermal stability will increase with azGly incorporation.

**Fig. 5:**
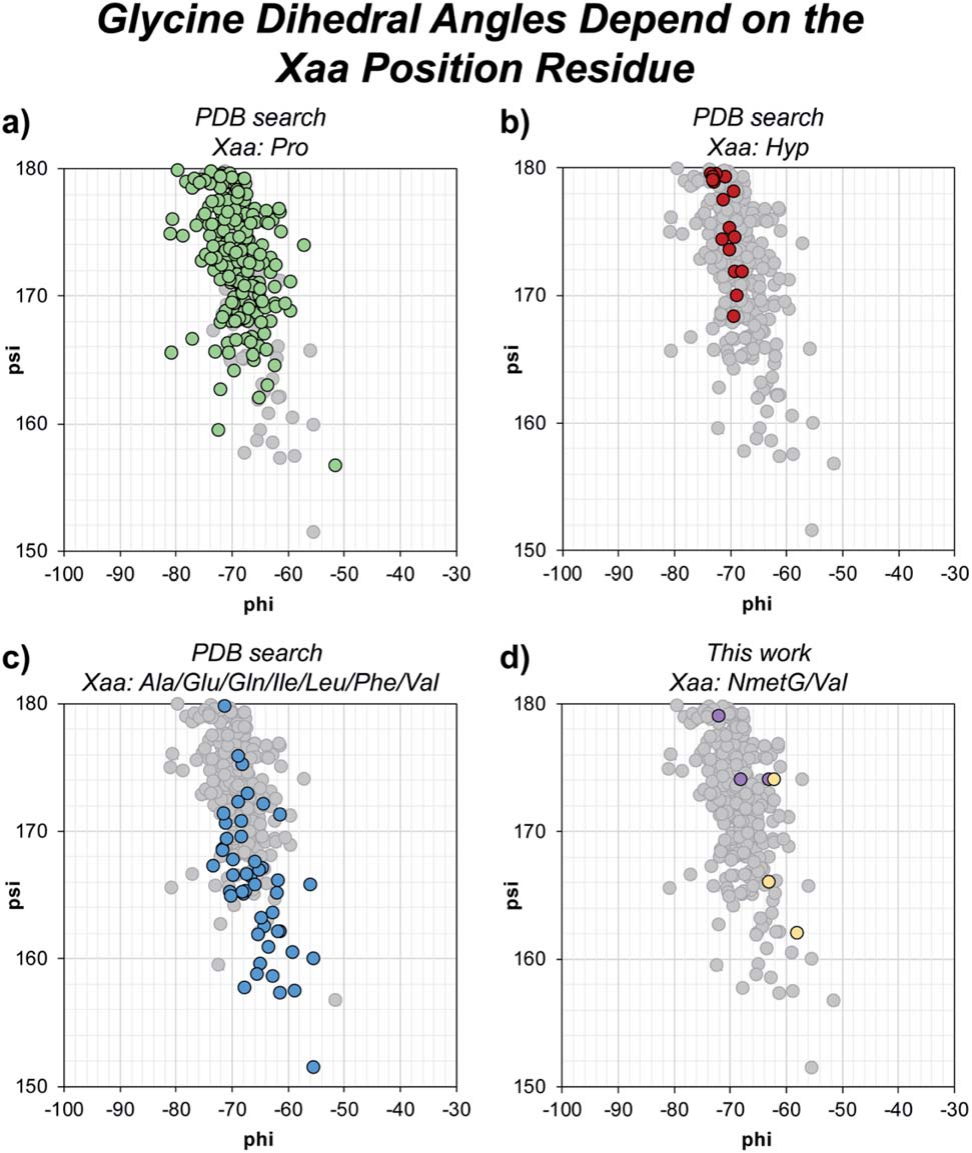
Dihedral angles of glycine residues grouped by the identity of the adjacent Xaa residue. The view area is restricted to the given region for clarity. The following PDB models were examined: 1BKV, 2DRT, 2DRX, 3A08, 3A19, 3ADM, 3P46, 4DMT, 4GYX, and 6A0C. (a) Highlighted data points for Gly residues in –(Gly–Pro)– regions (green). (b) Highlighted data points for Gly residues in –(Gly–Hyp)– regions (red). (c) Highlighted data points for Gly residues in –(Gly–Ala)–, –(Gly–Glu)–, –(Gly–Gln)–, –(Gly–Ile)–, –(Gly–Leu)–, –(Gly–Phe)–, or –(Gly–Val)– regions (blue). (d) Highlighted data points for the Gly residues in our structures, 6W46 and 6W47, in –(Gly–NmetG)– regions (purple) or –(Gly–Val)– regions (yellow).

Beyond solvent exposure, the identity of the Xaa position residue will affect the backbone dihedral angles of nearby residues. Averaging *φ* angles of Gly residues from collagen structures in the PDB gives a value of −69 ± 5° while averaging *ψ* angles gives a value of −176 ± 5° or 173 ± 5°.^32^ In our structure, the Gly residues adjacent to the Xaa NmetG residue adopt conformations closely matching the PDB average Gly (*φ*, *ψ*) angles with average values of (−68°, 176°) (Fig. 5d and Table S4†). Comparatively, the Gly residues adjacent to the Val residue adopt conformations with average (*φ*, *ψ*) angles of (−61°, 167°), deviating more from the global average (Fig. 5d and Table S3†). Comparing these angles to other structures in the PDB, the Gly residues next to NmetG adopt conformations similar to those adopted by Gly residues adjacent to either Pro or Hyp residues (Fig. 5a and b). When the Xaa position is a not an *N*-substituted residue, then the adjacent Gly residue is slightly twisted out of this conformation, which may be critical when azGly is present in this position (Fig. 5c). This backbone perturbation could have a profound effect on the azGly residue's ability to form favorable interactions and strong H-bonds. We recently reported the potential energy surfaces for model systems corresponding to collagen-like peptides to determine the energetic barriers for rotation.^40^ These models consisted of either a Gly or an azGly residue adjacent to what corresponded to either an *N*-substituted residue or a *N*-unsubstituted residue, demonstrating that azGly residues are more conformationally constrained than Gly residues. Compared to the average dihedral angle values of (−69°, 173°) or (−69°, −176°) observed with Gly residues in collagen structures, azGly residues prefer dihedral angles of (−65°, 167°) when adjacent to a secondary-like amino acid and angles of (−62°, 159°) when adjacent to *N*-unsubstituted residues. Placing an azGly residue adjacent to an *N*-unsubstituted residue forces the azGly to deviate more strongly from the “ideal” collagen Gly residue dihedral angles than if the azGly residue was incorporated next to a residue like Pro, Hyp, or NmetG. The conformational bias imposed by the Xaa position residue, combined with the level of local backbone solvent exposure, points to the underlying explanation of azGly's preference for *N*-substituted Xaa residues.

### The principles and guidelines for aza-glycine incorporation

**Fig. 6:**
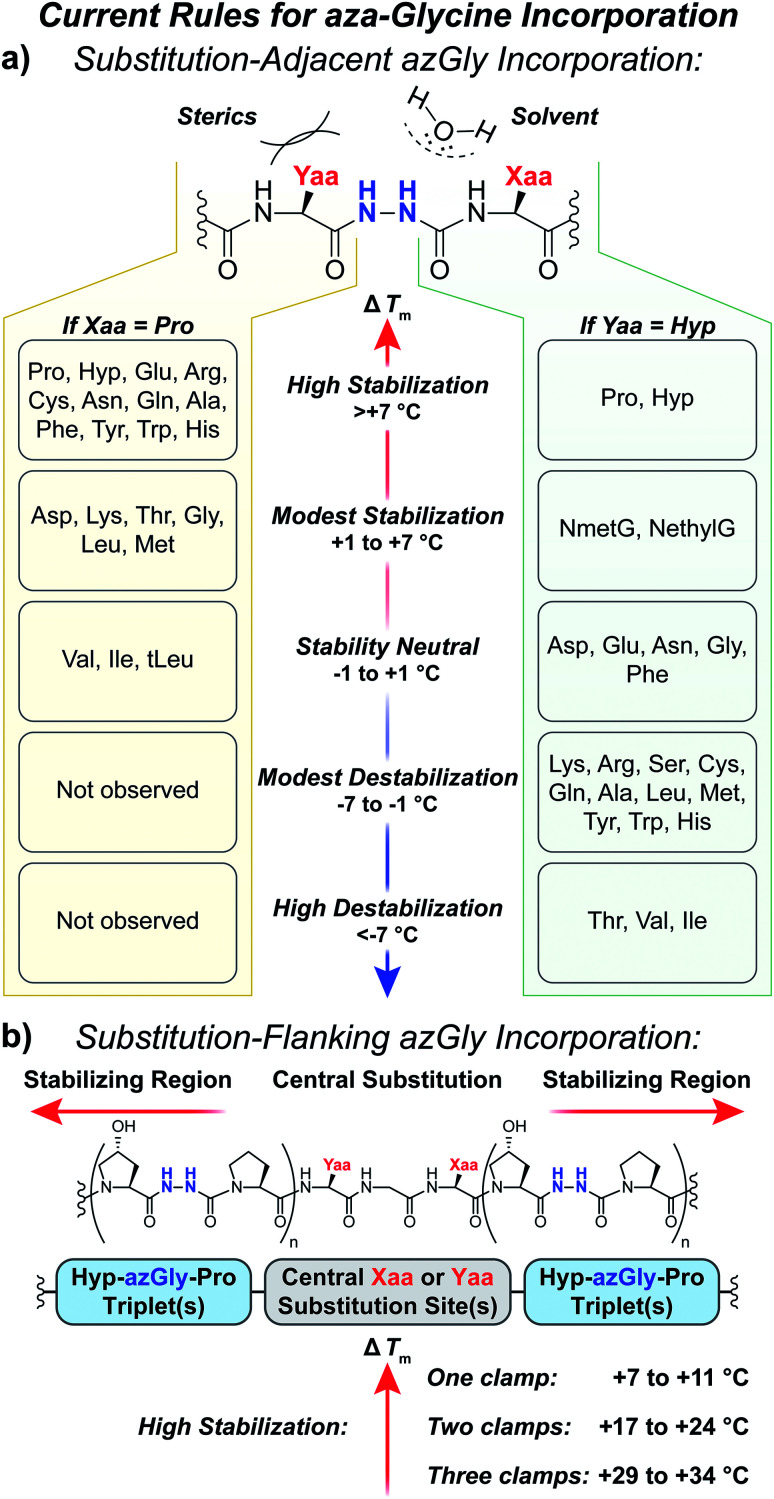
(a) Summary of aza-glycine's effect on triple-helical thermal stability (Δ*T*_m_) within our model [H–(POG)_8_–NH_2_]-like system, incorporated adjacent to either a central Yaa^33^ or Xaa substitution. The Δ*T*_m_ value is given as the difference between the azGly-containing CMP and the corresponding control (*e.g.* CMPs **10b** and **10a**). Residues are categorized by the observed Δ*T*_m_ upon azGly incorporation. Left: Yaa position substitutions when the Xaa position is Pro. *t*Leu refers to the unnatural amino acid l-*tert*-leucine. Right: Xaa substitutions when the Yaa position is Hyp. (b) Summary of aza-glycine's effect when substituted in regions flanking a central substitution.

Our results provide a set of rules and guidelines regarding how the incorporation of azGly residues will affect the triple-helical thermal stability of a given collagen peptide (Fig. 6). When choosing potential azGly substitution sites for increasing triple-helical thermal stability, the ideal candidates include essentially any Gly residue that is positioned adjacent to an Xaa position occupied by a secondary amino acid or a peptoid residue (assuming the adjacent Yaa position is occupied by a Hyp residue). If the Xaa position does not bear an *N*-substitution, azGly incorporation will typically result in modest destabilization of the peptide. Substitution of Gly residues adjacent to Xaa positions occupied by β-branched residues should be avoided as this will result in significant triple-helical thermal stability loss (Fig. 6). Conversely, the identity of the Yaa position residue adjacent to the potential azGly substitution site is not as critical (assuming the adjacent Xaa position is occupied by a Pro residue). As seen in Fig. 6, the incorporation of azGly will, at worst, have no effect on the triple-helical thermal stability (as observed with β-branched residues like Val or Ile).^33^ Incorporation of azGly residues next to most amino acids in the Yaa position resulted in modest to high increases in triple-helical thermal stability.

The standard triplet used for small collagen mimetic peptides, (Pro–Hyp–Gly), is particularly well suited for azGly substitution. We've previously demonstrated the synergistic effect of multiple azGly residue incorporation; as the number of (Pro–Hyp–azGly) triplets increases the triple-helical thermal stability gain per azGly residue increases as well.^31^ This triplet, often used in the literature for small triple-helical collagen peptides, can be exploited to stabilize collagen peptides with central regions bearing varied sequences that would either exhibit small increases or even decreases in triple-helical thermal stability upon azGly incorporation. Substitution of Gly residues in (Hyp–Gly–Pro) triplets, when positioned in regions flanking a central substitution site, will provide a substantial increase in triple-helical thermal stability regardless of the central region's sequence. The viability of this approach can be observed both in this work with CMPs **6c**, **14c**, and **20c** and our previous work.^33^ This strategy facilitates the preparation of incredibly stable and short collagen peptides with different binding motifs and sequences that are still capable of triple helix assembly.

## Conclusions

Incorporation of azGly in collagen peptides has a diversity of consequences depending on the nature of the adjacent amino acids in both the Xaa and Yaa position. Understanding the principles for azGly incorporation is essential for designing new azGly-containing collagen peptides, as the neighboring residues in no small part affect the degree of increase or decrease in triple-helical thermal stability caused by the incorporation of azGly (Fig. 6a). Unlike the results with different Yaa position substitutions where most residues exhibited a small to moderate increase in thermal stability when paired with azGly, most residues in the Xaa position substitutions resulted in minor to moderate destabilization of the triple helix when paired with azGly. Notably, the CMPs containing Xaa position β-branched residues are particularly destabilized when paired with an adjacent azGly. Our general sequence independent strategy provides a general method for triple-helical stabilization across a broad set of collagen peptides with different central substitutions (Fig. 6b). In addition, we have reported two new collagen crystal structures, with the first structure of a collagen triple helix containing a peptoid residue. These structures, containing either a Val or NmetG residue in the Xaa position and reveal how the NmetG residues protect the collagen backbone from solvent while the Val residues expose it. These crystal structures point to the importance of preserving collagen's triple-helical backbone geometry and protecting the triple helix's hydrogen bond network from solvent. These principles are particularly relevant when incorporating azGly residues, as azGly's beneficial impact on triple-helical thermal stability depends, in no small part, on forming interstrand H-bonds. Using these new findings, and taken together with our previous work, we have established the first comprehensive set of principles and guidelines for the incorporation of azGly residues in collagen peptides.

## Experimental procedures

Crystallographic data for CMPs **24** and **25**, including atomic coordinates and structure factors, are deposited in the Protein Data Bank (PDB) with accession codes 6W46 and 6W47, respectively. Crystal data integration was performed with XDS^41^ and iMOSFLM^42^ (ver 7.2.2). Space group validation and data reduction was performed using Aimless^43^ (ver 0.7.4) in the CCP4 (ref. 44) (ver 7.0.078) software suite. Molecular replacement was performed using Phaser.^45^ Crystallographic restraints files were generated using eLBOW.^46^ Refinement was performed using Phenix^47^ (ver 1.18rc2-3794). Manual model building was performed using Coot^48^ (ver 0.8.9.2).

## Conflicts of interest

There are no conflicts to declare.

## Supplementary Material

SC-011-D0SC03003A-s001
