# Supplementary material for: Rules for the design of aza-glycine stabilized triple-helical collagen peptides
Source: Chem Sci. 2020 Jul 21;11(39):10638–46. doi: 10.1039/d0sc03003a (PMC8162259; doi:10.1039/d0sc03003a)
Supplement: SC-011-D0SC03003A-s001 [file SC-011-D0SC03003A-s001.pdf]

Electronic Supplementary Material (ESI) for Chemical Science.
This journal is © The Royal Society of Chemistry 2020

SUPPORTING INFORMATION

Rules for the Design of Aza-Glycine Stabilized Triple-Helical Collagen Peptides

Samuel D. Melton, Emily A. E. Brackhahn, Samuel J. Orlin, Pengfei Jin, and David M. Chenoweth*

Department of Chemistry, University of Pennsylvania, 231 South 34th Street, Philadelphia, Pennsylvania 19104-6323, United States

Table of Contents

Instrumentation S02

Reagents.......... S03

Synthesis, Purification, Sample Preparation, and CD Measurements.......... S04 – S05

Crystallography S06

Supporting Figures .... S07 – S12

MALDI-TOF MS Results, Thermal Denaturation Curves, and HPLC Traces.......... S13 – S61

References .... S62 – S63

ABBREVIATIONS

Boc	tert-Butyloxycarbonyl
CD	Circular dichroism
CDT	1,1′-Carbonyl-di-(1,2,4-triazole)
CHCA	α-Cyano-4-hydroxycinnamic acid
CMP	Collagen model peptide
COMU	(1-Cyano-2-ethoxy-2-oxoethylidenaminooxy)dimethylamino-morpholino-carbenium hexafluorophosphate
DBU	1,8-Diazabicyclo[5.4.0]undec-7-ene
DCM	Dichloromethane
DMF	*N*,*N*-Dimethylformamide
Fmoc	9-Fluorenylmethoxycarbonyl
HOBt	1-Hydroxybenzotriazole
HPLC	High-performance liquid chromatography
MALDI-TOF MS	Matrix-assisted laser desorption/ionization time-of-flight mass spectrometry
PBS	Phosphate-buffered saline
RT	Room temperature
SPPS	Solid-phase peptide synthesis
tBu	tert-Butyl
TFA	Trifluoroacetic acid
TIPS	Triisopropylsilane
TMP	2,2,6,6-Tetramethylpiperidine
Trt	trityl

INSTRUMENTATION

All peptides were purified using preparative or semi-preparative reversed-phase HPLC on an Agilent 1260 Infinity II system using a Phenomenex Gemini 5 µm NX-C18 110 Å LC column or a Phenomenex Luna Omega 5 µm PS C18 100 Å LC column. Varying gradients of acetonitrile and 0.1% TFA in H_2O were used depending on the individual CMP. Analytical HPLC (spectra shown for each peptide) to check purity was carried out on an Agilent 1260 Infinity II system using a Phenomenex Gemini 5 µm NX-C18 100 Å LC column. Mass spectrometry was performed using a Bruker MALDI-TOF MS Ultraflex III mass spectrometer and CHCA as the matrix. Peptides were either lyophilized using a Labconco FreeZone Plus 12 Liter Cascade Console Freeze Dry system or concentrated using rotary evaporation. UV-Vis absorption spectrophotometry was performed using a Jasco V-650 Spectrophotometer equipped with a PAC-743R multichannel Peltier and 1 cm path length quartz cells. Circular dichroism experiments were performed using a Jasco J-1500 Circular Dichroism Spectrometer and 1 mm quartz cuvettes.

REAGENTS

All commercially available reagents were used as received. Rink Amide AM Resin was purchased from Novabiochem. CDT and COMU were purchased from Chem-Impex Int'l. Inc. CHCA, DIEA, DMF, and ether were purchased from Sigma-Aldrich. 10X PBS stock solution (sln) was purchased from Fisher. DBU and TFA were purchased from Acros Organics. 9-Fluorenylmethyl carbazate was purchased from Oakwood Chemical. HOBt was purchased from EMD Millipore.

Fmoc-Amino Acids:	Supplier:
Fmoc-Pro-OH	Chem-Impex Int'l. Inc.
Fmoc-Hyp(tBu)-OH	Ark Pharm, Inc.
Fmoc-Gly-OH	Chem-Impex Int'l Inc.
Fmoc-Asp(tBu)-OH	Chem-Impex Int'l. Inc.
Fmoc-Glu(tBu)-OH	Novabiochem
Fmoc-Lys(Boc)-OH	Novabiochem
Fmoc-Ser(tBu)-OH	Chem-Impex Int'l. Inc.
Fmoc-Thr(tBu)-OH	Novabiochem
Fmoc-Cys(Trt)-OH	Novabiochem
Fmoc-Asn(Trt)-OH	Novabiochem
Fmoc-Gln(Trt)-OH	Novabiochem
Fmoc-Ala-OH	Novabiochem
Fmoc-Val-OH	Chem-Impex Int'l. Inc.
Fmoc-Leu-OH	Novabiochem
Fmoc-Ile-OH	Chem-Impex Int'l. Inc.
Fmoc-(t-Leu)-OH	Ark Pharm, Inc.
Fmoc-Met-OH	Chem-Impex Int'l. Inc.
Fmoc-Phe-OH	Novabiochem
Fmoc-Trp(Boc)-OH	Novabiochem
Fmoc-Tyr(tBu)-OH	Chem-Impex Int'l. Inc.
Fmoc-His(Trt)-OH	Novabiochem
Fmoc-*N*-methylglycine-OH	Chem-Impex Int'l. Inc.
Fmoc-*N*-ethylglycine-OH	Acrotein
Fmoc-4-bromo-L-phenylalanine-OH	AstaTech Inc.

Fmoc-Pro-Hyp(tBu)-Gly-OH was synthesized using methods previously described by our group[1,2].

SYNTHESIS, PURIFICATION, SAMPLE PREPARATION, AND CD MEASUREMENTS

a. Resin Preparation

All peptides were synthesized using manual SPPS. All peptides in this study were prepared on a 0.0125 mmol (1 equivalent) scale on Rink Amide MBHA resin LL (loading density: 0.36 mmol/g). The resin was swelled in DMF for 30 min. All Fmoc group deprotections were performed using 2 mL of a 1% HOBt (w/v), 2% DBU (v/v) in DMF solution and stirring for 1-2 min, repeated twice. The resin was then thoroughly washed with DMF. Finally, a coupling solution containing Fmoc-Pro-Hyp(tBu)-Gly-OH (described below) was added to the resin and stirred for 45 min at 60 ˚C. The solution was then drained, the resin was washed with DMF, the Fmoc group on the growing peptide chain was removed as previously described, and the resin was washed with DMF to give H-(PO(tBu)G) on resin. For all CMPs except **6c**, **14c**, and **20c** this POG coupling was repeated two times to give H-$(POG)_3$ on resin, and followed by standard amino acids couplings (described below) with Fmoc-Gly-OH and Fmoc-Hyp(tBu)-OH to give H-(OG)$(POG)_3$ on resin. For CMPs **6c**, **14c**, and **20c** only one POG coupling was performed.

b. Standard Amino Acid Couplings

Following Fmoc deprotection and washing, amino acid coupling solutions were added to the free amine on the growing peptide chain. The coupling solutions contained 5 equivalents of the appropriate Fmoc-amino acid (listed above), 5 equivalents of COMU, and 7.5 equivalents of TMP in 1 mL of DMF. Prior to addition to resin, the coupling solutions were allowed to sit for 5-10 min. All couplings were done over 45 min at 60 ˚C with the exception of His, Met, and Cys couplings which were done at RT. The coupling sln was then drained, and the resin was then washed with DMF. The Fmoc group was removed as described above and the resin was washed again with DMF.

c. Fmoc-Pro-Hyp(tBu)-Gly-OH Coupling

The amino acid trimer Fmoc-Pro-Hyp(tBu)-Gly-OH coupling solution was prepared in the same manner as the single amino acids. The coupling solution contained 5 equivalents of Fmoc-Pro-Hyp(tBu)-Gly-OH, 5 equivalents of COMU, and 7.5 equivalents of TMP in 1 mL of DMF. Prior to addition to resin, the coupling solution was allowed to sit for 5-10 min. The coupling solution was stirred with the resin for 45 min at 60 ˚C before being drained. The resin was then washed with DMF, the Fmoc group was removed using 1 mL of a 1% HOBt (w/v), 2% DBU (v/v) in DMF and stirring for 1 min (x3), and the resin was washed again with DMF. Whenever possible and appropriate, POG coupling solutions were used while synthesizing each CMP.

d. Aza-Glycine Couplings and Subsequent Amino Acid Couplings

Ten equivalents of 9-Fluorenylmethyl carbazate and ten equivalents of CDT were combined in 1 mL of DMF and activated at RT for 5-10 min before being added to the growing peptide chain on resin. The solution and resin were stirred for 24 hr before draining the coupling solution and washing the resin with DMF. The Fmoc group was removed using 1 mL of a 1% HOBt (w/v), 2% DBU (v/v) in DMF and stirring for 1 min (x3), and the resin was washed again with DMF. Following the azGly coupling, a Fmoc-Hyp(tBu)-OH coupling was performed followed by a Fmoc-Pro-OH coupling (as described above) to give H-(POazG)(XOG)$(POG)_3$ on resin, where "X" represents any given amino acid.

e. *Cleavage from Resin and Precipitation*

Following all appropriate couplings, the final Fmoc group was removed using 1 mL of a 1% HOBt (w/v), 2% DBU (v/v) in DMF and stirring for 1 min (x3), and the resin was washed first with DMF and then with DCM. A 2 mL cleavage cocktail containing 95% TFA, 2.5% TIPS, and 2.5% H_2O was added to the resin. The mixture was stirred for 2 hr before being collected into cold ether, causing the peptide to precipitate. The solid was collected by centrifugation, resuspended in cold ether, and collected by centrifugation again (3x). The final pellet was then dissolved in 1.5 mL of 50:50 H_2O:CH_3CN and stored at 4 ˚C prior to purification.

f. *Purification*

The peptide solutions were purified using semi-preparative reversed-phase HPLC using acetonitrile and 0.1% TFA in H_2O. During purification, the column was heated at 60 ˚C to prevent triple helix formation and aid in separation. The absorbance at 215 nm was monitored to determine collection, collected fractions were analyzed using MALDI-TOF MS in positive ion mode. Appropriate fractions were combined and lyophilized to yield the desired peptide as a white solid. Purity was then checked using analytical HPLC, with the column heated at 60 ˚C. Please note that in many of the HPLC traces (below), there are peaks that appear around a 3-4 min retention time. These peaks are solvent artifacts resulting from differences in the sample solvent and the HPLC system solvent upon injection, as some of the samples were dissolved in 1X PBS prior to checking purity via analytical HPLC.

g. *Sample Preparation*

After obtaining the pure product, each peptide was then dissolved in a small amount of 1X pH 7.4 PBS. Using UV-Vis spectrophotometry, the concentration of each sample was determined by measuring the absorbance at 214 nm and using an extinction coefficient of 60 $mM^{-1}cm^{-1}$ as described by Engel *et al.*[3]. The stock solution was then diluted using PBS to a final concentration of 0.2 mM and stored at 4 ˚C for 24 hr before carrying out CD measurements.

h. *CD Measurements and T_m Determination*

For each peptide, approximately 200 µL of the 0.2 mM solution was placed into a 1 mm quartz cuvette. The ellipticity of these solutions was then measured from 260 to 190 nm while holding the temperature at 4 ˚C. Measurements were obtained in triplicate and then converted to mean residue ellipticity and averaged to generate the CD scan curves included for each peptide below. Following this, the solutions were then heated at a rate of 12 ˚C/hr starting at 5 ˚C and ending at 92 ˚C while monitoring the absorbance at 210, 215, 220, and 225 nm. These measurements, obtained in triplicate, were converted to mean residue ellipticity and averaged. The melting temperate for each peptide, T_m, was determined by using the program GraphPad Prism 7 by fitting the data to a two-state model to find the temperature at which 50% of starting ellipticity was lost, as described previously[1].

CRYSTALLOGRAPHY

a. *Crystallization*

Peptide stock concentrations were determined using UV-Vis. The absorption was measured at 214 nm and used an extinction coefficient of 60 mM^{-1} cm^{-1} was used.

CMP 24. Peptide **24** was crystallized using sitting-vapor drop diffusion under conditions adapted from Okuyama *et. al*.[4] The peptide stock was prepared at a concentration of 12 mg/mL. Crystal trials were performed using 1 µL of the peptide solution and 1 µL of the reservoir solution of 0.1 M $Li_2SO_4 \cdot H_2O$, 0.094 M Tris-HCl, and 30% (w/v) PEG 4000 at pH 7.6. Trays were incubated at RT for two months. Prior to beamline analysis, crystals were dipped in a drop of cryoprotectant mixture consisting of 20% glycerol mixed with 80% reservoir solution.

CMP 25. Peptide **25** was crystallized using the PEG/Ion HT screen purchased from Hampton Research. The peptide stock was prepared at a concentration of 10 mg/mL. Crystal trials were performed using 200 nL of the peptide solution and 200 nL of the reservoir solution of 0.2 M potassium formate and 20% PEG 3350 at pH 7.3. Trays were incubated at 4 ˚C for approximately three months before being moved to RT for two months. Prior to beamline analysis, crystals were dipped in a drop of cryoprotectant mixture consisting of 20% glycerol mixed with 80% reservoir solution.

b. *Data Collection, Refinement, and Analysis*

Crystal data integration was performed with XDS[5] and iMOSFLM[6] (ver 7.2.2). Space group validation and data reduction was performed using Aimless[7] (ver 0.7.4) in the CCP4[8] (ver 7.0.078) software suite. Molecular replacement (MR) was performed using Phaser[9]. MR search models consisted of full length triple helical collagen structures and truncated triple helical collagen structures modified to be short $(Ala\text{-}Ala\text{-}Gly)_n$ sequences. Crystallographic restraints files for *N*-methylglycine, 4-bromophenylalanine, and the C-terminal amidated glycine residues were generated using eLBOW[10]. Refinement was performed using Phenix[11] (ver 1.17.1-3660). Manual model building was performed using Coot[12] (ver 0.8.9.2).

c. *PDB Analysis*

The comprehensive list of all structures examined is as follows: 1A3I, 1A3J, 1BKV, 1CAG, 1CGD, 1EI8, 1G9W, 1ITT, 1K6F, 1Q7D, 1QSU, 1V4F, 1V6Q, 1V7H, 1X1K, 2CUO, 2D3F, 2D3H, 2DRT, 2DRX, 3A0A, 3A0M, 3A1H, 3A08, 3A19, 3ABN, 3ADM, 3AH9, 3B0S, 3B2C, 3DMW, 3P46, 3POD, 3PON, 3T4F, 3U29, 3WN8, 4AXY, 4DMT, 4GYX, 4OY5, 4Z1R, 5K86, 5Y46, 6A0C, 6HG7, 6JEC, and 6JKL. These structures were chosen as they contained only the collagen triple helix in their crystal structure and present a variety of primary sequences. Within each structure, the distance between the nearest water molecule to the Cα of every glycine residue and the backbone N of every Xaa position residue was measured using the UCSF Chimera software package.[13] Additionally, UCSF Chimera was used to measure the phi and psi angles for each structure.

SUPPORTING FIGURES

Beamline	NSLS-II 17-ID-1 (AMX)
Detector	Dectris Eiger 9M
Wavelength (Å)	0.91976
Resolution range (Å)	37.24 - 1.25 (1.295 - 1.25)
Space group	$P\ 2_1$
Unique reflections	16476 (1611)
Unit cell	
(*a*, *b*, *c*; Å)	37.300, 19.680, 40.810
(*α*, *β*, *γ*; °)	90.00, 93.26, 90.00
Multiplicity	2.9 (3.0)
Completeness (%)	97.48 (96.44)
Mean I/σ_I	5.7 (1.7)
Wilson B-factor (Å^2)	8.83
R_{merge}	0.115 (1.081)
R_{meas}	0.141 (1.314)
R_{pim}	0.080 (0.736)
$CC_{1/2}$	0.994 (0.574)
Reflections used in refinement	16381 (1598)
Reflections used for R_{free}	1633 (157)
R_{work}	0.1874 (0.2775)
R_{free}	0.2267 (0.3227)
Number of non-hydrogen atoms	793
Macromolecules	610
Ligands	31
Solvent	152
Amino acid residues	90
RMSD	
Bonds (Å)	0.01
Angles (°)	1.34
Ramachandran favored (%)	100
Ramachandran allowed (%)	0
Ramachandran outliers (%)	0
Rotamer outliers (%)	0
Clashscore	0.79
Average B-factor (Å^2)	16.2
Macromolecules	13.59
Ligands	38.9
Solvent	22.07

Table S1. Crystallography table for CMP **24** (PDB accession code: 6W46).

Beamline	APS 24-ID-C
Detector	DECTRIS PILATUS 6M-F
Wavelength (Å)	0.97918
Resolution range (Å)	31.3 - 1.15 (1.191 - 1.15)
Space group	$P\ 2_1$
Unique reflections	15857 (1215)
Unit cell	
(*a, b, c*; Å)	31.679, 19.556, 37.528
(*α, β, γ*; °)	90.00, 98.915, 90.00
Multiplicity	4.6 (3.1)
Completeness (%)	95.75 (75.50)
Mean I/σ_I	9.4 (2.8)
Wilson B-factor (Å^2)	8.06
R_{merge}	0.107 (0.646)
R_{meas}	0.122 (0.796)
R_{pim}	0.072 (0.530)
$CC_{1/2}$	0.995 (0.823)
Reflections used in refinement	15798 (1211)
Reflections used for R_{free}	1581 (122)
R_{work}	0.1834 (0.2676)
R_{free}	0.2172 (0.2715)
Number of non-hydrogen atoms	608
Macromolecules	483
Ligands	29
Solvent	96
Amino acid residues	78
RMSD	
Bonds (Å)	0.011
Angles (°)	1.3
Ramachandran favored (%)	100
Ramachandran allowed (%)	0
Ramachandran outliers (%)	0
Rotamer outliers (%)	0
Clashscore	1.02
Average B-factor (Å^2)	13.1
Macromolecules	12.31
Ligands	15.88
Solvent	16.29

Table S2. Crystallography table for CMP **25** (PDB accession code: 6W47).

Phi Angles of PDB: 6W46 (Xaa: Val)					
Residue No.	Amino Acid	Chain A	Chain B	Chain C	Avg. All
4	Pro	-70	-69	-73	-71
5	Hyp	-56	-55	-56	-56
6	Gly	-70	-63	-67	-66
7	Pro	-64	-63	-66	-64
8	Arg	-59	-64	-62	-62
9	Gly	-70	-69	-68	-69
10	Pro	-79	-76	-76	-77
11	Hyp	-60	-55	-65	-60
12	Gly	-64	-73	-70	-69
13	Pro	-66	-71	-67	-68
14	Hyp	-63	-59	-63	-62
15	Gly	-62	-63	-58	-61
16	Val	-71	-58	-68	-66
17	Hyp	-59	-60	-60	-59
18	Gly	-65	-65	-66	-65
19	Pro	-70	-67	-75	-71
20	Hyp	-57	-61	-52	-57
21	Gly	-67	-68	-71	-69
22	Pro	-66	-62	-64	-64
23	Hyp	-58	-56	-62	-59
24	Gly	-68	-68	-66	-67
25	BrF	-71	-69	-72	-70
26	Hyp	-60	-60	-60	-60
27	Gly	-70	-68	-66	-68

Psi Angles of PDB: 6W46 (Xaa: Val)					
Residue No.	Amino Acid	Chain A	Chain B	Chain C	Avg. All
4	Pro	153	158	165	159
5	Hyp	148	146	149	148
6	Gly	176	173	171	173
7	Pro	160	157	159	159
8	Arg	145	145	146	145
9	Gly	172	172	175	173
10	Pro	166	164	167	165
11	Hyp	147	147	153	149
12	Gly	167	179	177	175
13	Pro	161	163	161	162
14	Hyp	156	151	151	153
15	Gly	174	166	162	167
16	Val	161	147	159	156
17	Hyp	146	148	146	147
18	Gly	166	162	171	166
19	Pro	156	161	165	160
20	Hyp	144	150	146	147
21	Gly	174	175	-178	57
22	Pro	163	158	156	159
23	Hyp	150	148	152	150
24	Gly	176	172	168	172
25	BrF	154	154	161	156
26	Hyp	154	151	148	151
27	Gly	173	173	171	172

Table S3. Measured phi and psi angles of CMP **24**, containing the Xaa position Val residue. The first and last three terminal residues of the CMP have been excluded to account for the one-residue stagger of the collagen triple helix and helical fraying. The Xaa position residue, Val, is highlighted in blue and the adjacent Gly residue is highlighted in green.

Phi Angles of PDB: 6W47 (Xaa: NmetG)					
Residue No.	Amino Acid	Chain A	Chain B	Chain C	Avg All
4	Pro	-60	-73	-65	-66
5	Hyp	-58	-54	-55	-56
6	Gly	-70	-67	-69	-69
7	Pro	-72	-66	-70	-69
8	Arg	-63	-62	-66	-64
9	Gly	-67	-66	-67	-66
10	Pro	-70	-72	-75	-72
11	Hyp	-55	-65	-60	-60
12	Gly	-65	-70	-74	-70
13	Pro	-69	-75	-63	-69
14	Hyp	-53	-49	-54	-52
15	Gly	-65	-66	-63	-65
16	Pro	-69	-68	-68	-68
17	Hyp	-58	-60	-60	-59
18	Gly	-72	-68	-63	-68
19	NmetG	-76	-79	-81	-79
20	Hyp	-66	-69	-67	-67
21	Gly	-65	-69	-69	-67
22	Pro	-67	-72	-63	-67
23	Hyp	-63	-57	-60	-60
24	Gly	-70	-68	-73	-70

Psi Angles of PDB: 6W47 (Xaa: NmetG)					
Residue No.	Amino Acid	Chain A	Chain B	Chain C	Avg All
4	Pro	153	163	157	157
5	Hyp	141	145	152	146
6	Gly	168	172	168	170
7	Pro	154	147	156	152
8	Arg	146	146	145	145
9	Gly	169	165	168	168
10	Pro	152	163	164	160
11	Hyp	148	157	150	152
12	Gly	170	171	177	173
13	Pro	161	164	156	160
14	Hyp	142	147	147	145
15	Gly	170	176	168	171
16	Pro	160	156	160	159
17	Hyp	150	153	150	151
18	Gly	179	174	174	176
19	NmetG	178	176	-177	59
20	Hyp	151	155	148	151
21	Gly	171	-179	176	56
22	Pro	160	163	154	159
23	Hyp	152	146	150	149
24	Gly	171	173	172	172

Table S4. Measured phi and psi angles of CMP **25**, containing the Xaa position NmetG residue. The first and last three terminal residues of the CMP have been excluded to account for the one-residue stagger of the collagen triple helix and helical fraying. The Xaa position residue, NmetG, is highlighted in blue and the adjacent Gly residue is highlighted in green.

MALDI-TOF MASS SPECTROMETRY RESULTS, THERMAL DENATURATION CURVES, AND HPLC TRACES

CMP **2a**: H-(Pro-Hyp-Gly)$_3$(Pro-Hyp-Gly)(**Hyp**-Hyp-Gly)(Pro-Hyp-Gly)$_3$-NH_2

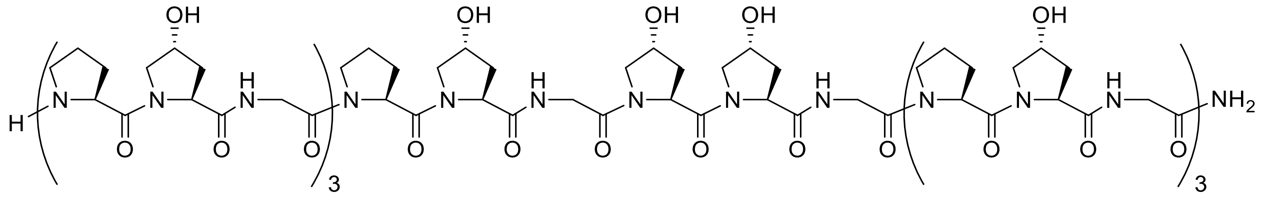

MALDI-TOF MS:

Calculated $[M+Na]^+$: 2193.990

Found: 2193.711

THERMAL DENATURATION CURVE:

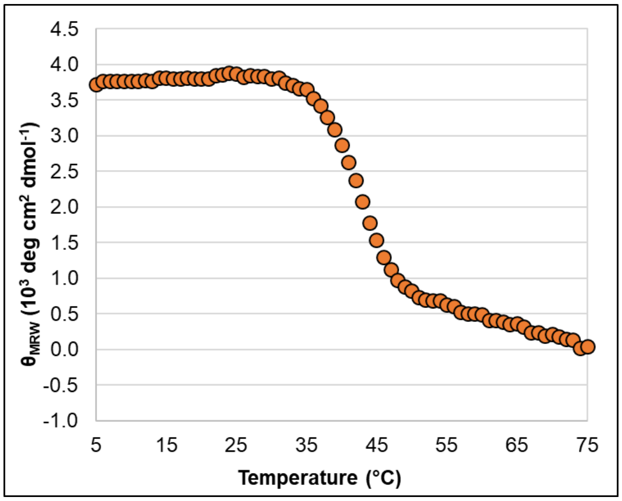

HPLC: 5-25% CH_3CN in 0.1% TFA H_2O over 30 min at 60 ˚C

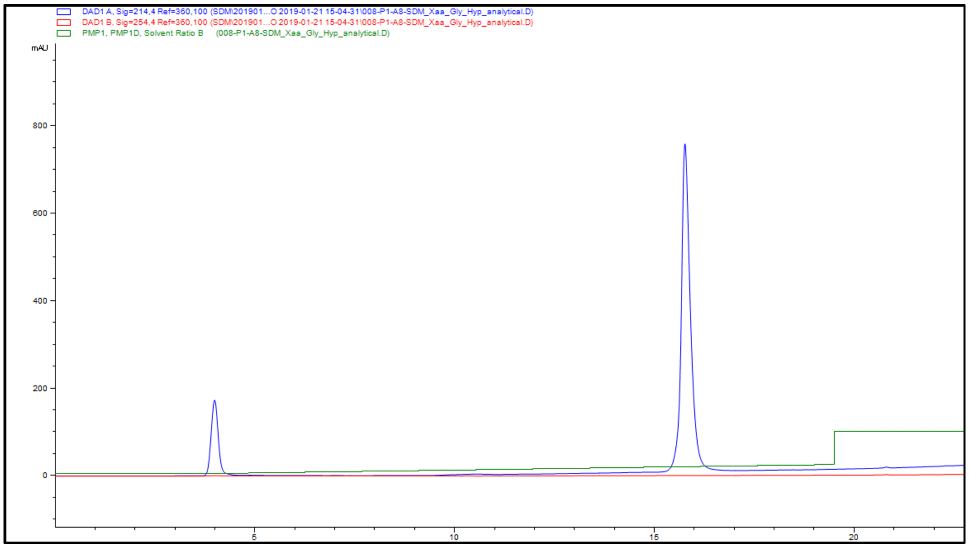

CMP **2b**: H-(Pro-Hyp-Gly)$_3$(Pro-Hyp-**azGly**)(**Hyp**-Hyp-Gly)(Pro-Hyp-Gly)$_3$-NH$_2$

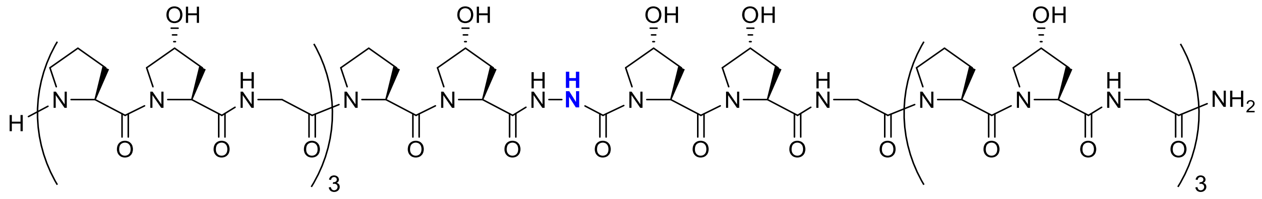

MALDI-TOF MS:

Calculated [M+Na]$^+$: 2194.985

Found: 2194.751

THERMAL DENATURATION CURVE:

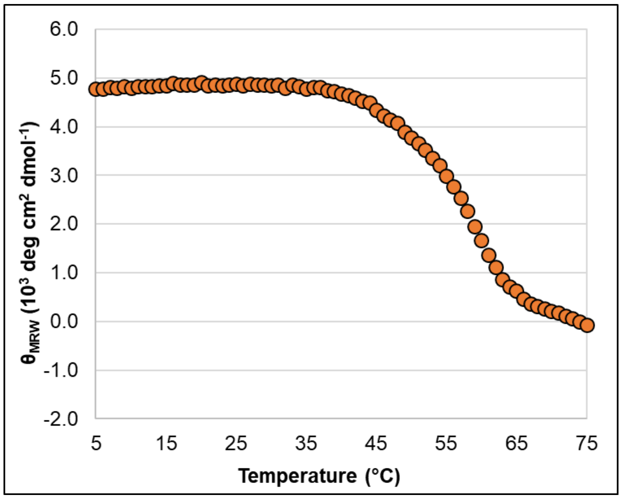

HPLC: 1-25% CH_3CN in 0.1% TFA H_2O over 30 min at 60 ˚C

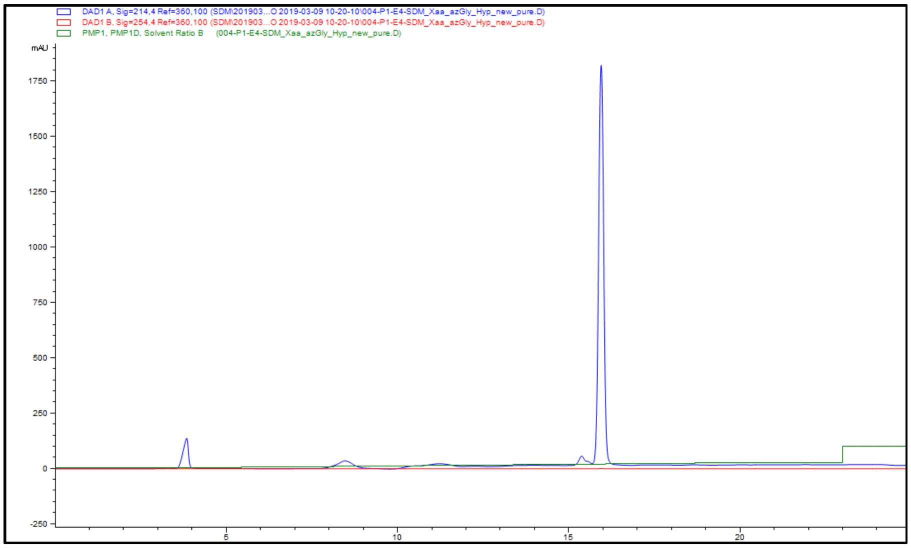

CMP **3a**: H-(Pro-Hyp-Gly)$_3$(Pro-Hyp-Gly)(**Asp**-Hyp-Gly)(Pro-Hyp-Gly)$_3$-NH$_2$

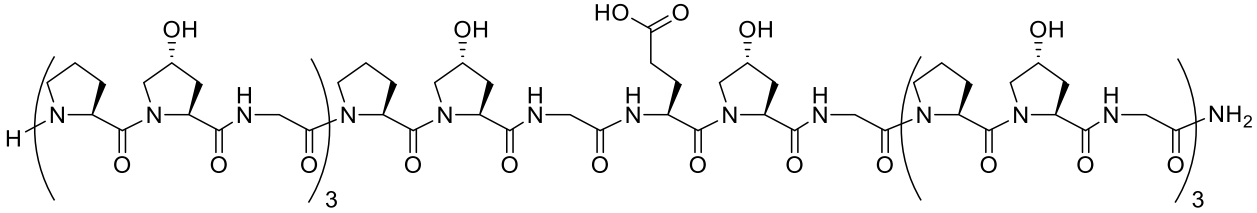

MALDI-TOF MS:

Calculated [M+Na]$^+$: 2195.969

Found: 2196.033

THERMAL DENATURATION CURVE:

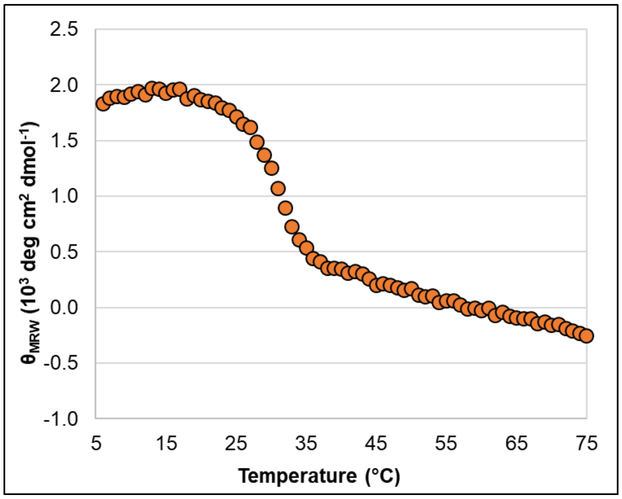

HPLC: 1-25% CH_3CN in 0.1% TFA H_2O over 30 min at 60 ˚C

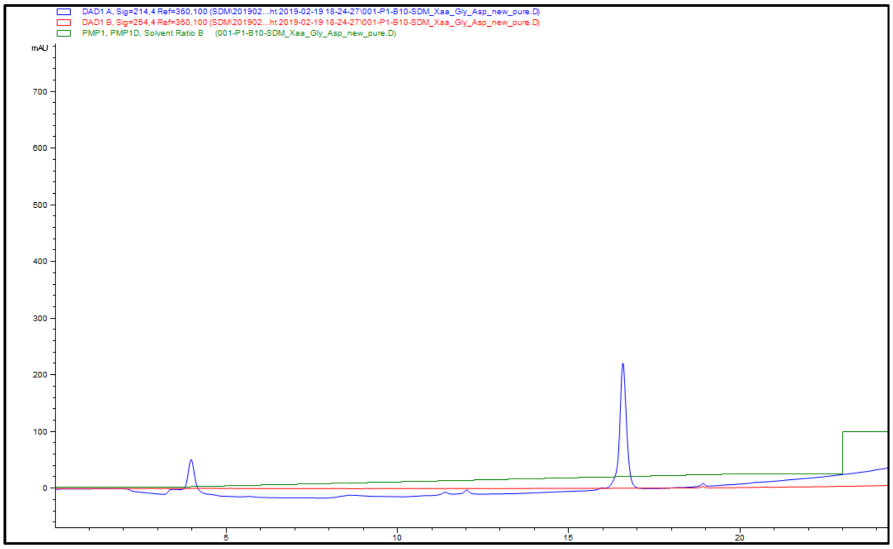

CMP **3b**: H-(Pro-Hyp-Gly)$_3$(Pro-Hyp-**azGly**)(**Asp**-Hyp-Gly)(Pro-Hyp-Gly)$_3$-NH$_2$

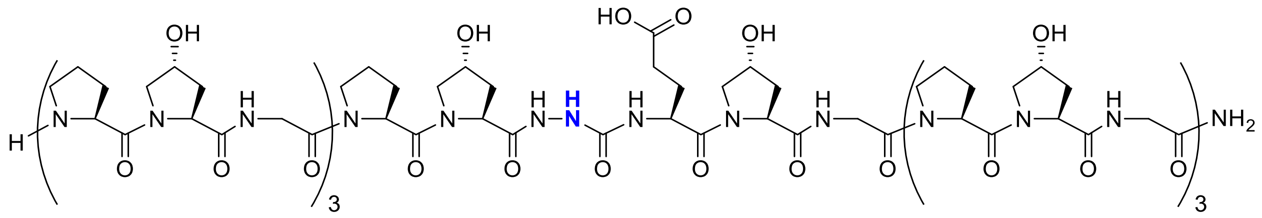

MALDI-TOF MS:

Calculated [M+Na]$^+$: 2196.964

Found: 2196.901

THERMAL DENATURATION CURVE:

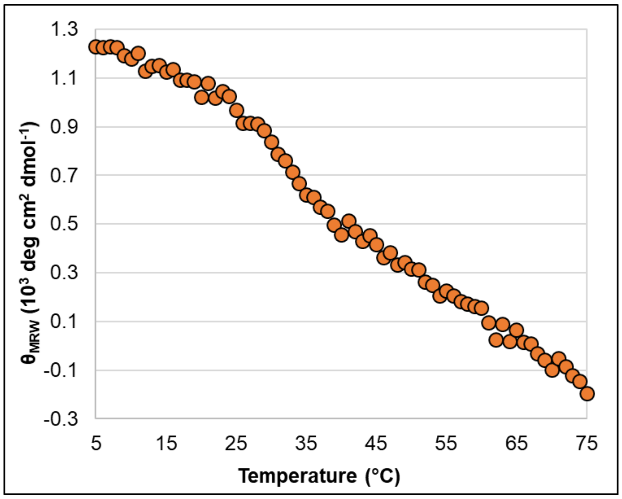

HPLC: 1-25% CH$_3$CN in 0.1% TFA H$_2$O over 30 min at 60 ˚C

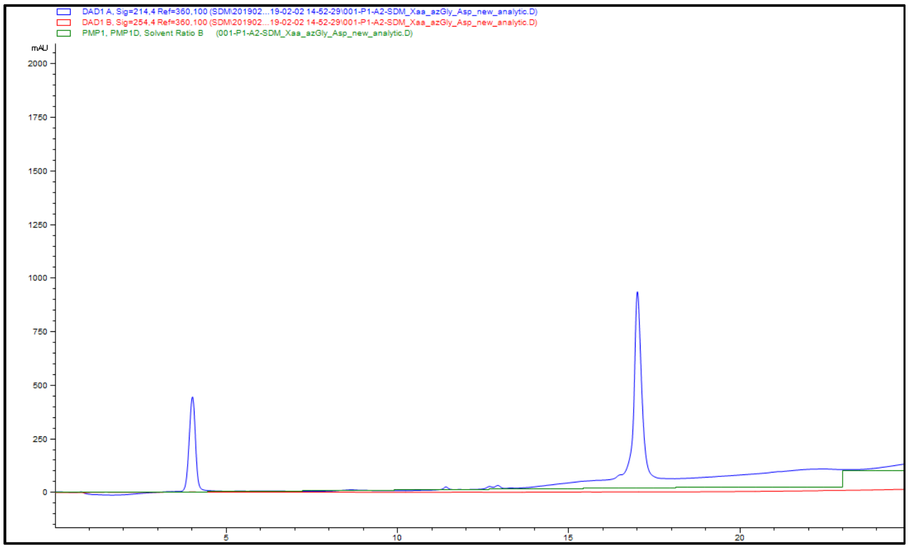

CMP **4a**: H-(Pro-Hyp-Gly)$_3$(Pro-Hyp-Gly)(**Glu**-Hyp-Gly)(Pro-Hyp-Gly)$_3$-NH$_2$

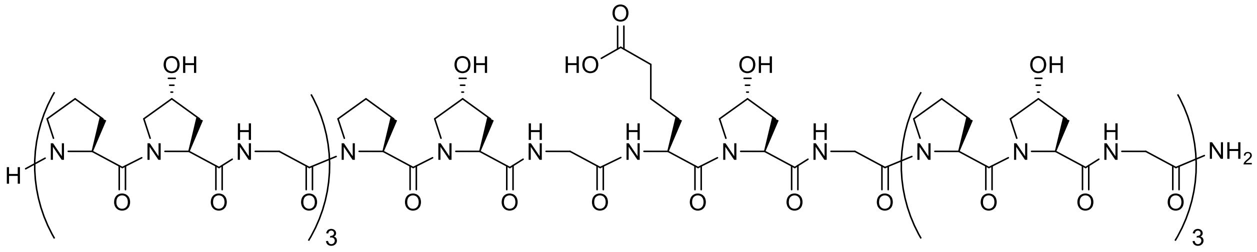

MALDI-TOF MS:

Calculated $[M+Na]^+$: 2209.945

Found: 2210.235

THERMAL DENATURATION CURVE:

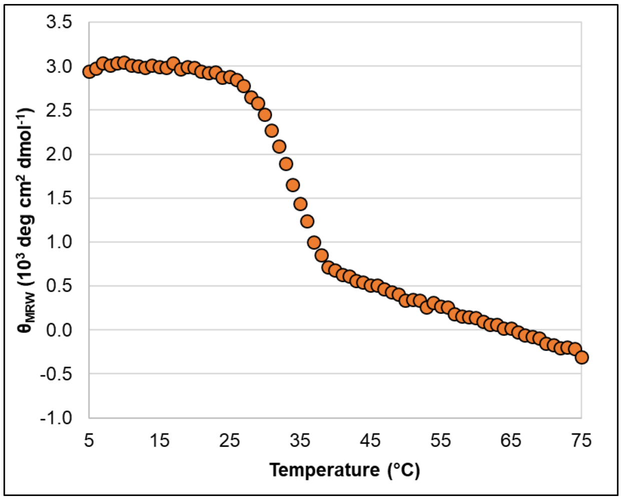

HPLC: 1-25% CH_3CN in 0.1% TFA H_2O over 30 min at 60 ˚C

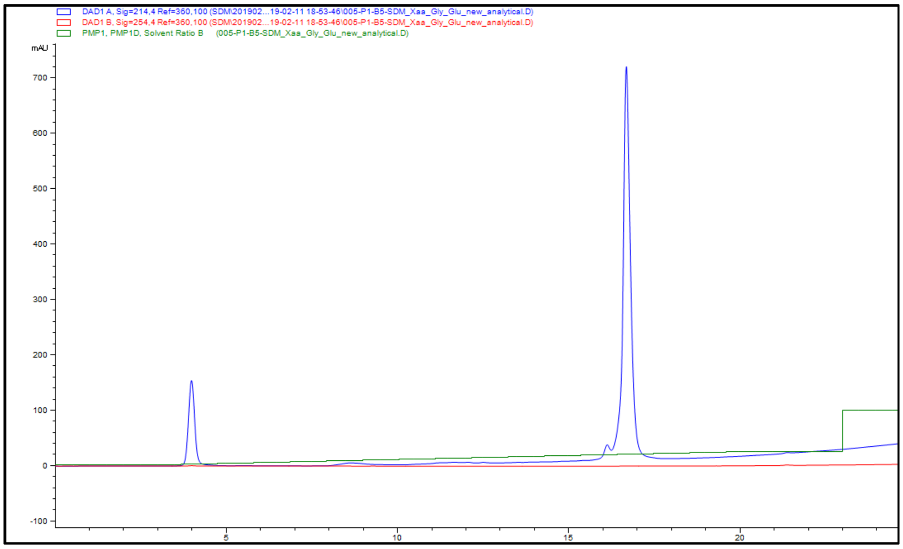

CMP **4b**: H-(Pro-Hyp-Gly)$_3$(Pro-Hyp-**azGly**)(**Glu**-Hyp-Gly)(Pro-Hyp-Gly)$_3$-NH$_2$

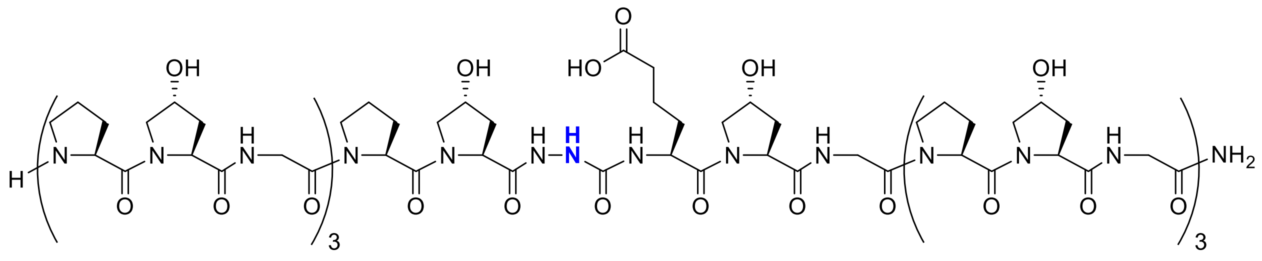

MALDI-TOF MS:

Calculated [M+Na]$^+$:	2210.940
Found:	2211.649

THERMAL DENATURATION CURVE:

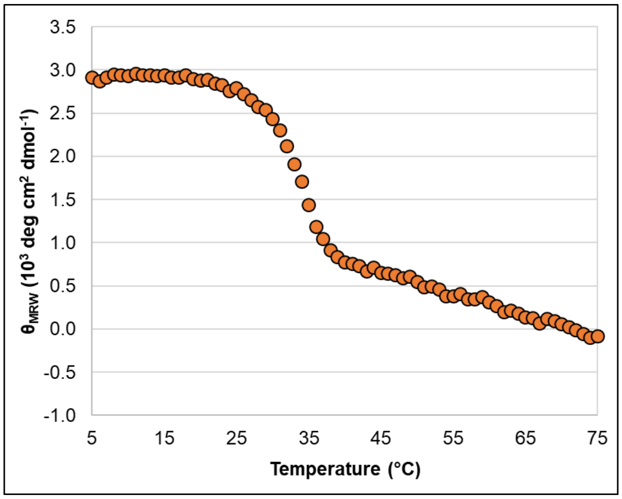

HPLC: 5-45% CH_3CN in 0.1% TFA H_2O over 30 min at 80 ˚C

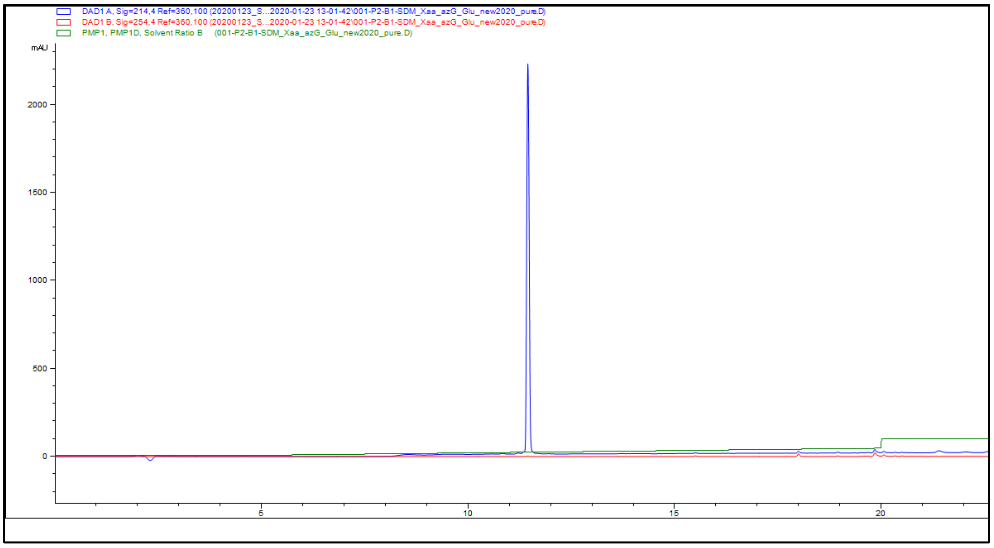

CMP **5a**: H-(Pro-Hyp-Gly)$_3$(Pro-Hyp-Gly)(**Lys**-Hyp-Gly)(Pro-Hyp-Gly)$_3$-NH$_2$

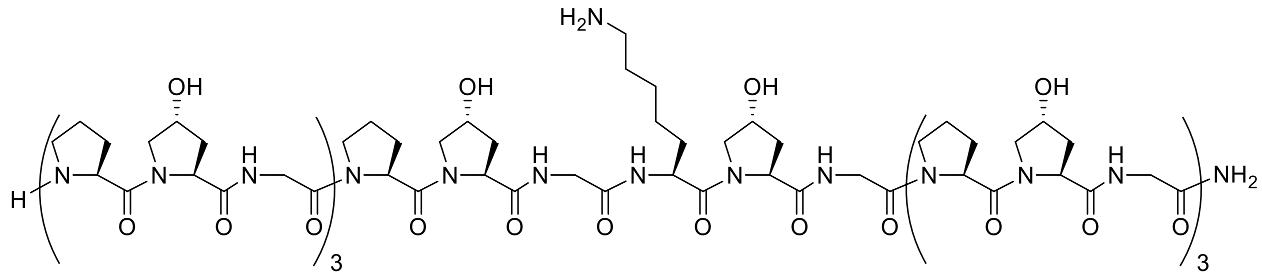

MALDI-TOF MS:

Calculated [M+Na]$^+$: 2209.037

Found: 2208.890

THERMAL DENATURATION CURVE:

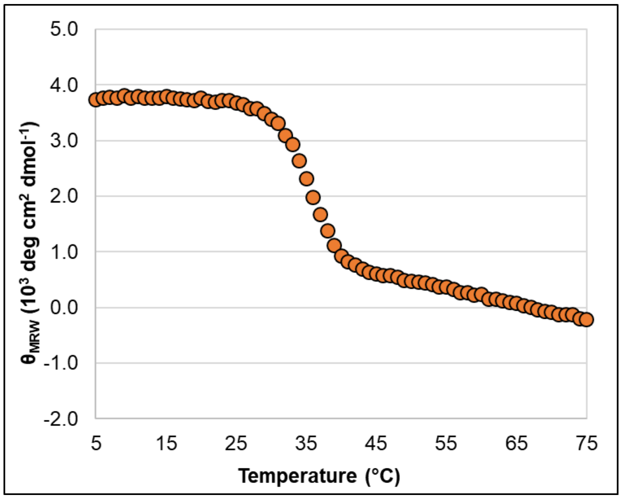

HPLC: 5-25% CH_3CN in 0.1% TFA H_2O over 30 min at 60 ˚C

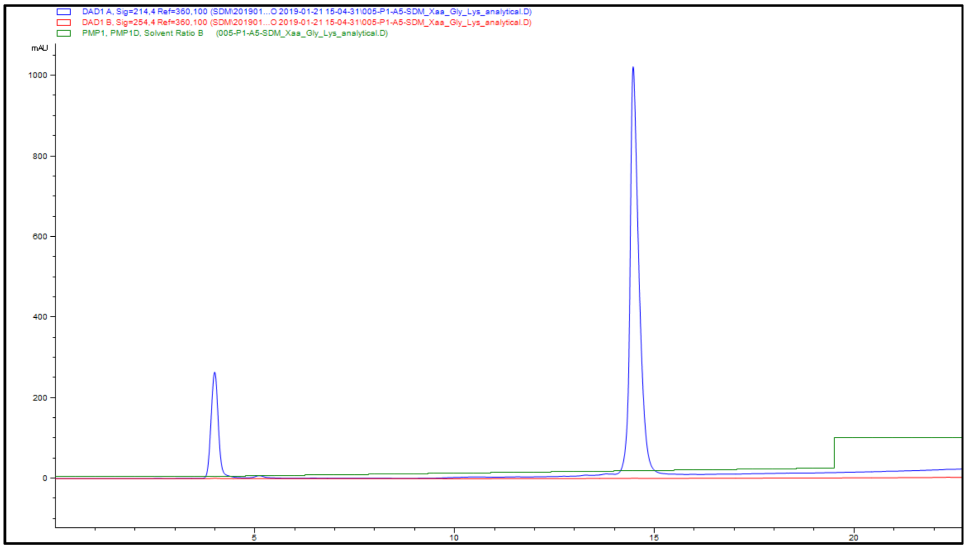

CMP **5b**: H-(Pro-Hyp-Gly)$_3$(Pro-Hyp-**azGly**)(**Lys**-Hyp-Gly)(Pro-Hyp-Gly)$_3$-NH$_2$

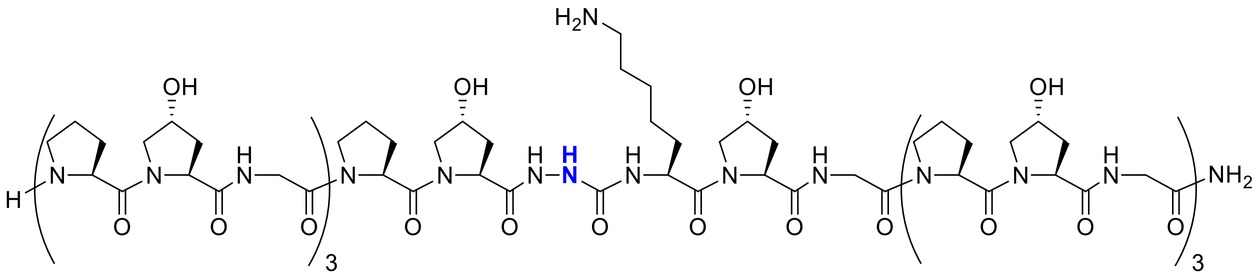

MALDI-TOF MS:

Calculated [M+Na]$^+$: 2210.032

Found: 2210.063

THERMAL DENATURATION CURVE:

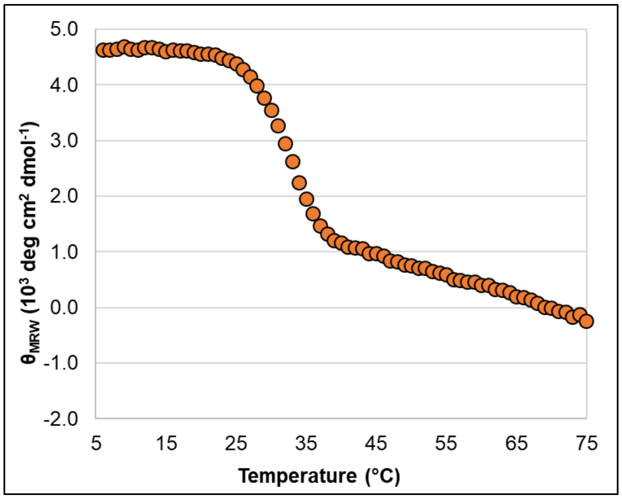

HPLC: 1-25% CH_3CN in 0.1% TFA H_2O over 30 min at 60 ˚C

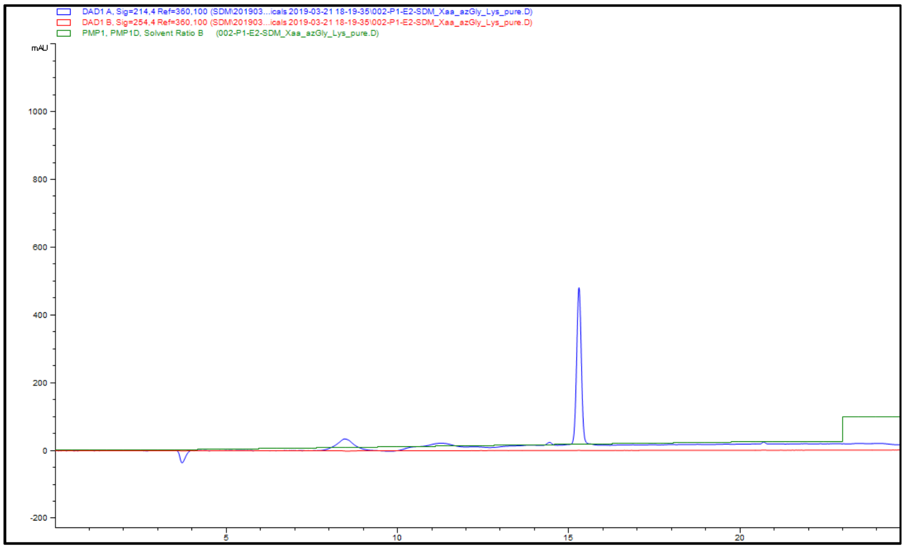

CMP **6a**: H-(Pro-Hyp-Gly)$_3$(Pro-Hyp-Gly)(**Arg**-Hyp-Gly)(Pro-Hyp-Gly)$_3$-NH$_2$

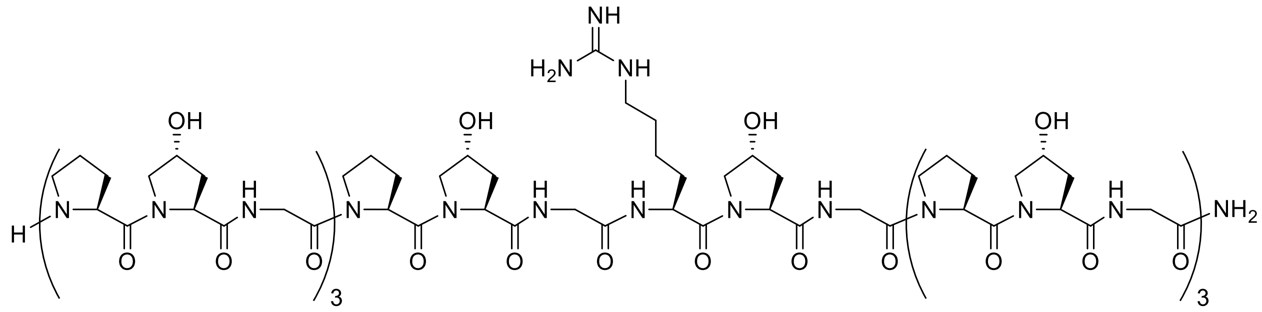

MALDI-TOF MS:

Calculated [M+Na]$^+$: 2237.043

Found: 2237.031

THERMAL DENATURATION CURVE:

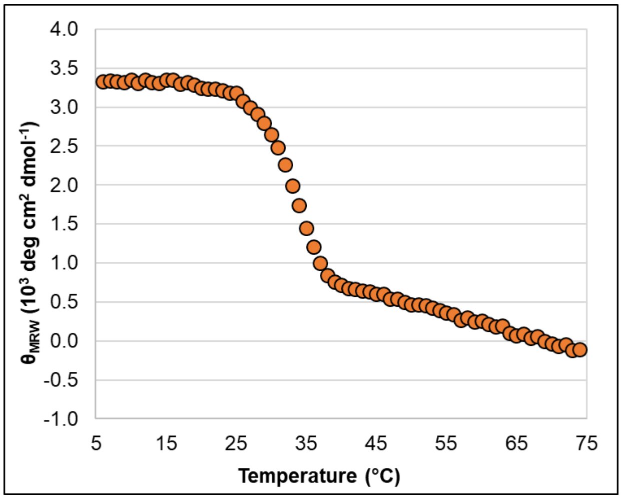

HPLC: 1-25% CH_3CN in 0.1% TFA H_2O over 30 min at 60 ˚C

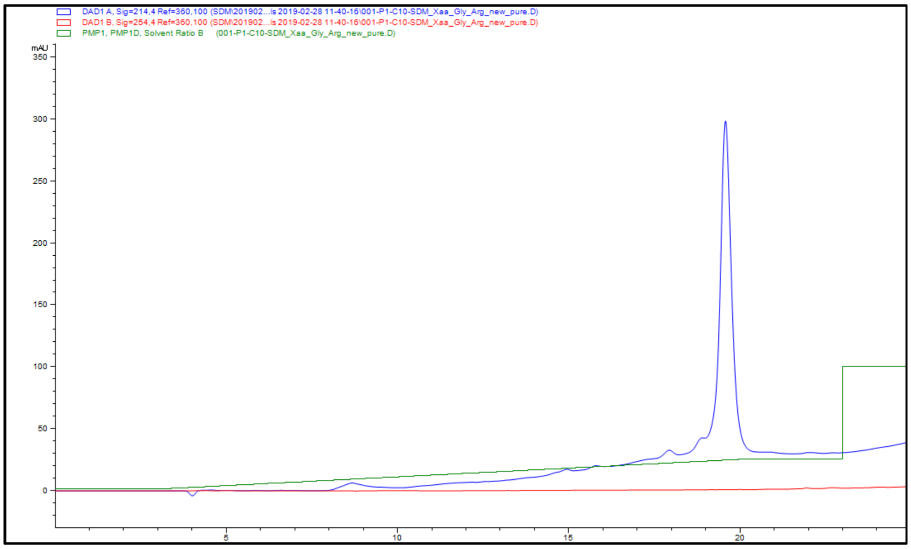

CMP **6b**: H-(Pro-Hyp-Gly)$_3$(Pro-Hyp-**azGly**)(**Arg**-Hyp-Gly)(Pro-Hyp-Gly)$_3$-NH$_2$

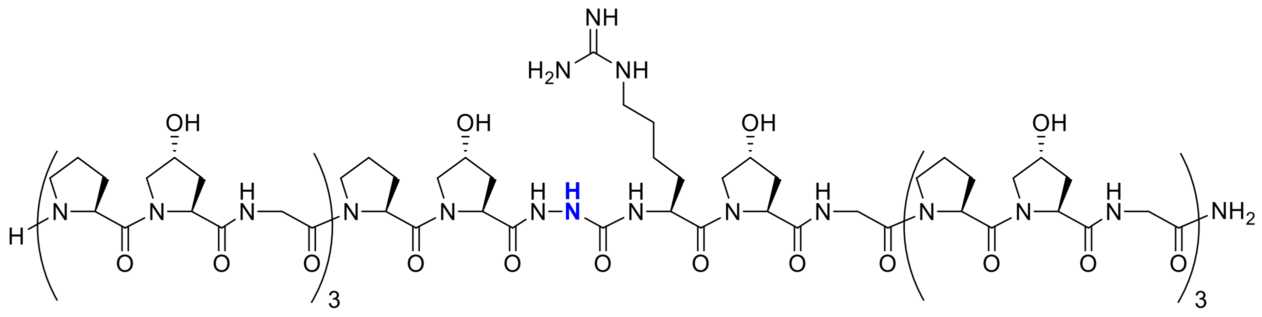

MALDI-TOF MS:

Calculated [M+Na]$^+$:	2238.038
Found:	2237.691

THERMAL DENATURATION CURVE:

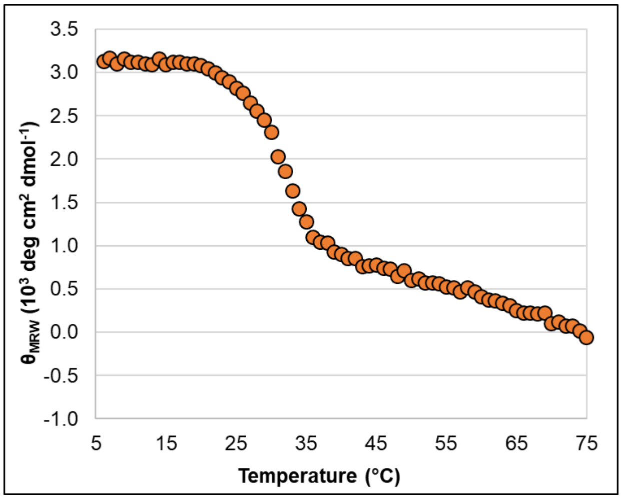

HPLC: 1-40% CH_3CN in 0.1% TFA H_2O over 30 min at 60 ˚C

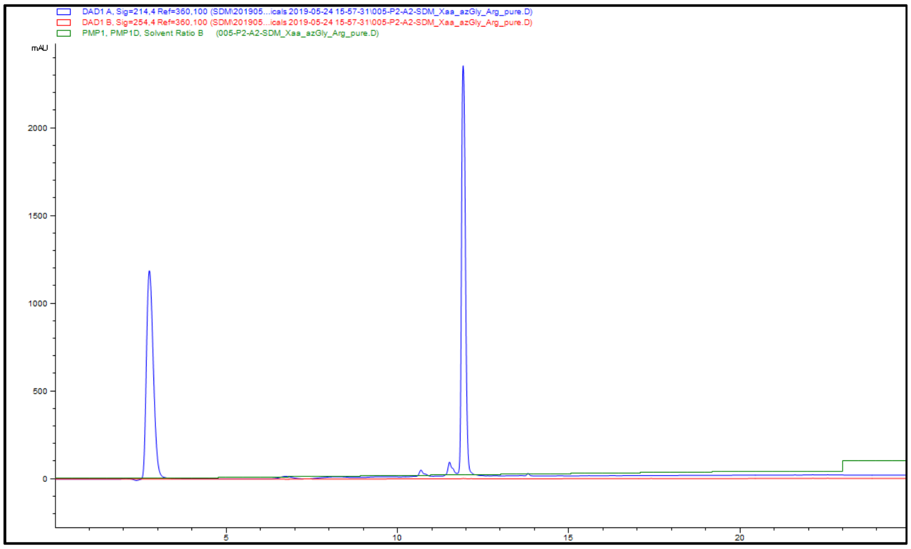

CMP **6c**: H-(Pro-Hyp-Gly)(Pro-Hyp-**azGly**)(Pro-Hyp-Gly)$_2$(**Arg**-Hyp-Gly)(Pro-Hyp-Gly)(Pro-Hyp-**azGly**)(Pro-Hyp-Gly)-NH$_2$

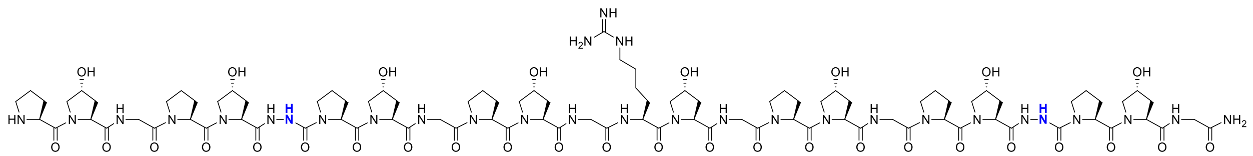

MALDI-TOF MS:

Calculated [M+Na]$^+$: 2239.034

Found: 2238.804

THERMAL DENATURATION CURVE:

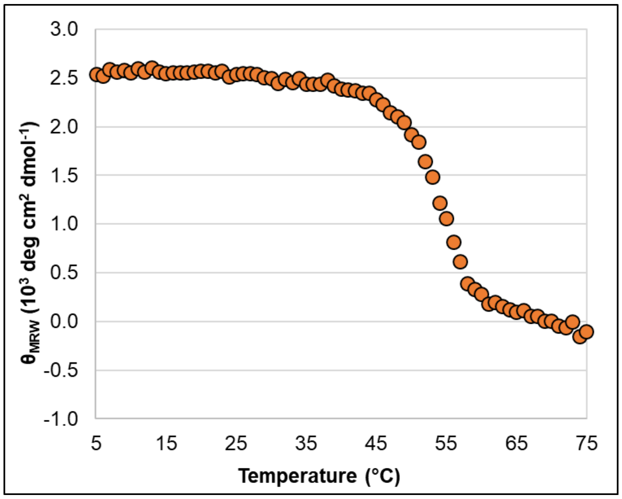

HPLC: 1-25% CH_3CN in 0.1% TFA H_2O over 30 min at 60 ˚C

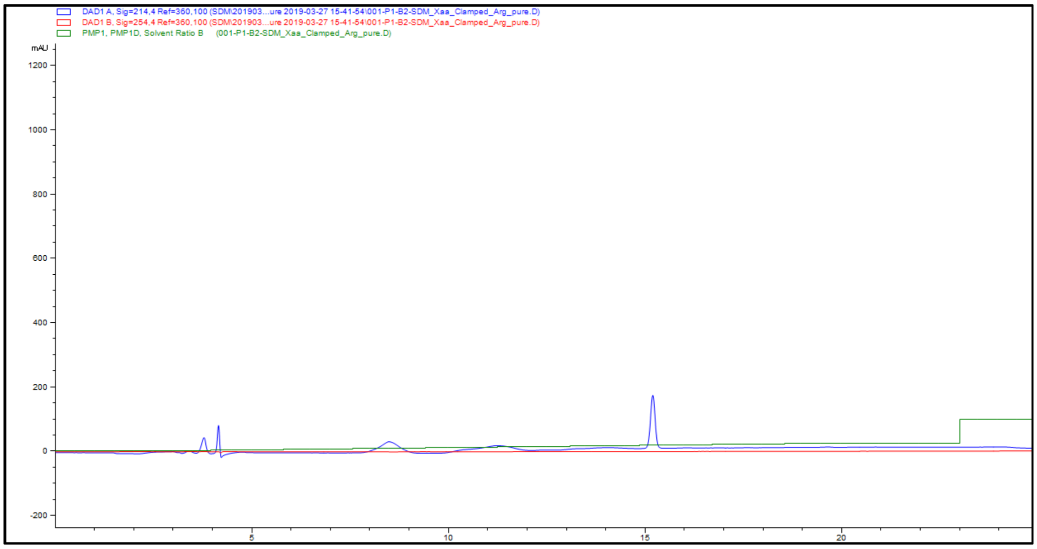

CMP **7a**: H-(Pro-Hyp-Gly)$_3$(Pro-Hyp-Gly)(Ser-Hyp-Gly)(Pro-Hyp-Gly)$_3$-NH$_2$

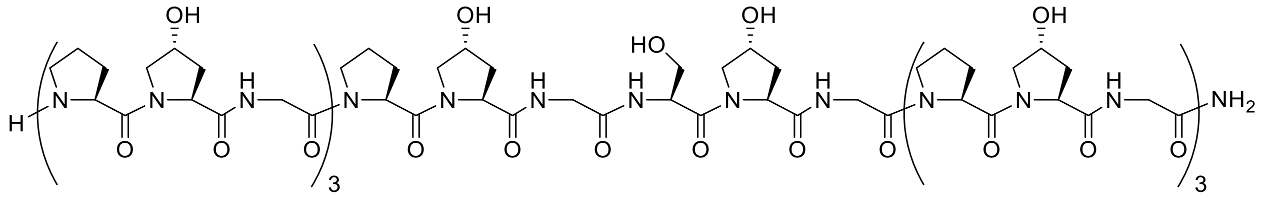

MALDI-TOF MS:

Calculated [M+Na]$^+$: 2167.974

Found: 2167.596

THERMAL DENATURATION CURVE:

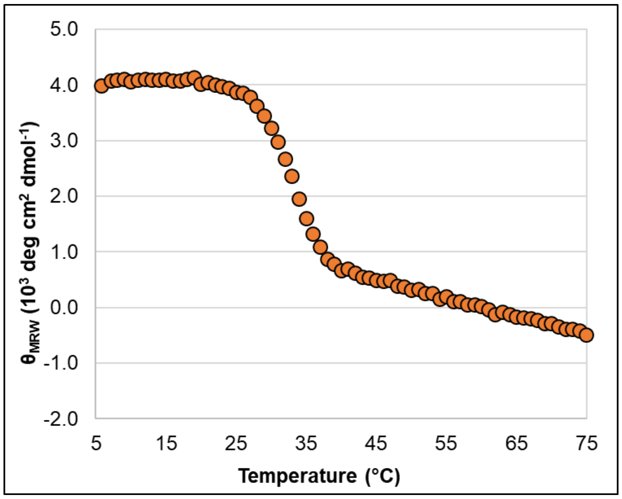

HPLC: 10-25% CH_3CN in 0.1% TFA H_2O over 30 min at 60 ˚C

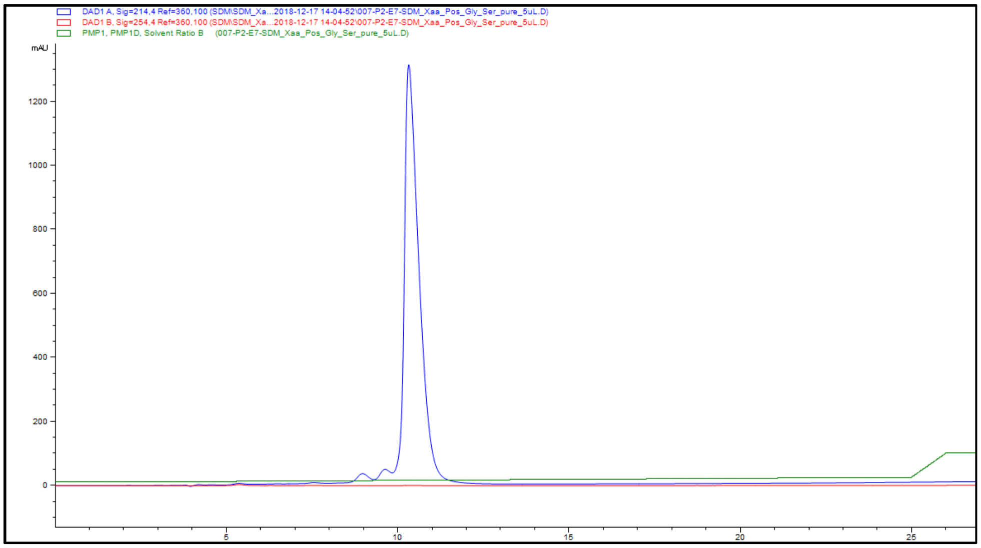

CMP **7b**: H-(Pro-Hyp-Gly)$_3$(Pro-Hyp-**azGly**)(**Ser**-Hyp-Gly)(Pro-Hyp-Gly)$_3$-NH$_2$

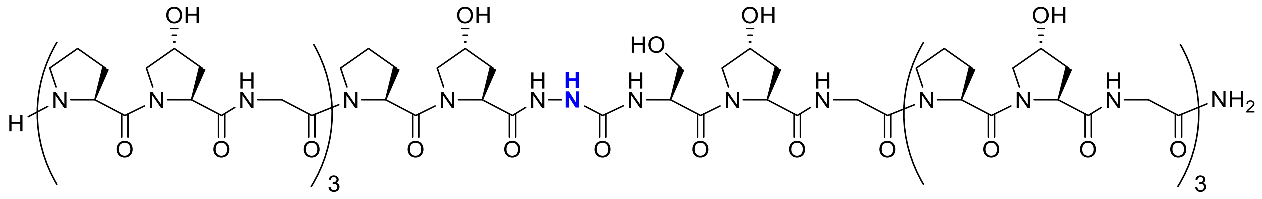

MALDI-TOF MS:

Calculated [M+Na]$^+$:	2168.969
Found:	2168.836

THERMAL DENATURATION CURVE:

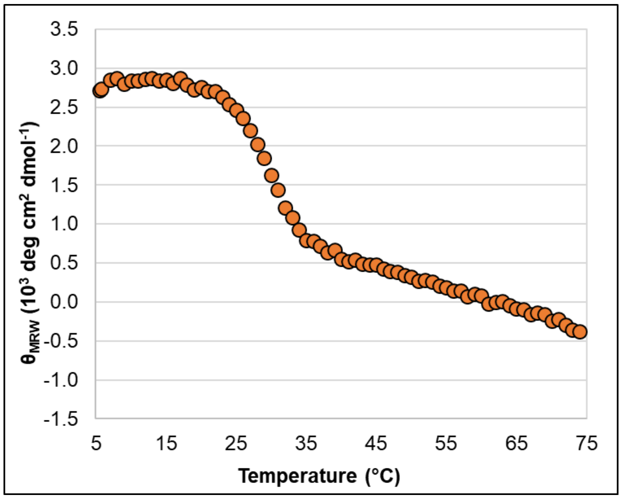

HPLC: 10-25% CH_3CN in 0.1% TFA H_2O over 30 min at 60 ˚C

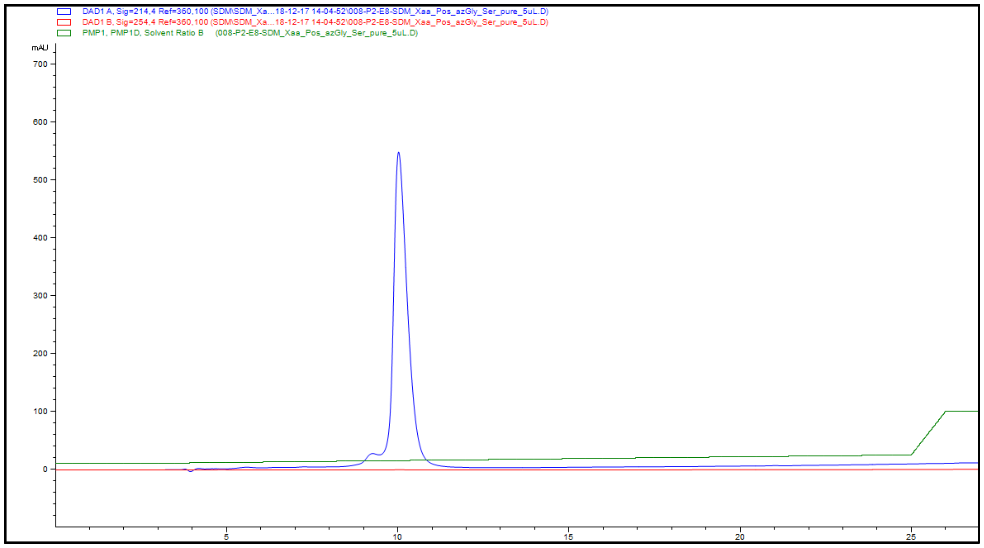

CMP **8a**: H-(Pro-Hyp-Gly)$_3$(Pro-Hyp-Gly)(**Thr**-Hyp-Gly)(Pro-Hyp-Gly)$_3$-NH$_2$

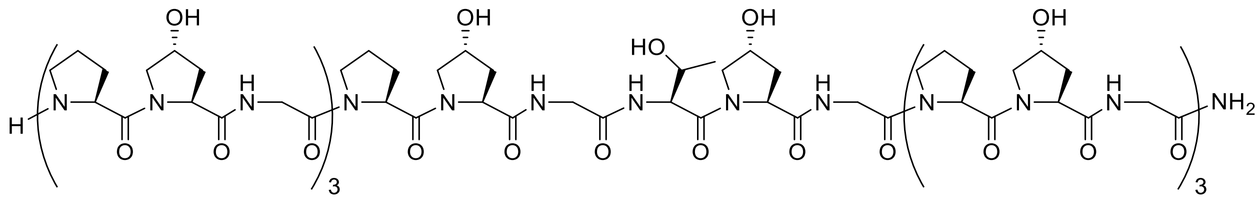

MALDI-TOF MS:

Calculated [M+Na]$^+$: 2181.990

Found: 2182.513

THERMAL DENATURATION CURVE:

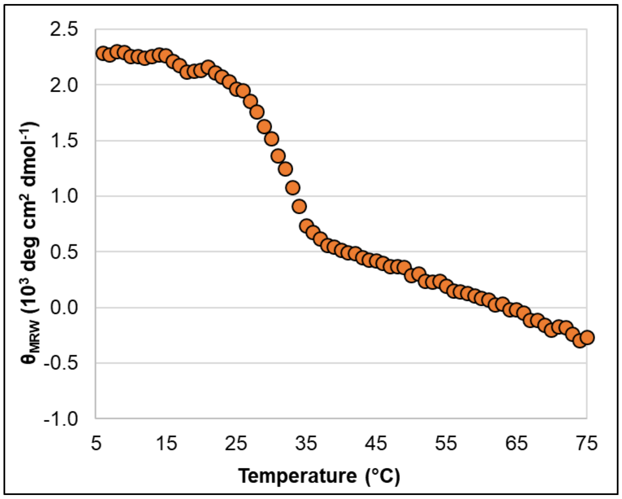

HPLC: 10-30% CH_3CN in 0.1% TFA H_2O over 30 min at 60 ˚C

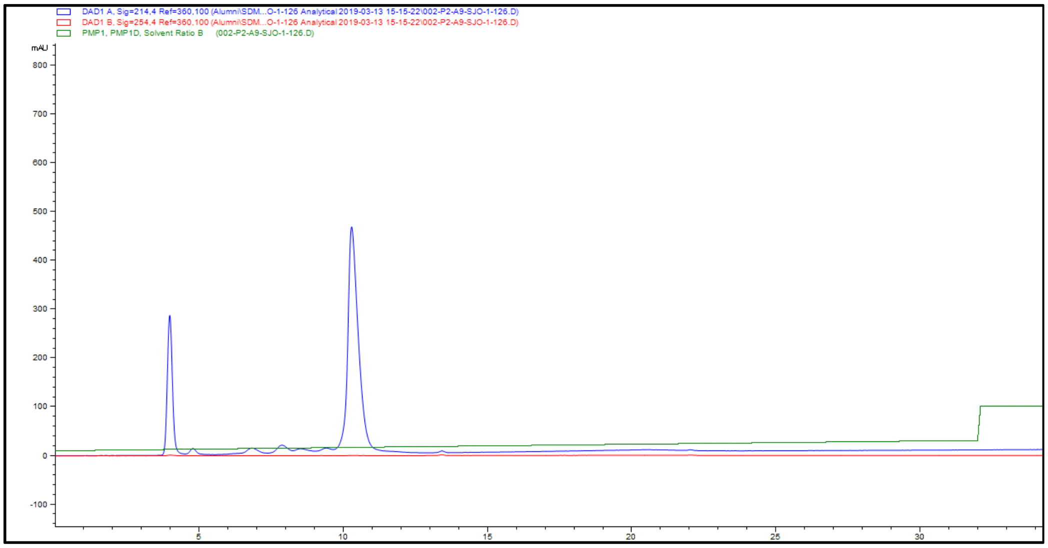

CMP **8b**: H-(Pro-Hyp-Gly)$_3$(Pro-Hyp-**azGly**)(**Thr**-Hyp-Gly)(Pro-Hyp-Gly)$_3$-NH$_2$

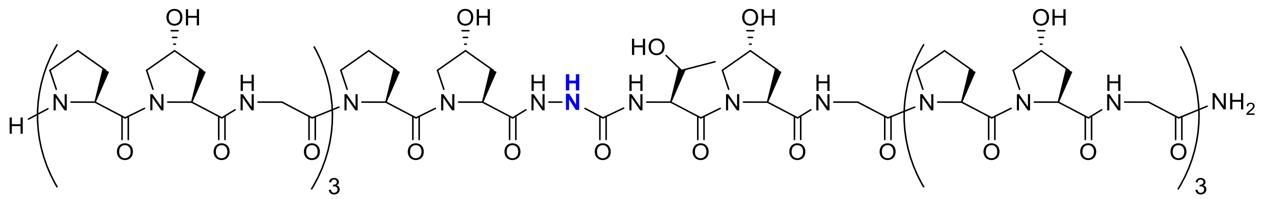

MALDI-TOF MS:

Calculated [M+Na]$^+$: 2182.985

Found: 2183.459

THERMAL DENATURATION CURVE:

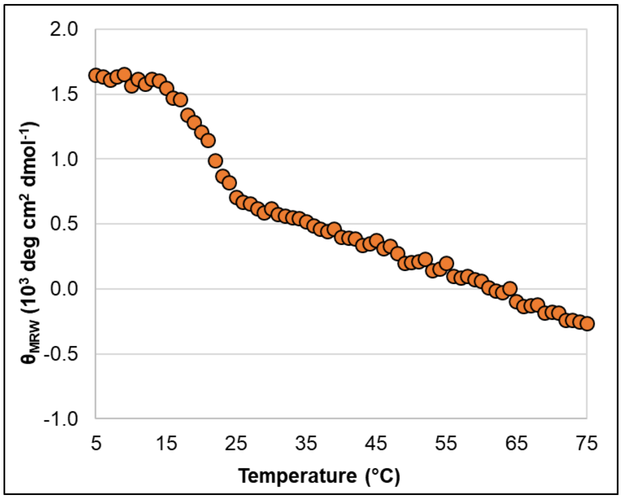

HPLC: 10-30% CH_3CN in 0.1% TFA H_2O over 30 min at 60 ˚C

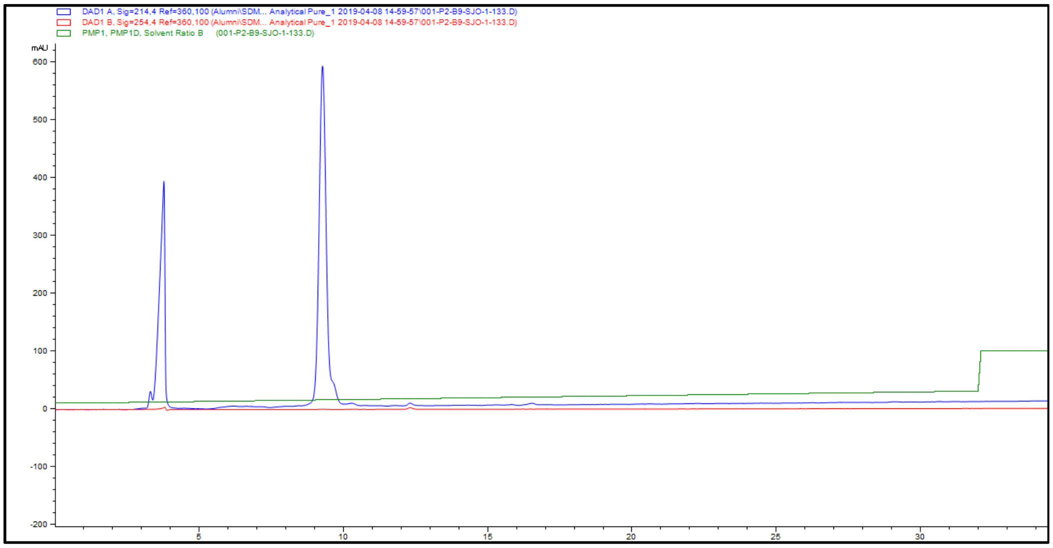

CMP **9a**: H-(Pro-Hyp-Gly)$_3$(Pro-Hyp-Gly)(**Cys**-Hyp-Gly)(Pro-Hyp-Gly)$_3$-NH$_2$

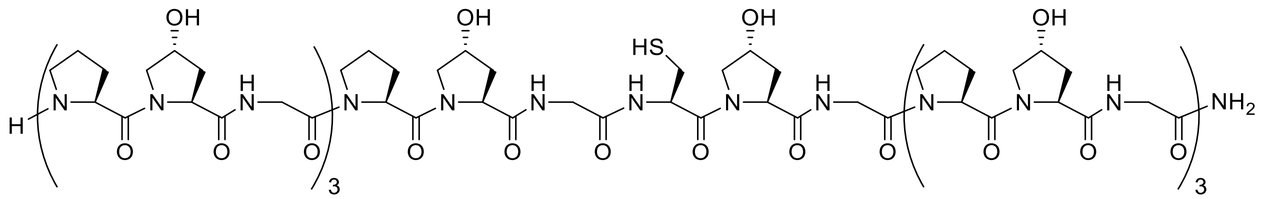

MALDI-TOF MS:

Calculated [M+Na]$^+$: 2183.952

Found: 2184.240

THERMAL DENATURATION CURVE:

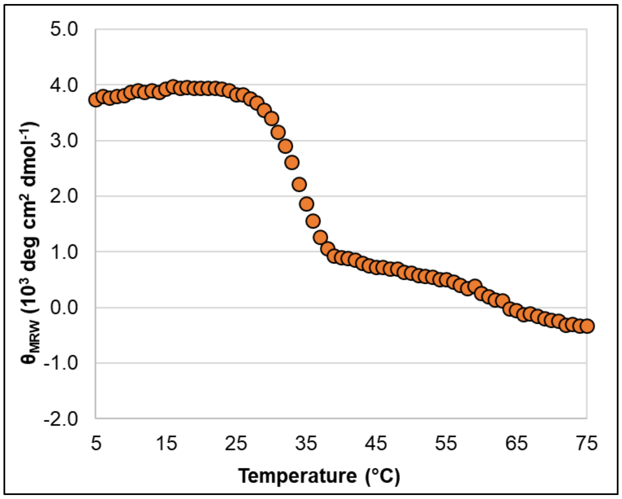

HPLC: 1-25% CH_3CN in 0.1% TFA H_2O over 30 min at 60 ˚C

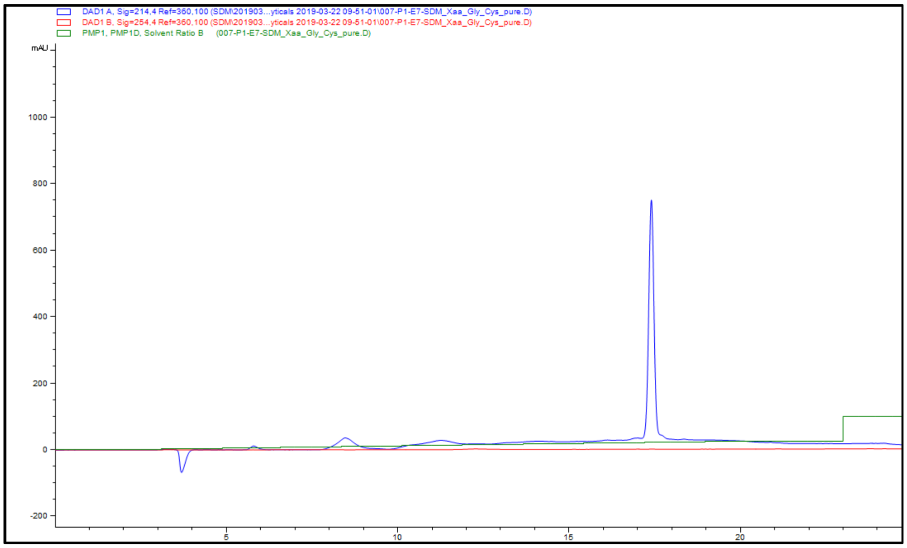

CMP **9b**: H-(Pro-Hyp-Gly)$_3$(Pro-Hyp-**azGly**)(**Cys**-Hyp-Gly)(Pro-Hyp-Gly)$_3$-NH$_2$

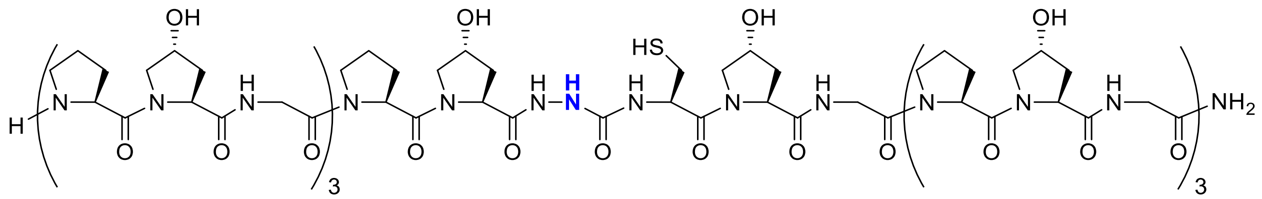

MALDI-TOF MS:

Calculated [M+Na]$^+$: 2184.947

Found: 2185.361

THERMAL DENATURATION CURVE:

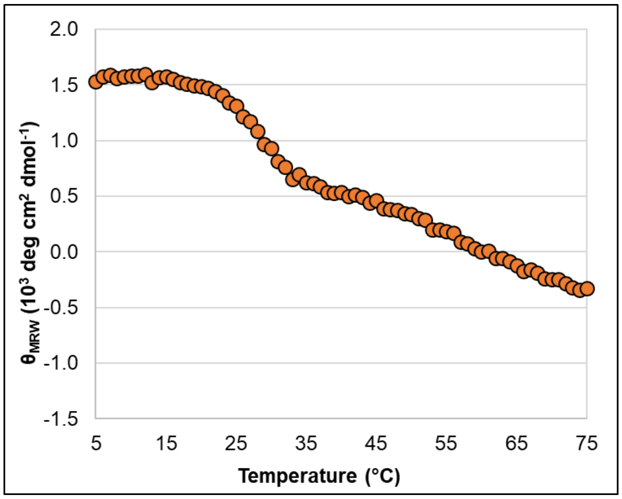

HPLC: 1-25% CH_3CN in 0.1% TFA H_2O over 30 min at 60 ˚C

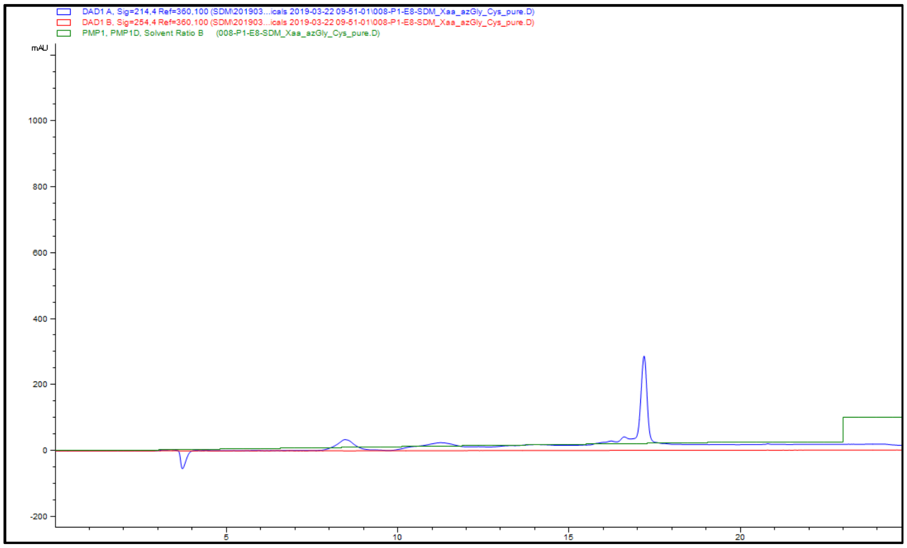

CMP **10a**: H-(Pro-Hyp-Gly)$_3$(Pro-Hyp-Gly)(Asn-Hyp-Gly)(Pro-Hyp-Gly)$_3$-NH$_2$

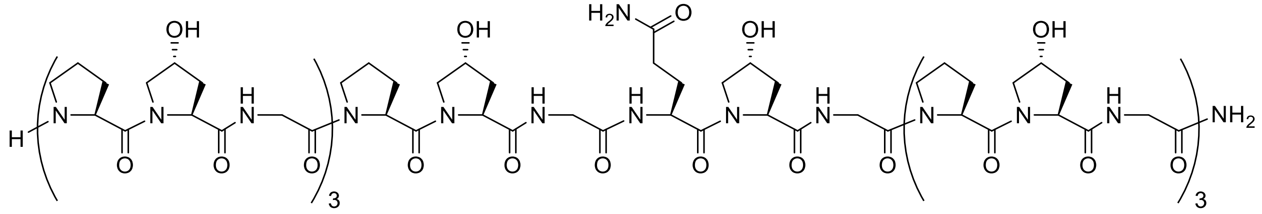

MALDI-TOF MS:

Calculated [M+Na]$^+$: 2194.985

Found: 2194.714

THERMAL DENATURATION CURVE:

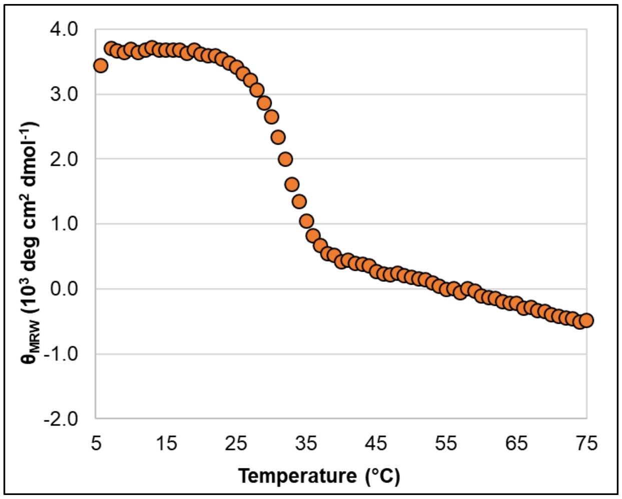

HPLC: 10-25% CH_3CN in 0.1% TFA H_2O over 30 min at 60 ˚C

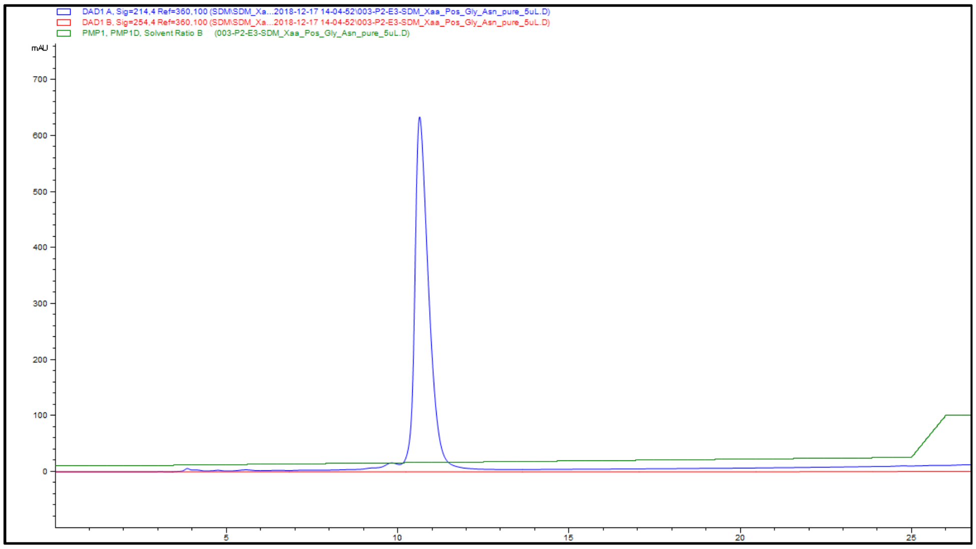

CMP **10b**: H-(Pro-Hyp-Gly)$_3$(Pro-Hyp-**azGly**)(**Asn**-Hyp-Gly)(Pro-Hyp-Gly)$_3$-NH$_2$

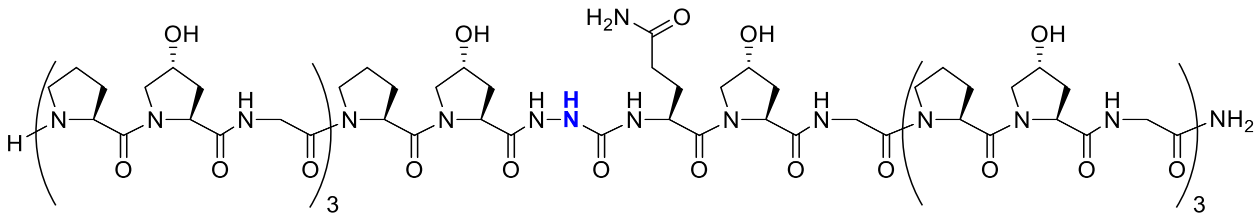

MALDI-TOF MS:

Calculated [M+Na]$^+$: 2195.980

Found: 2195.772

THERMAL DENATURATION CURVE:

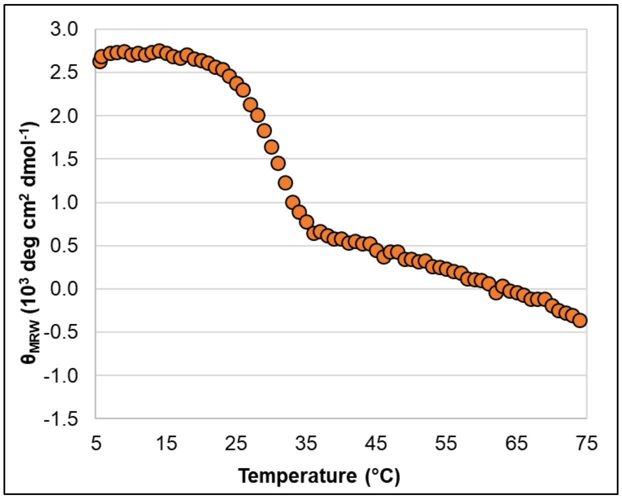

HPLC: 10-25% CH_3CN in 0.1% TFA H_2O over 30 min at 60 ˚C

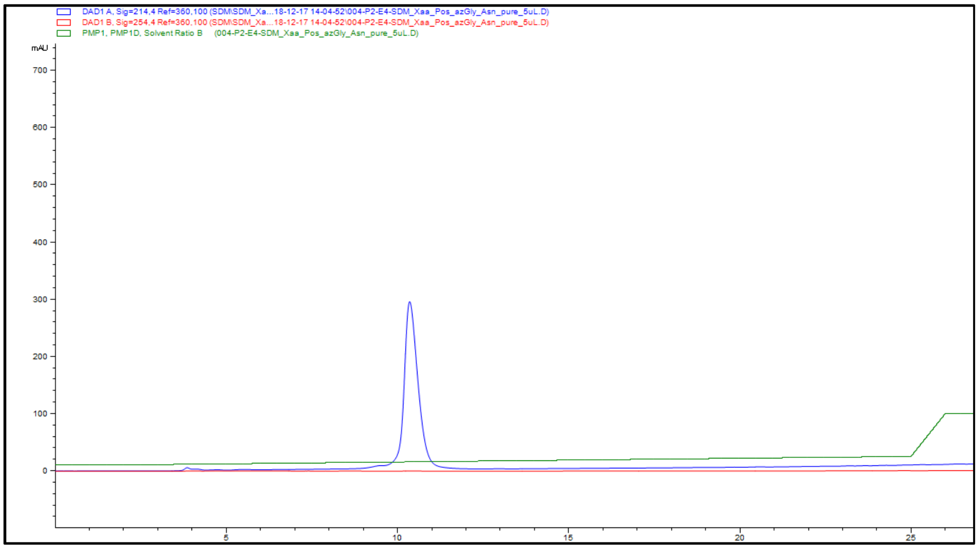

CMP **11a**: H-(Pro-Hyp-Gly)$_3$(Pro-Hyp-Gly)(**Gln**-Hyp-Gly)(Pro-Hyp-Gly)$_3$-NH$_2$

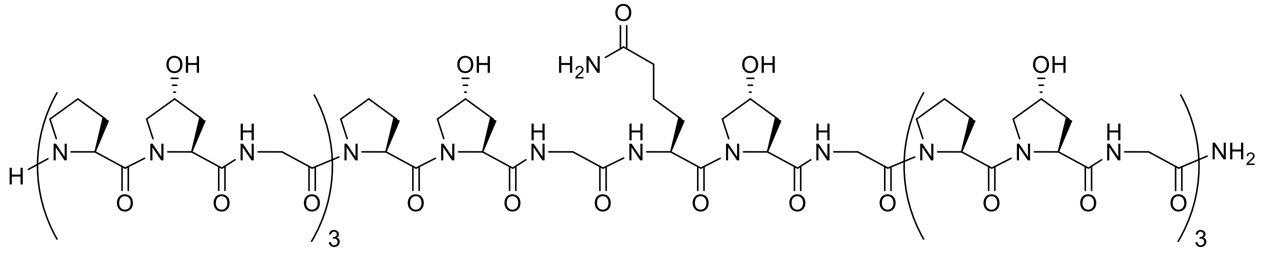

MALDI-TOF MS:

Calculated [M+Na]$^+$:	2209.001
Found:	2208.731

THERMAL DENATURATION CURVE:

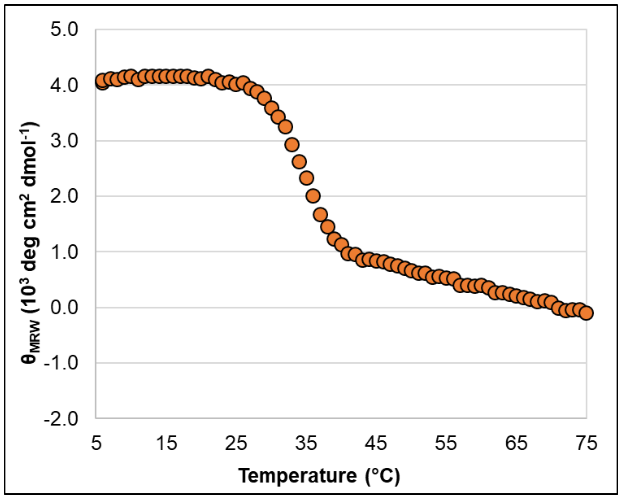

HPLC: 10-25% CH_3CN in 0.1% TFA H_2O over 30 min at 60 ˚C

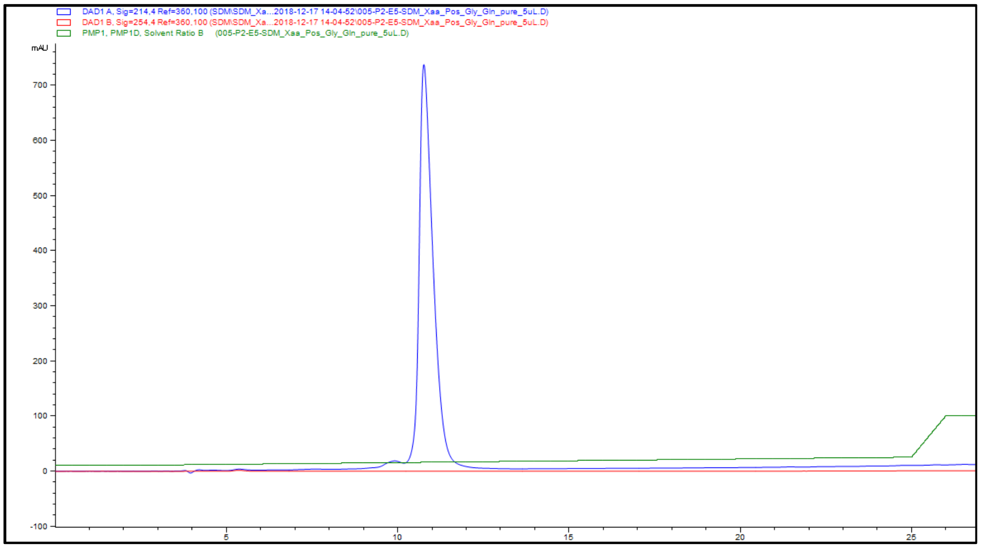

CMP **11b**: H-(Pro-Hyp-Gly)$_3$(Pro-Hyp-**azGly**)(**Gln**-Hyp-Gly)(Pro-Hyp-Gly)$_3$-NH$_2$

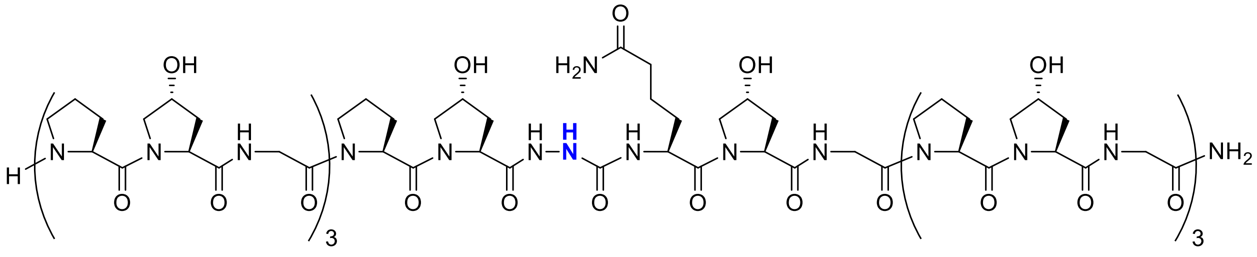

MALDI-TOF MS:

Calculated [M+Na]$^+$: 2209.996

Found: 2209.719

THERMAL DENATURATION CURVE:

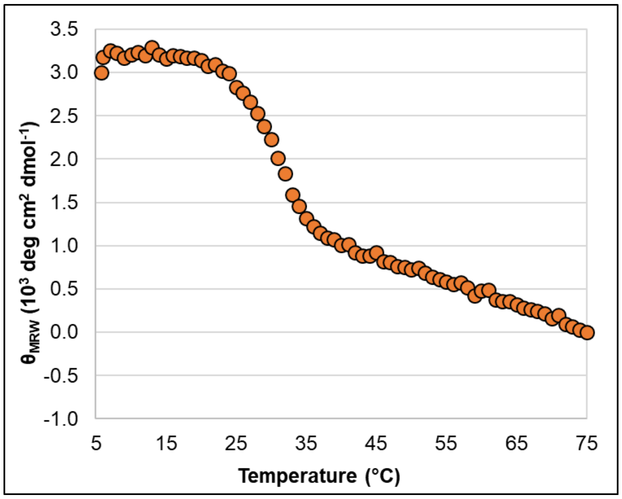

HPLC: 10-25% CH_3CN in 0.1% TFA H_2O over 30 min at 60 ˚C

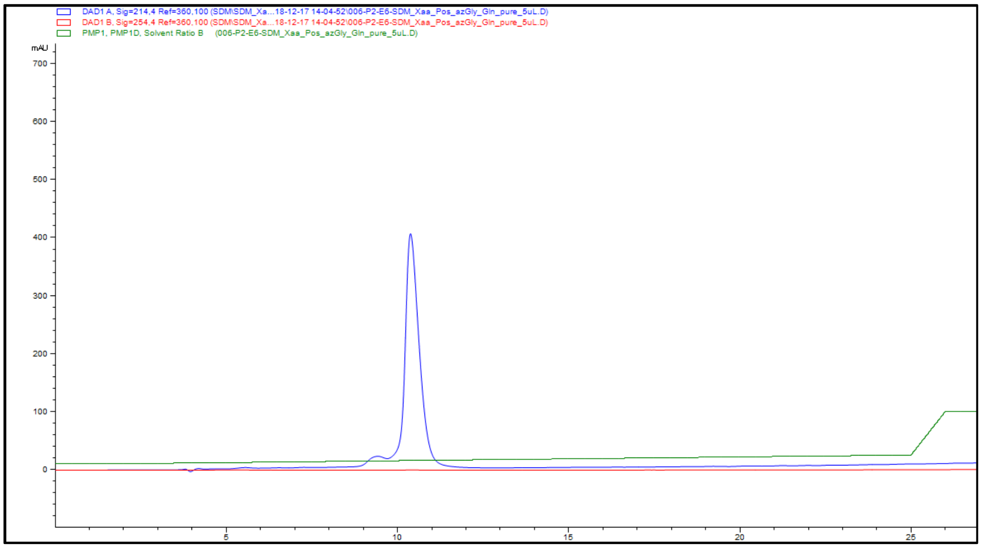

CMP **12a**: H-(Pro-Hyp-Gly)$_3$(Pro-Hyp-Gly)(**Gly**-Hyp-Gly)(Pro-Hyp-Gly)$_3$-NH_2

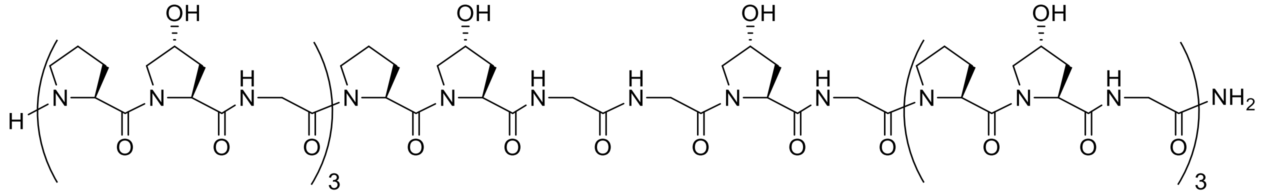

MALDI-TOF MS:

Calculated $[M+Na]^+$:	2137.956
Found:	2138.001

THERMAL DENATURATION CURVE:

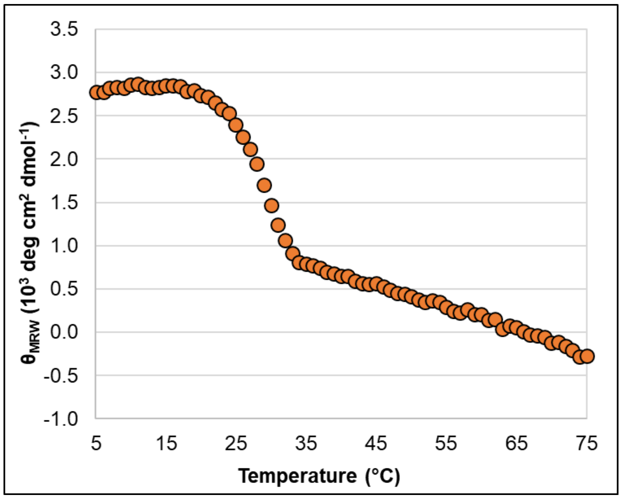

HPLC: 1-25% CH_3CN in 0.1% TFA H_2O over 30 min at 60 ˚C

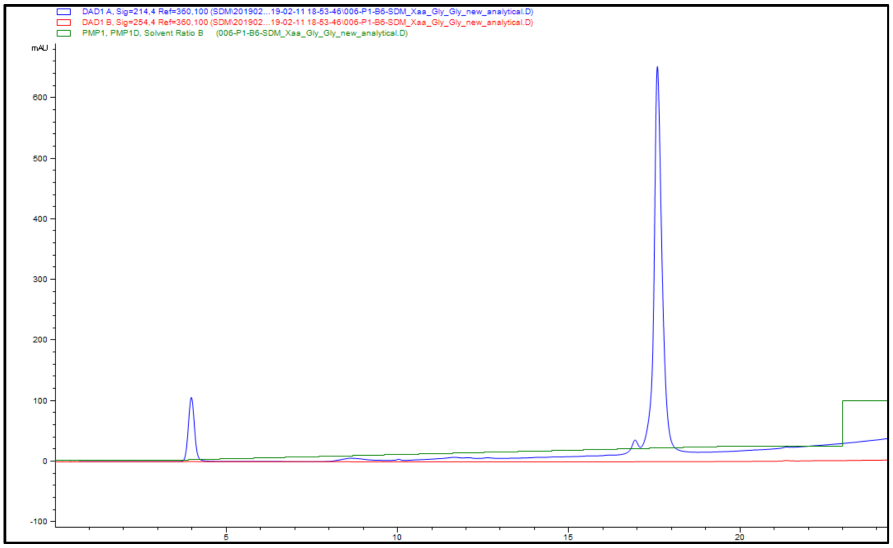

CMP **12b**: H-(Pro-Hyp-Gly)$_3$(Pro-Hyp-**azGly**)(**Gly**-Hyp-Gly)(Pro-Hyp-Gly)$_3$-NH$_2$

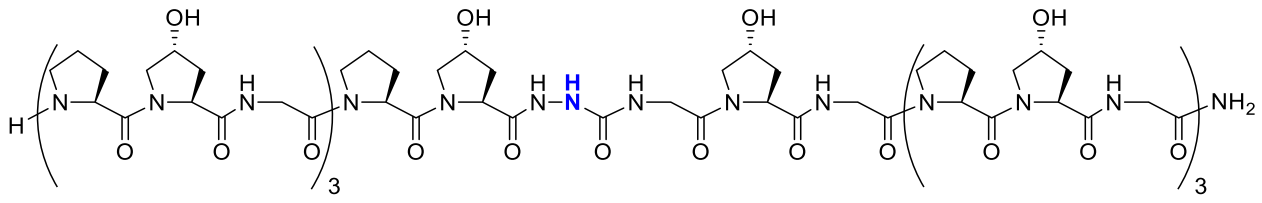

MALDI-TOF MS:

Calculated [M+Na]$^+$: 2138.951

Found: 2138.978

THERMAL DENATURATION CURVE:

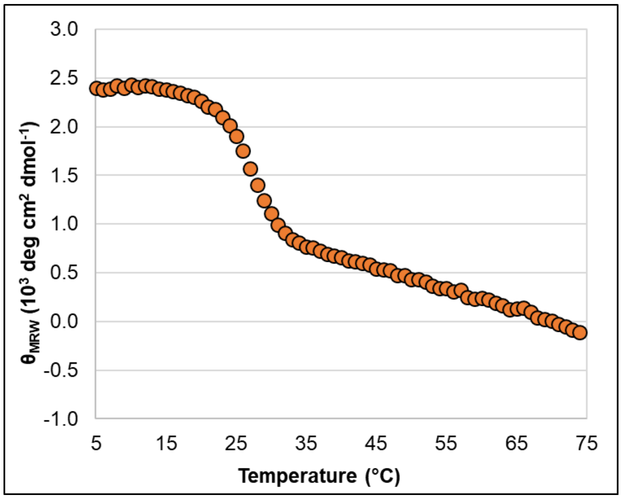

HPLC: 1-25% CH_3CN in 0.1% TFA H_2O over 30 min at 60 ˚C

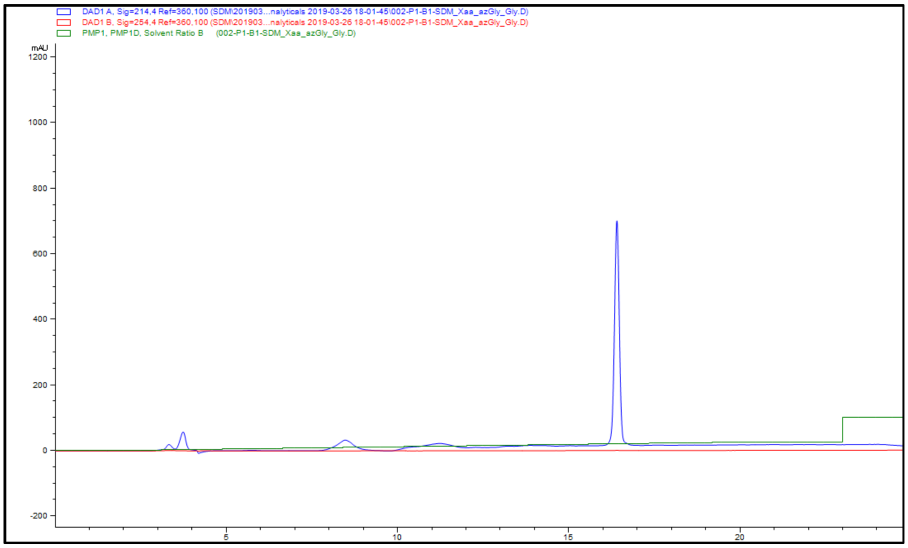

CMP **13a**: H-(Pro-Hyp-Gly)$_3$(Pro-Hyp-Gly)(**Ala**-Hyp-Gly)(Pro-Hyp-Gly)$_3$-NH$_2$

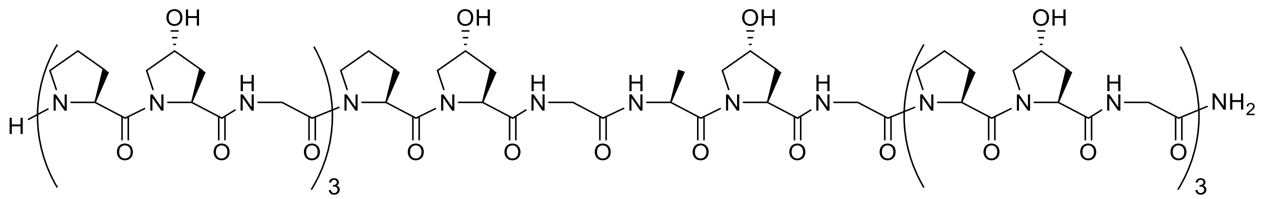

MALDI-TOF MS:

Calculated $[M+Na]^+$: 2151.980

Found: 2151.915

THERMAL DENATURATION CURVE:

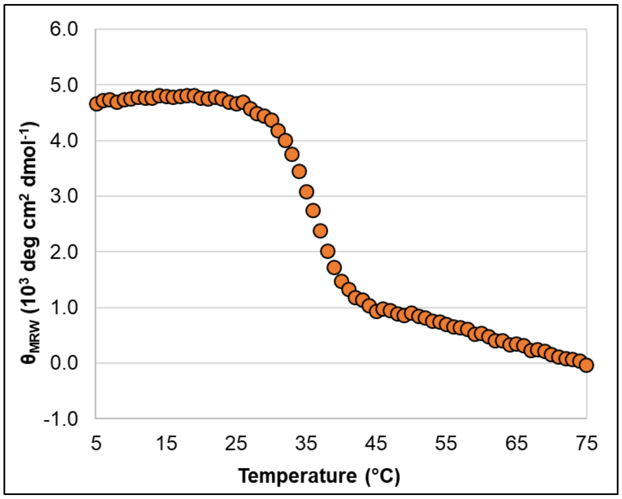

HPLC: 1-25% CH_3CN in 0.1% TFA H_2O over 30 min at 60 ˚C

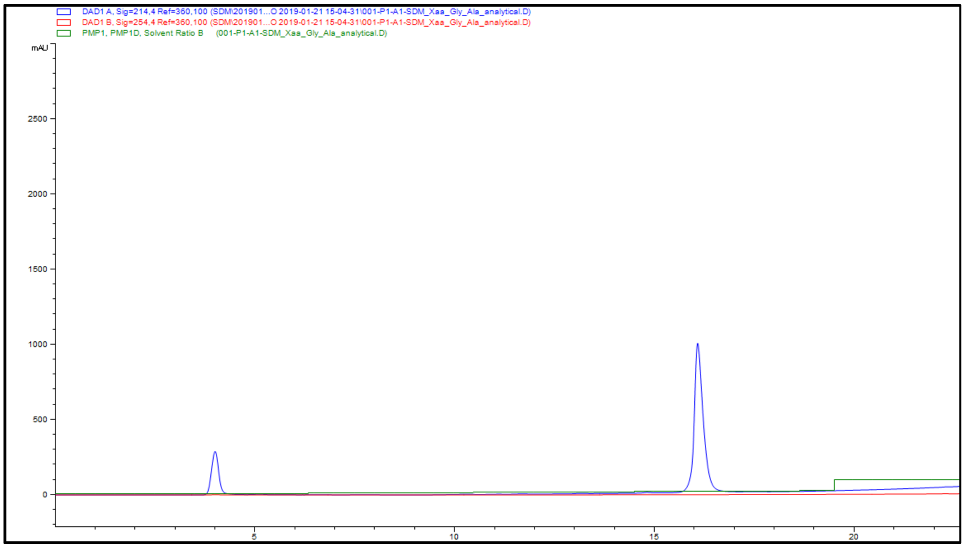

CMP **13b**: H-(Pro-Hyp-Gly)$_3$(Pro-Hyp-**azGly**)(**Ala**-Hyp-Gly)(Pro-Hyp-Gly)$_3$-NH$_2$

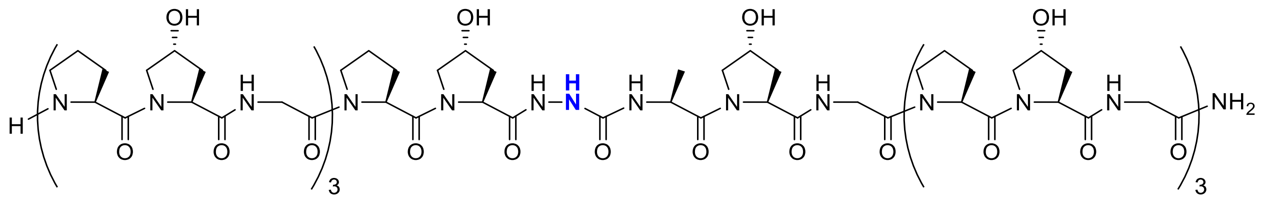

MALDI-TOF MS:

Calculated [M+Na]$^+$: 2152.975

Found: 2153.061

THERMAL DENATURATION CURVE:

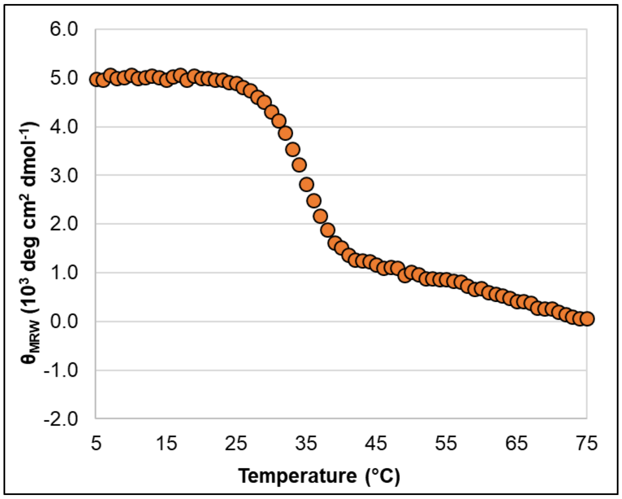

HPLC: 1-25% CH_3CN in 0.1% TFA H_2O over 30 min at 60 ˚C

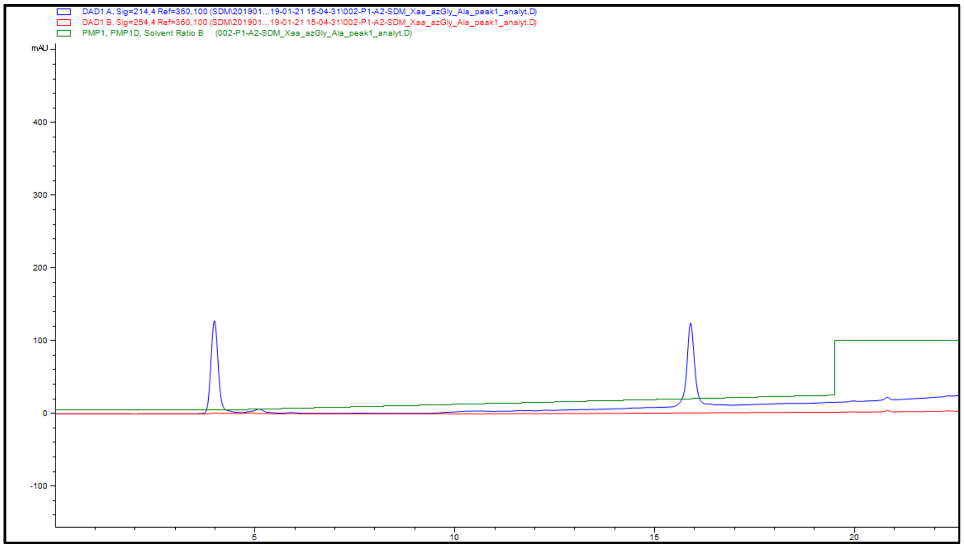

CMP **14a**: H-(Pro-Hyp-Gly)$_3$(Pro-Hyp-Gly)(**Val**-Hyp-Gly)(Pro-Hyp-Gly)$_3$-NH$_2$

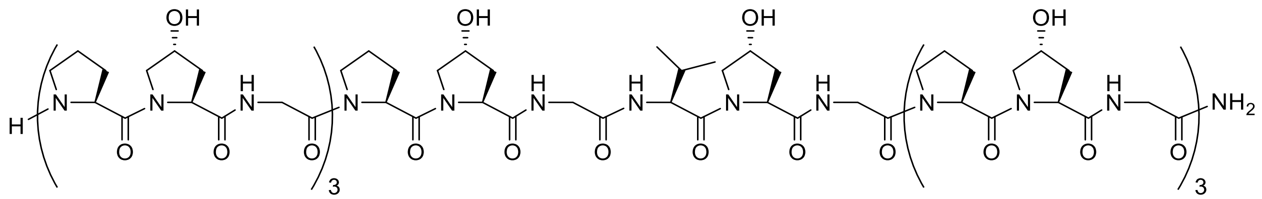

MALDI-TOF MS:

Calculated [M+Na]$^+$: 2180.011

Found: 2179.871

THERMAL DENATURATION CURVE:

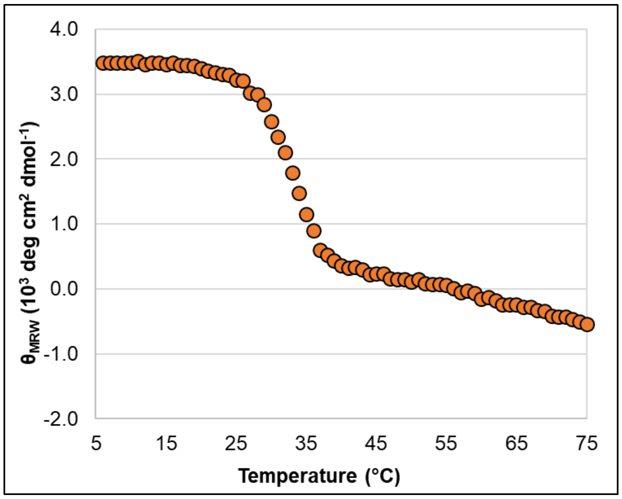

HPLC: 1-25% CH_3CN in 0.1% TFA H_2O over 30 min at 60 ˚C

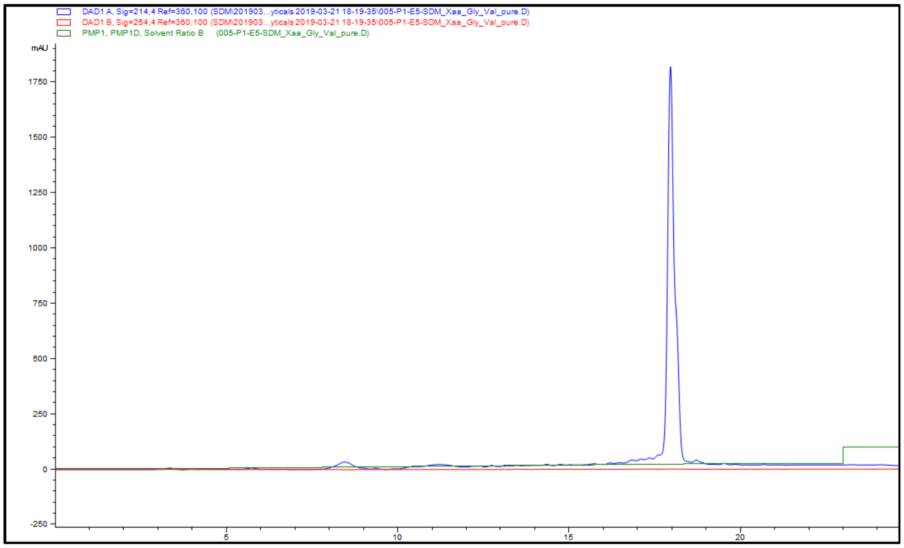

CMP **14b**: H-(Pro-Hyp-Gly)$_3$(Pro-Hyp-**azGly**)(**Val**-Hyp-Gly)(Pro-Hyp-Gly)$_3$-NH$_2$

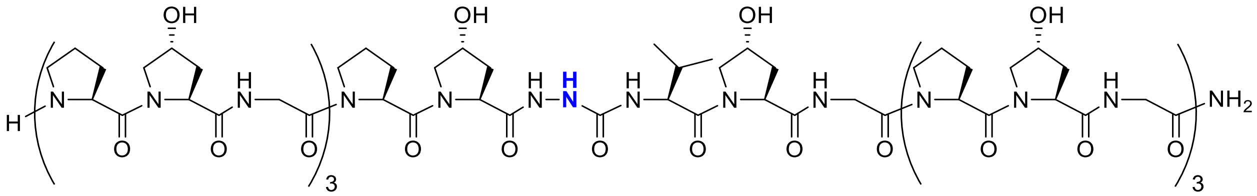

MALDI-TOF MS:

Calculated [M+Na]$^+$:	2181.006
Found:	2180.956

THERMAL DENATURATION CURVE:

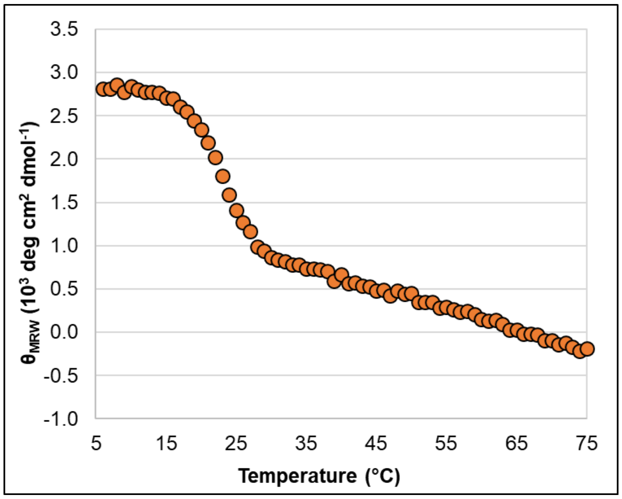

HPLC: 1-25% CH_3CN in 0.1% TFA H_2O over 30 min at 60 ˚C

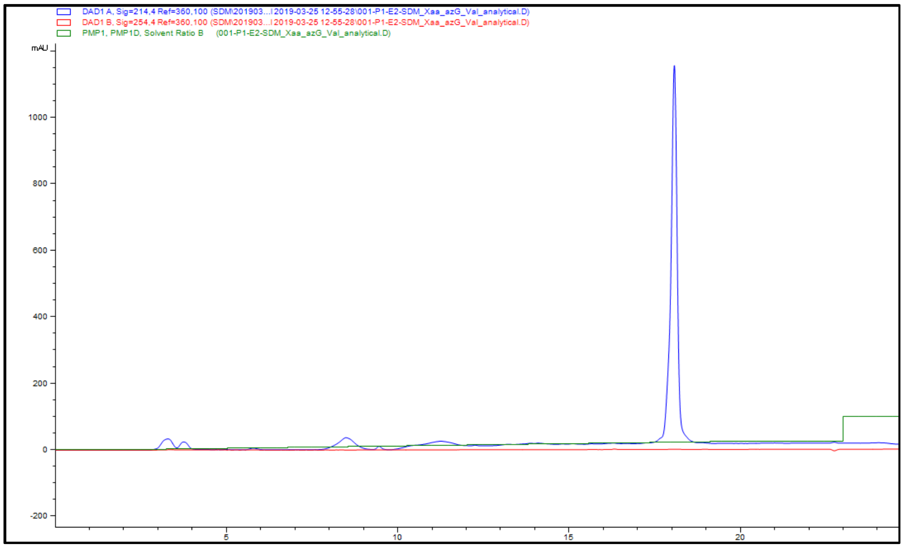

CMP **14c**: H-(Pro-Hyp-Gly)(Pro-Hyp-**azGly**)(Pro-Hyp-Gly)$_2$(**Val**-Hyp-Gly)(Pro-Hyp-Gly)(Pro-Hyp-**azGly**)(Pro-Hyp-Gly)-NH_2

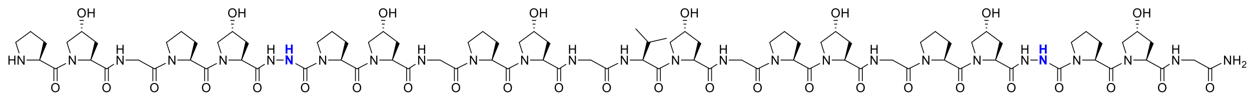

MALDI-TOF MS:

Calculated $[M+Na]^+$: 2182.001

Found: 2181.542

THERMAL DENATURATION CURVE:

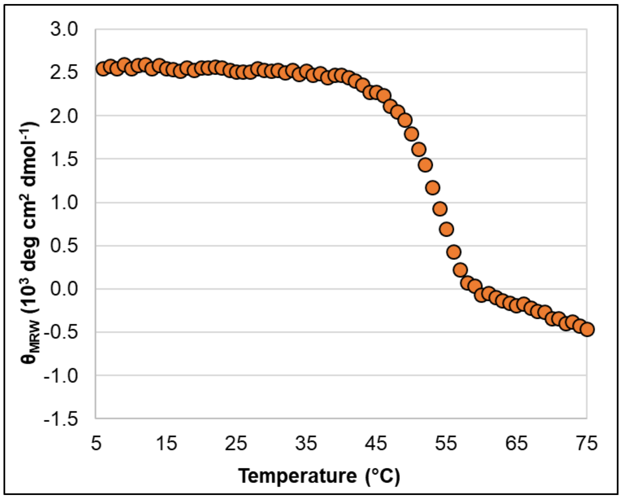

HPLC: 1-25% CH_3CN in 0.1% TFA H_2O over 30 min at 60 ˚C

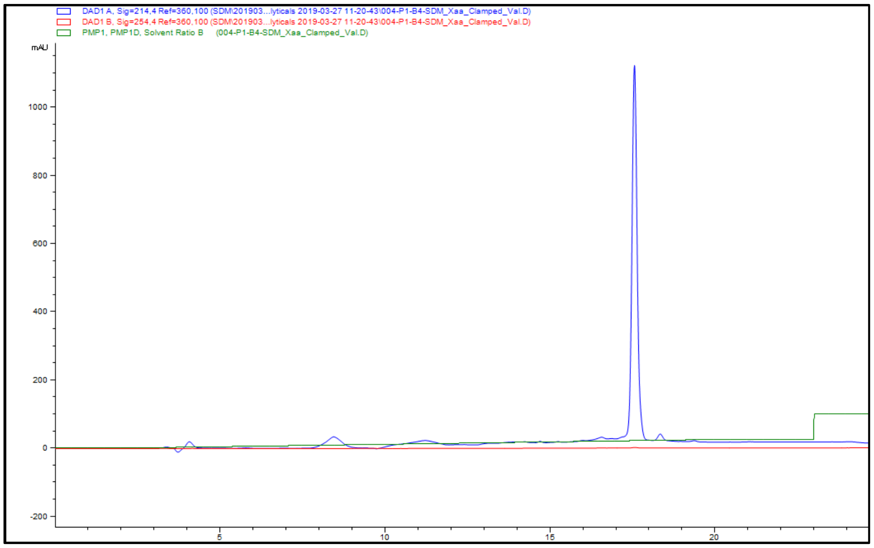

CMP **15a**: H-(Pro-Hyp-Gly)$_3$(Pro-Hyp-Gly)(**Leu**-Hyp-Gly)(Pro-Hyp-Gly)$_3$-NH$_2$

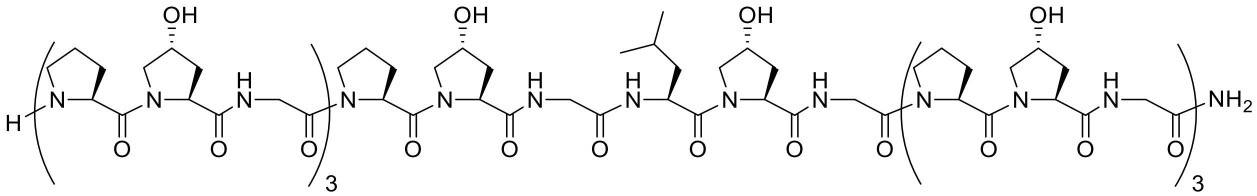

MALDI-TOF MS:

Calculated [M+Na]$^+$: 2194.026

Found: 2193.711

THERMAL DENATURATION CURVE:

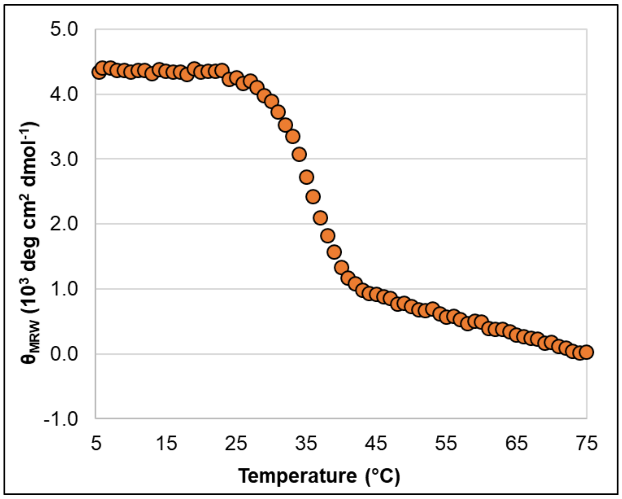

HPLC: 10-25% CH_3CN in 0.1% TFA H_2O over 30 min at 60 ˚C

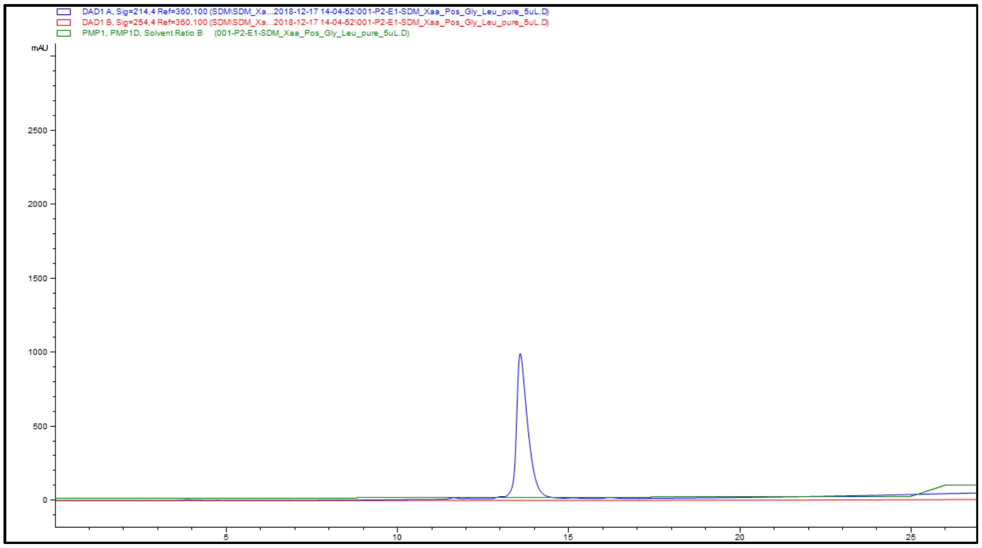

CMP **15b**: H-(Pro-Hyp-Gly)$_3$(Pro-Hyp-**azGly**)(**Leu**-Hyp-Gly)(Pro-Hyp-Gly)$_3$-NH_2

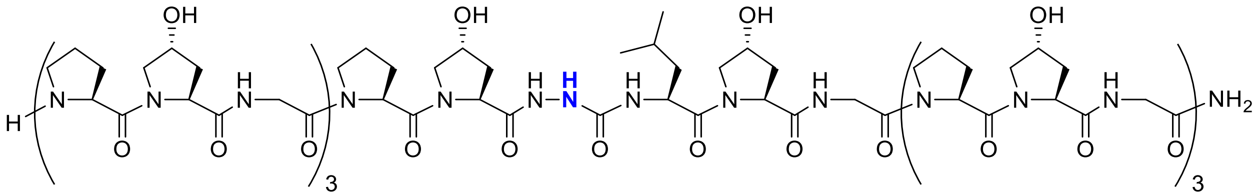

MALDI-TOF MS:

Calculated $[M+Na]^+$: 2195.021

Found: 2194.882

THERMAL DENATURATION CURVE:

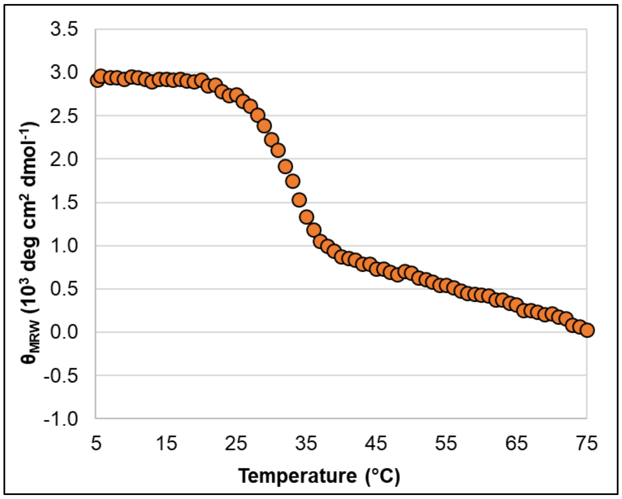

HPLC: 10-25% CH_3CN in 0.1% TFA H_2O over 30 min at 60 ˚C

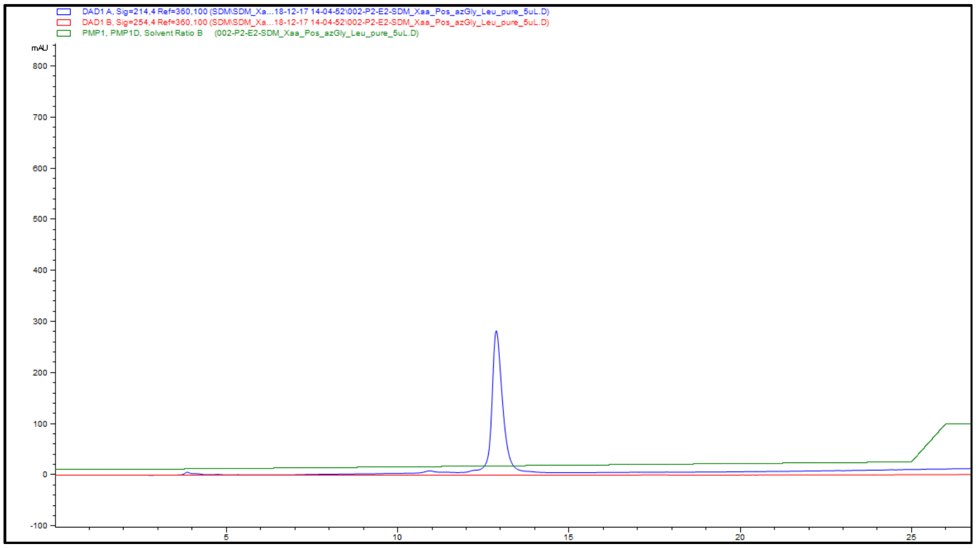

CMP **16a**: H-(Pro-Hyp-Gly)$_3$(Pro-Hyp-Gly)(Ile-Hyp-Gly)(Pro-Hyp-Gly)$_3$-NH$_2$

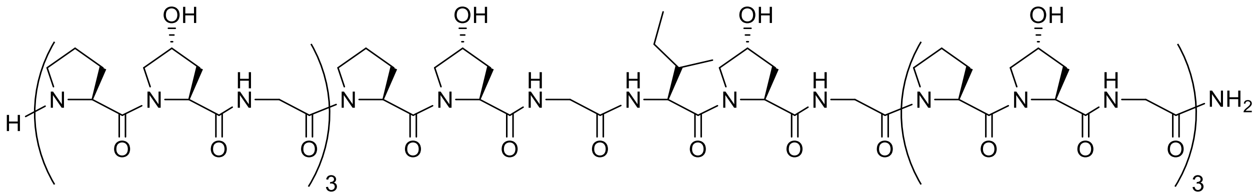

MALDI-TOF MS:

Calculated [M+Na]$^+$: 2194.026

Found: 2194.076

THERMAL DENATURATION CURVE:

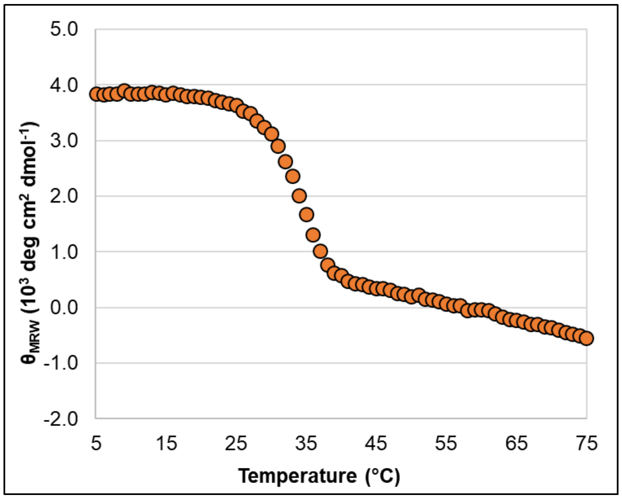

HPLC: 1-25% CH_3CN in 0.1% TFA H_2O over 30 min at 60 ˚C

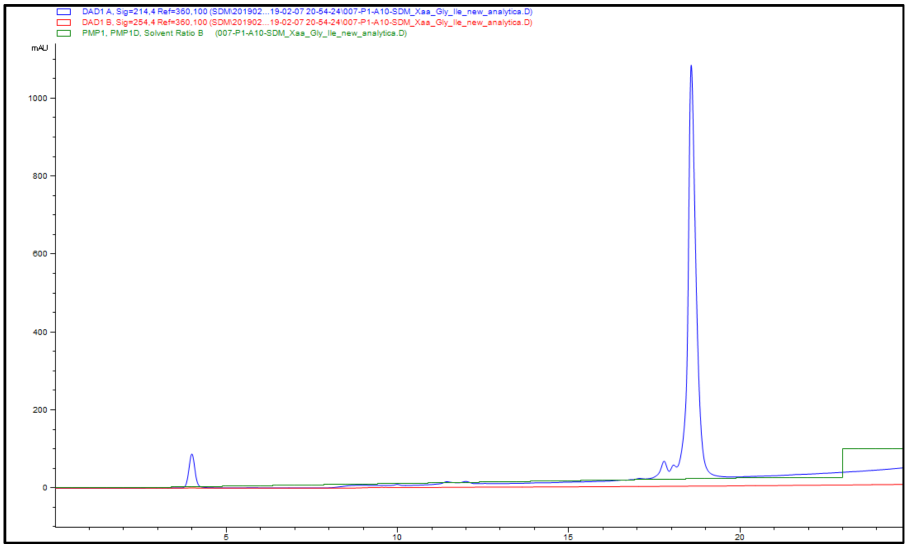

CMP **16b**: H-(Pro-Hyp-Gly)$_3$(Pro-Hyp-**azGly**)(**Ile**-Hyp-Gly)(Pro-Hyp-Gly)$_3$-NH$_2$

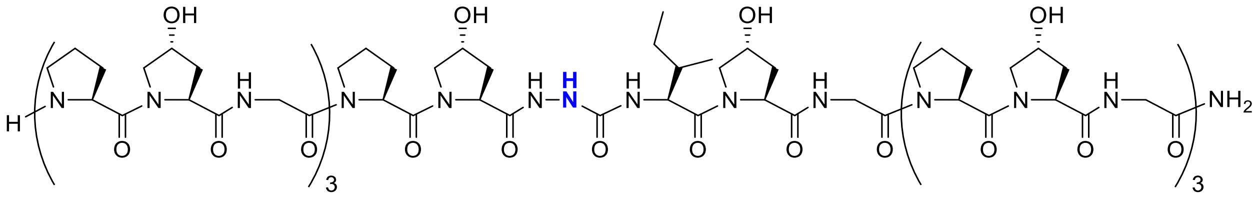

MALDI-TOF MS:

Calculated [M+Na]$^+$: 2195.021

Found: 2195.029

THERMAL DENATURATION CURVE:

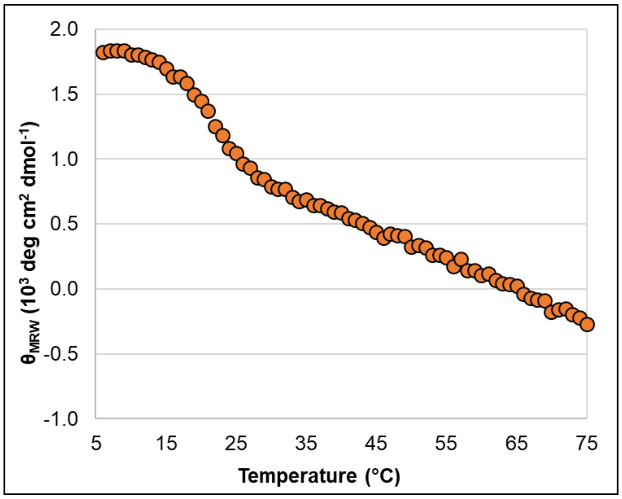

HPLC: 1-25% CH$_3$CN in 0.1% TFA H$_2$O over 30 min at 60 ˚C

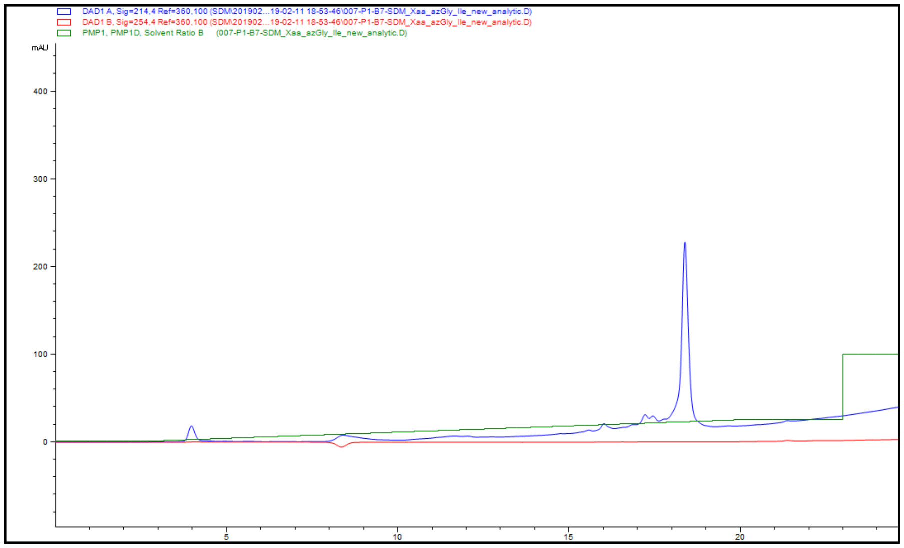

CMP **17a**: H-(Pro-Hyp-Gly)$_3$(Pro-Hyp-Gly)(**Met**-Hyp-Gly)(Pro-Hyp-Gly)$_3$-NH_2

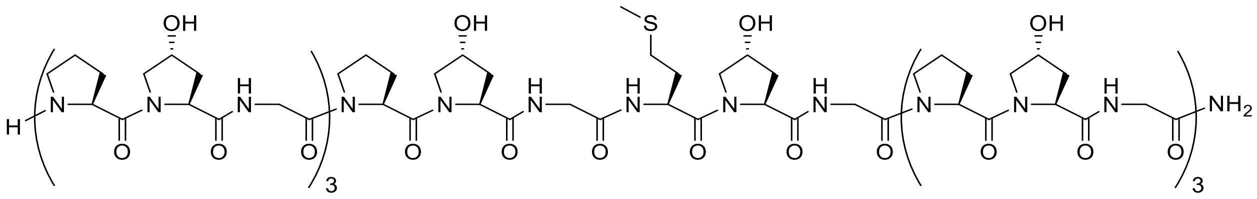

MALDI-TOF MS:

Calculated $[M+Na]^+$:	2211.983
Found:	2212.440

THERMAL DENATURATION CURVE:

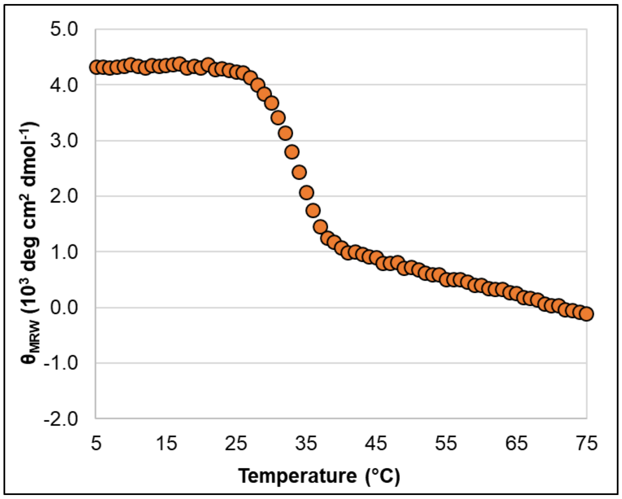

HPLC: 1-25% CH_3CN in 0.1% TFA H_2O over 30 min at 60 ˚C

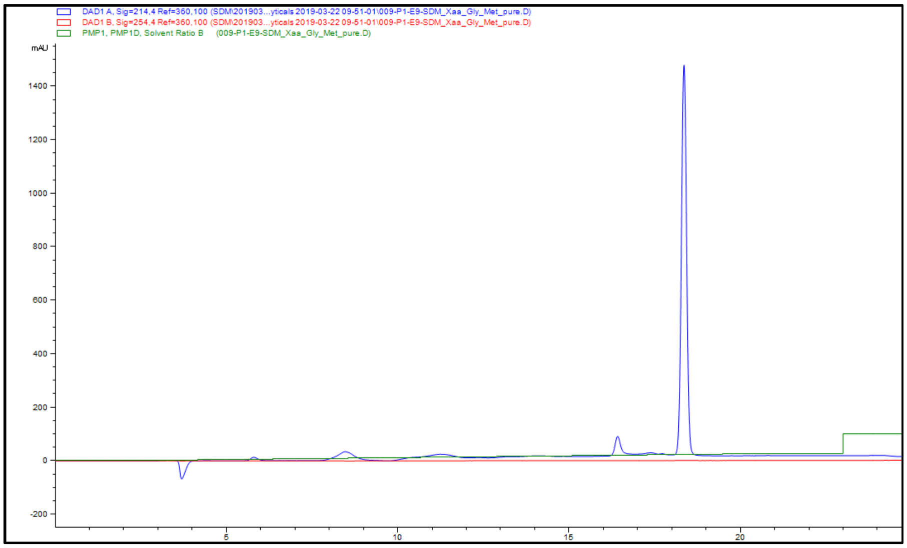

CMP **17b**: H-(Pro-Hyp-Gly)$_3$(Pro-Hyp-**azGly**)(**Met**-Hyp-Gly)(Pro-Hyp-Gly)$_3$-NH$_2$

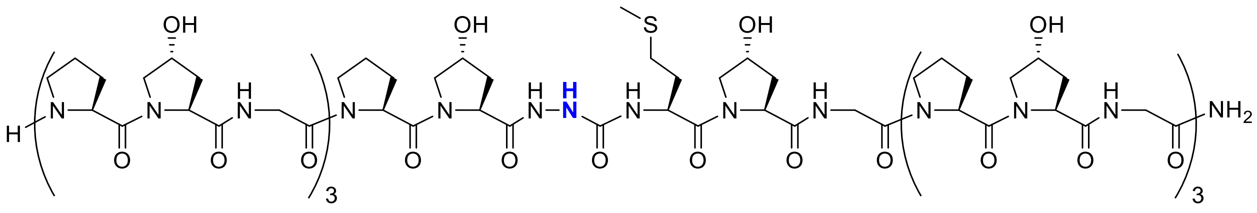

MALDI-TOF MS:

Calculated [M+Na]$^+$: 2212.978

Found: 2213.541

THERMAL DENATURATION CURVE:

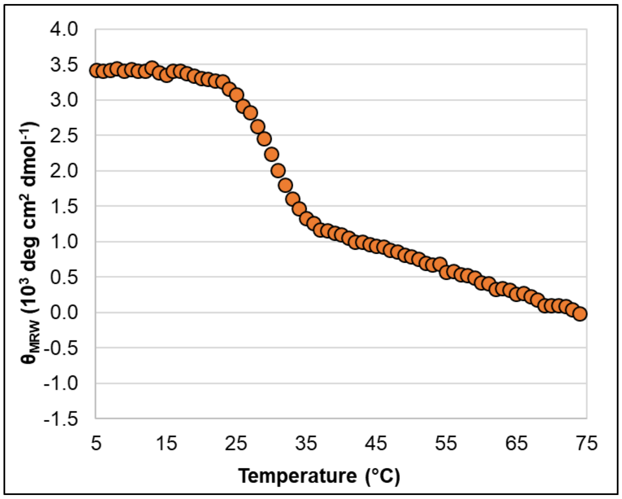

HPLC: 1-25% CH_3CN in 0.1% TFA H_2O over 30 min at 60 ˚C

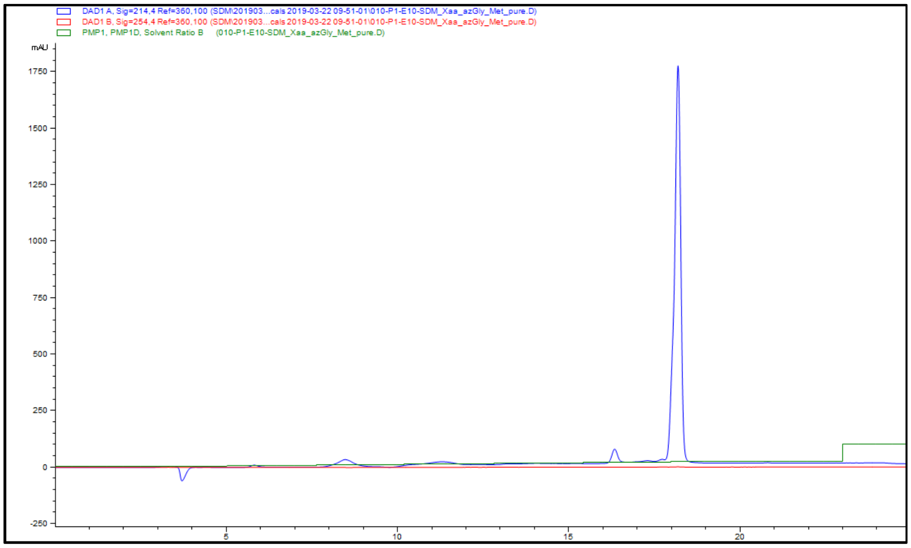

CMP **18a**: H-(Pro-Hyp-Gly)$_3$(Pro-Hyp-Gly)(**Phe**-Hyp-Gly)(Pro-Hyp-Gly)$_3$-NH_2

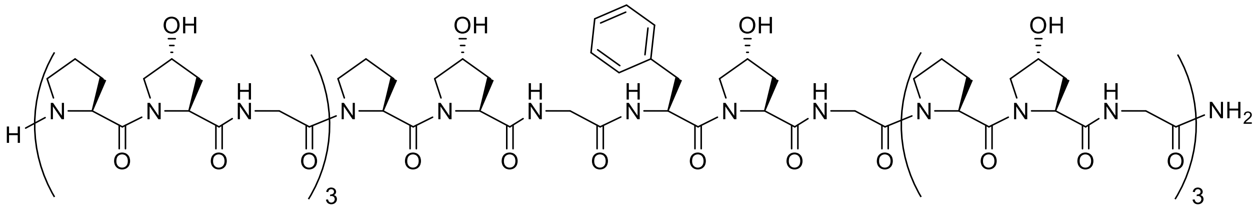

MALDI-TOF MS:

Calculated $[M+Na]^+$: 2228.011

Found: 2228.519

THERMAL DENATURATION CURVE:

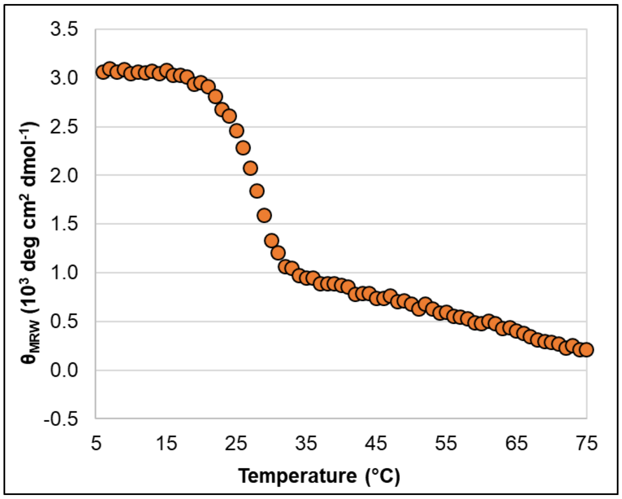

HPLC: 1-40% CH_3CN in 0.1% TFA H_2O over 30 min at 60 ˚C

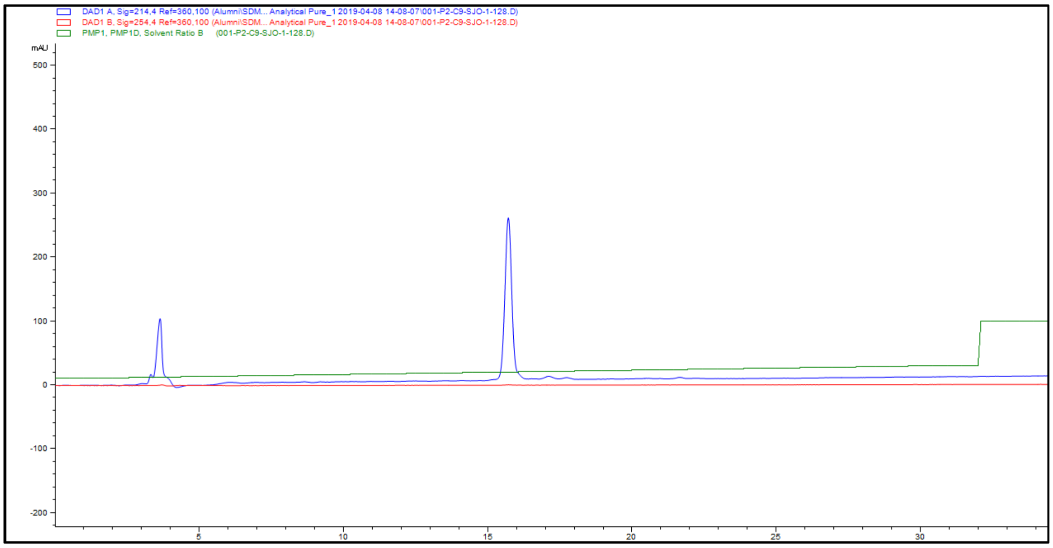

CMP **18b**: H-(Pro-Hyp-Gly)$_3$(Pro-Hyp-**azGly**)(**Phe**-Hyp-Gly)(Pro-Hyp-Gly)$_3$-NH$_2$

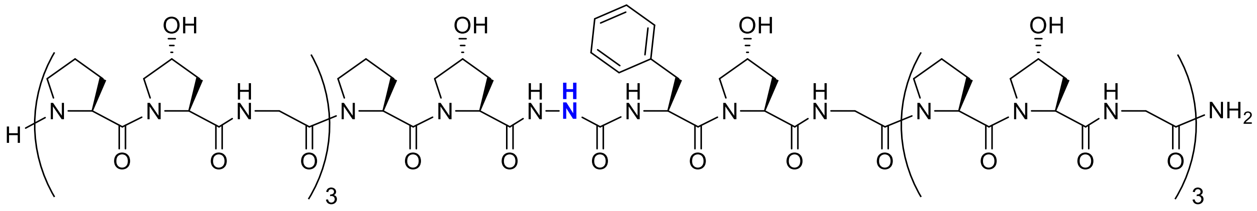

MALDI-TOF MS:

Calculated [M+H]$^+$: 2206.016

Found: 2206.406

THERMAL DENATURATION CURVE:

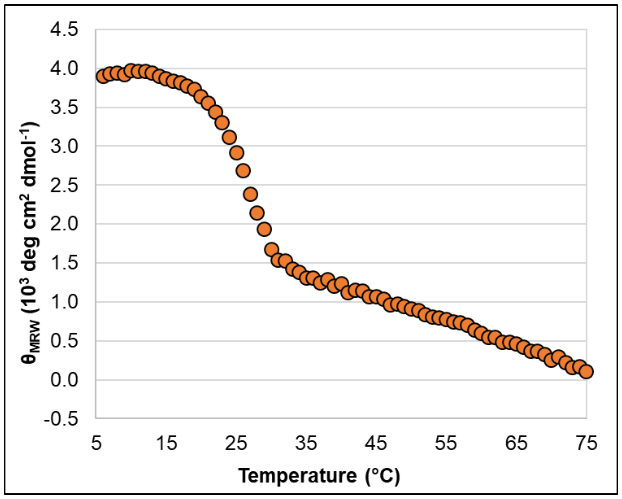

HPLC: 1-25% CH$_3$CN in 0.1% TFA H$_2$O over 30 min at 60 ˚C

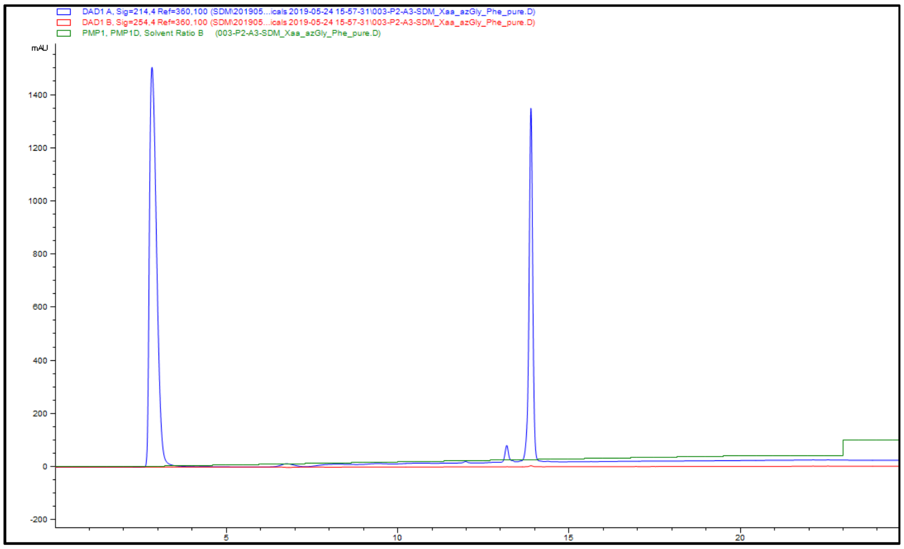

CMP **19a**: H-(Pro-Hyp-Gly)$_3$(Pro-Hyp-Gly)(**Tyr**-Hyp-Gly)(Pro-Hyp-Gly)$_3$-NH$_2$

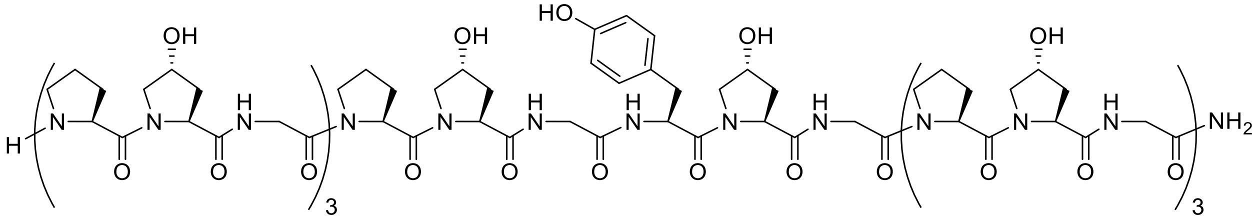

MALDI-TOF MS:

Calculated [M+Na]$^+$: 2244.006

Found: 2244.472

THERMAL DENATURATION CURVE:

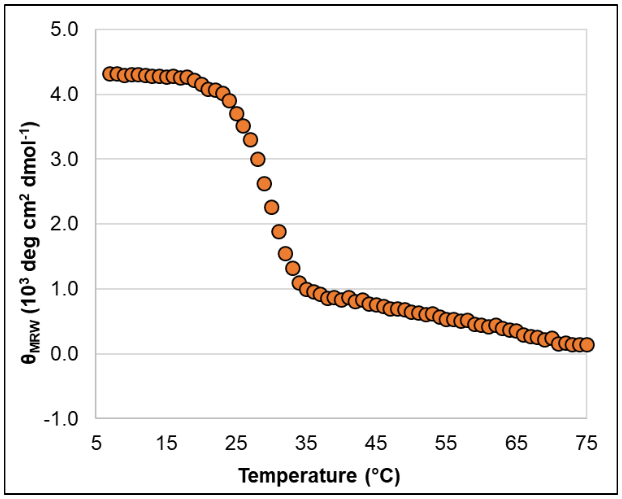

HPLC: 10-30% CH_3CN in 0.1% TFA H_2O over 30 min at 60 ˚C

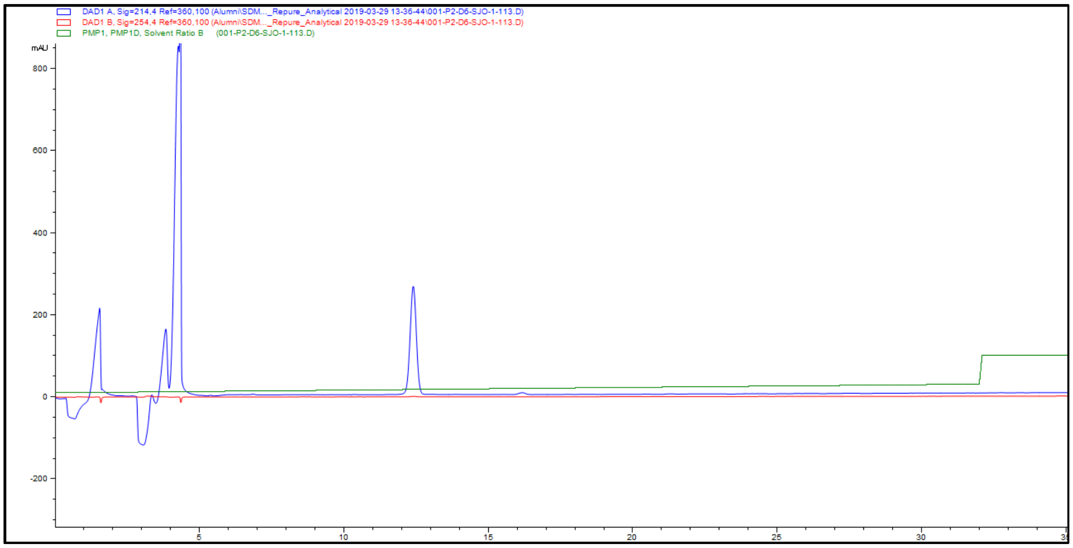

CMP **19b**: H-(Pro-Hyp-Gly)$_3$(Pro-Hyp-**azGly**)(**Tyr**-Hyp-Gly)(Pro-Hyp-Gly)$_3$-NH_2

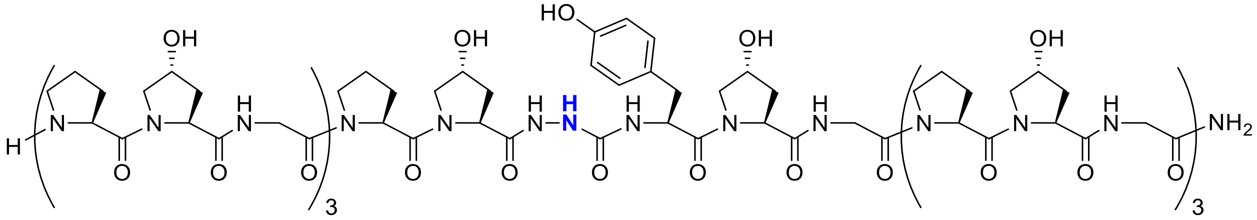

MALDI-TOF MS:

Calculated $[M+Na]^+$: 2245.001

Found: 2245.397

THERMAL DENATURATION CURVE:

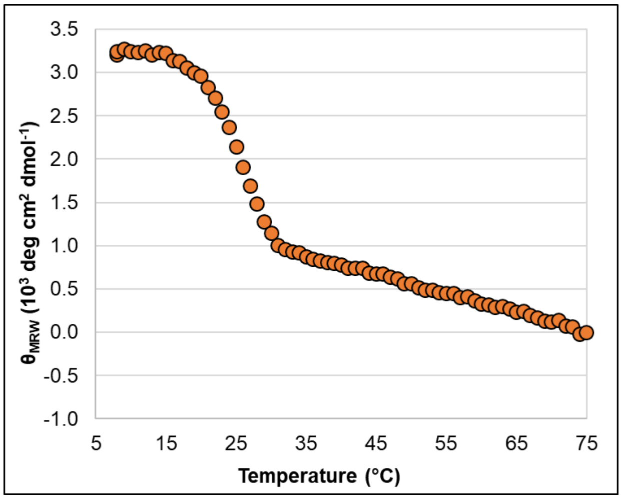

HPLC: 10-30% CH_3CN in 0.1% TFA H_2O over 30 min at 60 ˚C

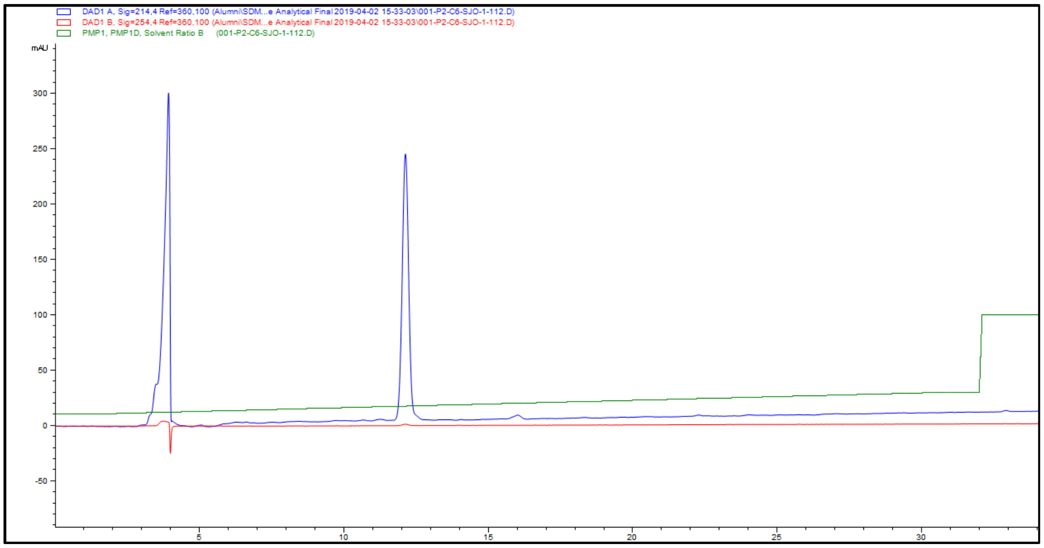

CMP **20a**: H-(Pro-Hyp-Gly)$_3$(Pro-Hyp-Gly)(**Trp**-Hyp-Gly)(Pro-Hyp-Gly)$_3$-NH$_2$

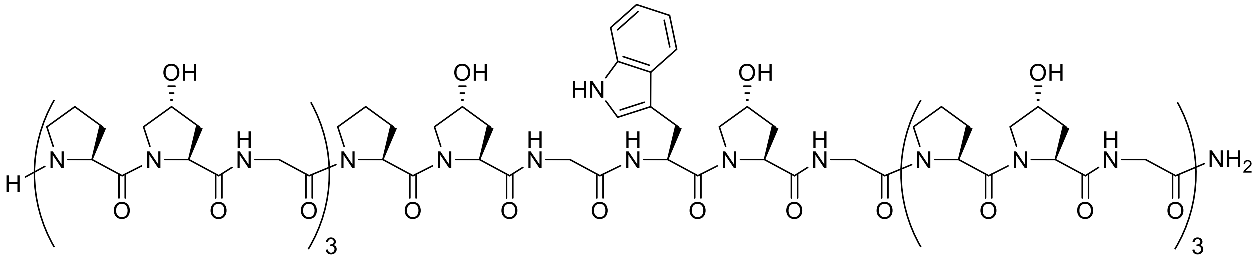

MALDI-TOF MS:

Calculated [M+Na]$^+$: 2267.022

Found: 2267.650

THERMAL DENATURATION CURVE:

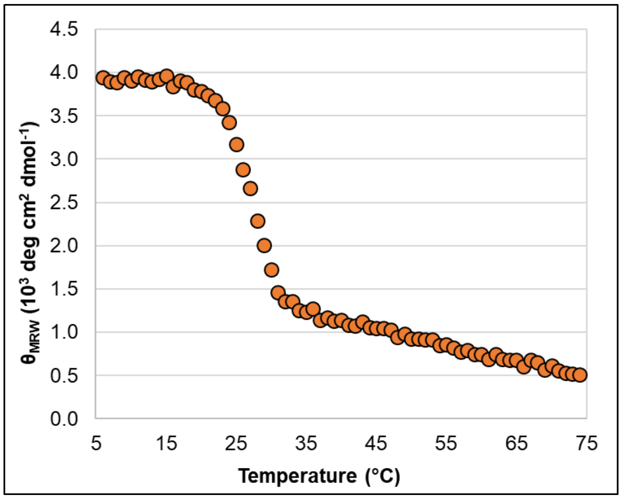

HPLC: 10-30% CH_3CN in 0.1% TFA H_2O over 30 min at 60 ˚C

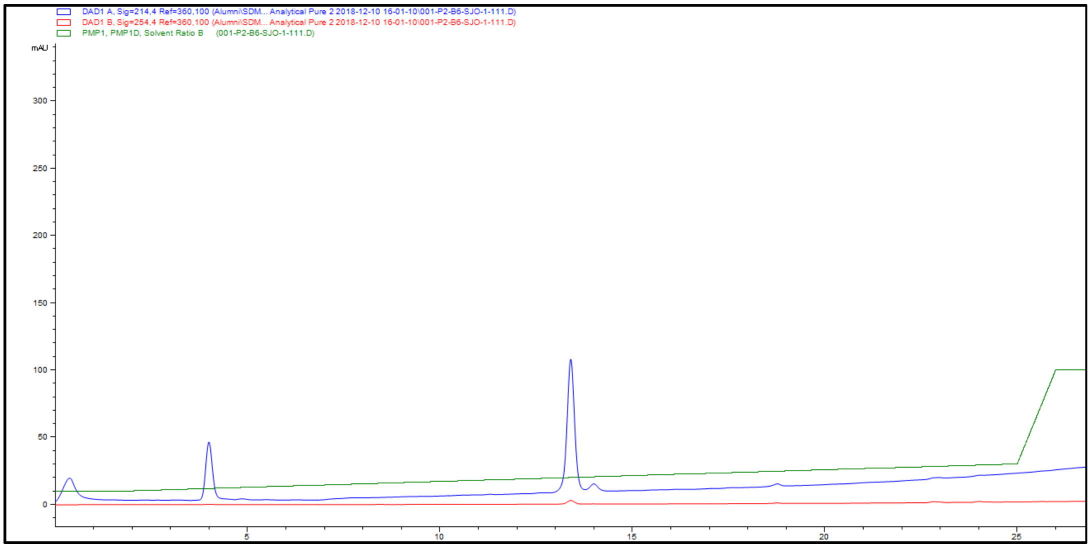

CMP **20b**: H-(Pro-Hyp-Gly)$_3$(Pro-Hyp-**azGly**)(**Trp**-Hyp-Gly)(Pro-Hyp-Gly)$_3$-NH$_2$

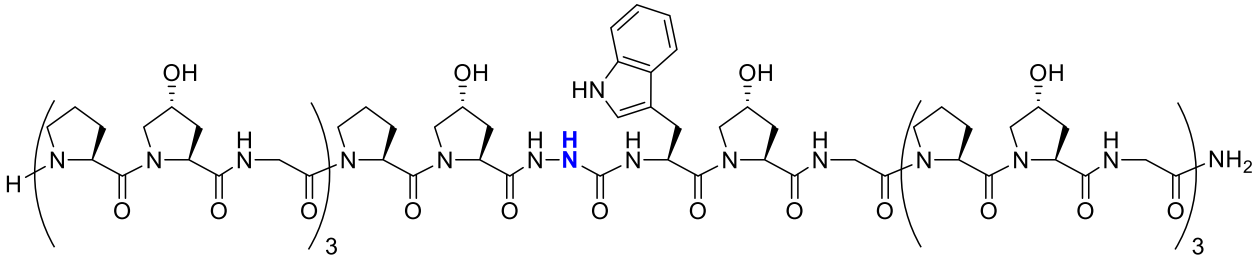

MALDI-TOF MS:

Calculated [M+Na]$^+$: 2268.017

Found: 2268.590

THERMAL DENATURATION CURVE:

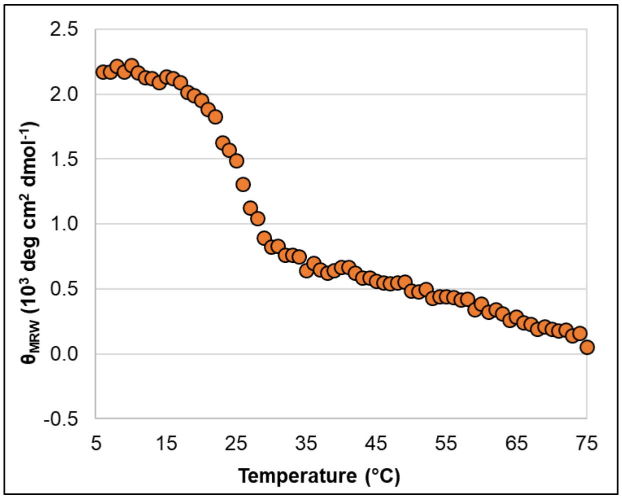

HPLC: 10-30% CH_3CN in 0.1% TFA H_2O over 30 min at 60 ˚C

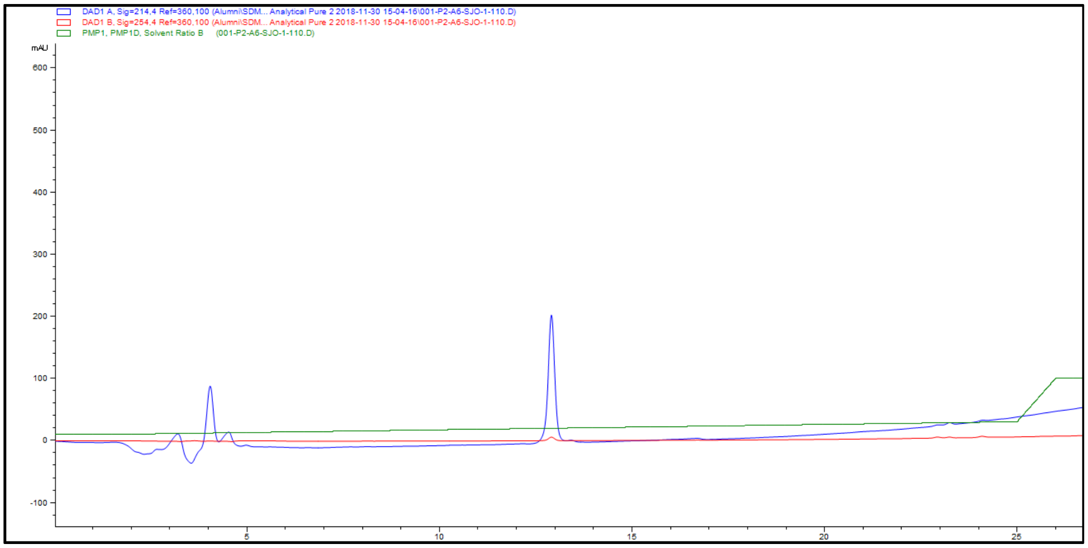

CMP **20c**: H-(Pro-Hyp-Gly)(Pro-Hyp-**azGly**)(Pro-Hyp-Gly)$_2$(**Trp**-Hyp-Gly)(Pro-Hyp-Gly)(Pro-Hyp-**azGly**)(Pro-Hyp-Gly)-NH_2

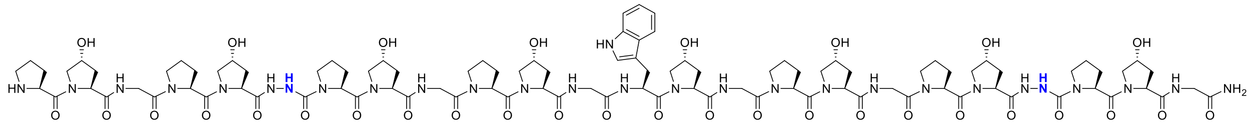

MALDI-TOF MS:

Calculated [M+Na]$^+$:	2269.012
Found:	2269.127

THERMAL DENATURATION CURVE:

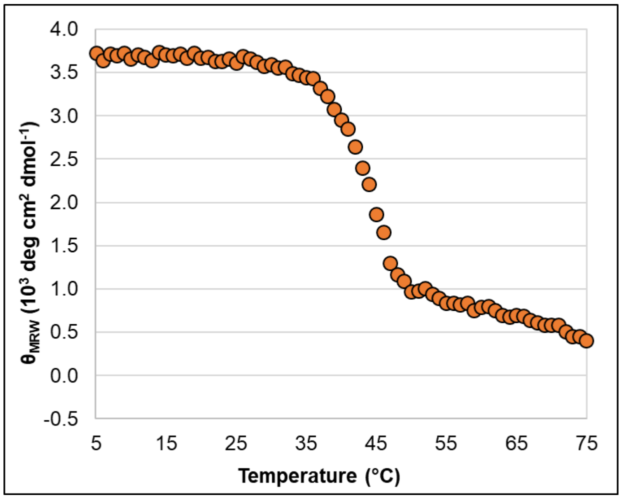

HPLC: 1-25% CH_3CN in 0.1% TFA H_2O over 30 min at 60 ˚C

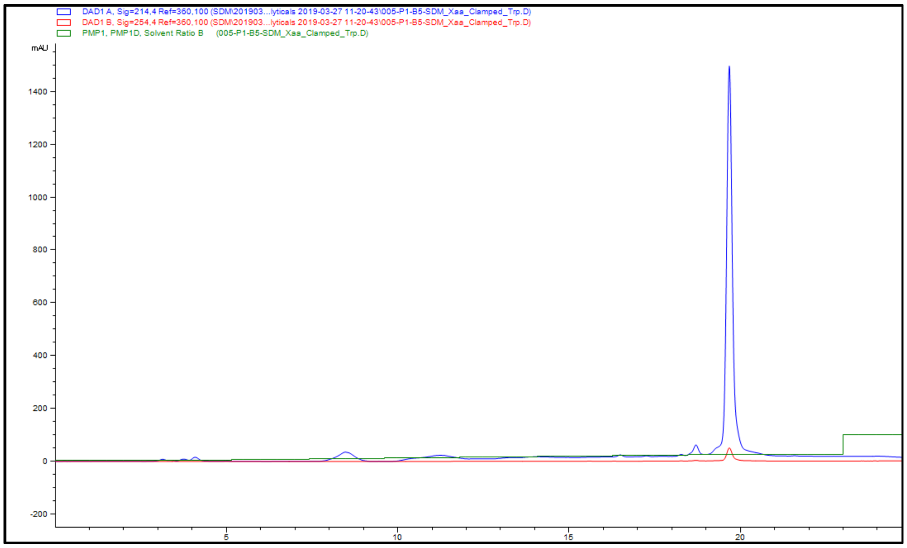

CMP **21a**: H-(Pro-Hyp-Gly)$_3$(Pro-Hyp-Gly)(**His**-Hyp-Gly)(Pro-Hyp-Gly)$_3$-NH_2

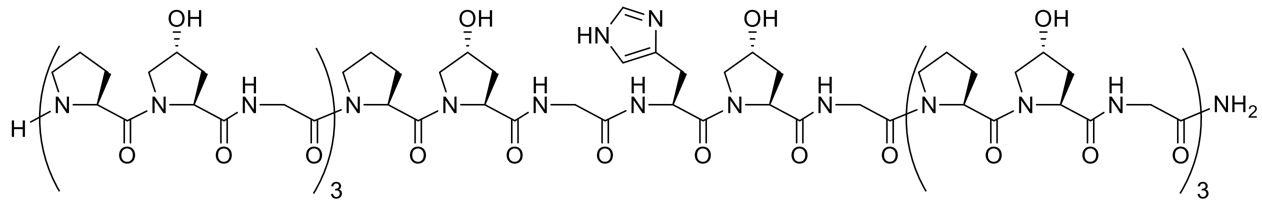

MALDI-TOF MS:

Calculated [M+Na]$^+$: 2218.001

Found: 2218.164

THERMAL DENATURATION CURVE:

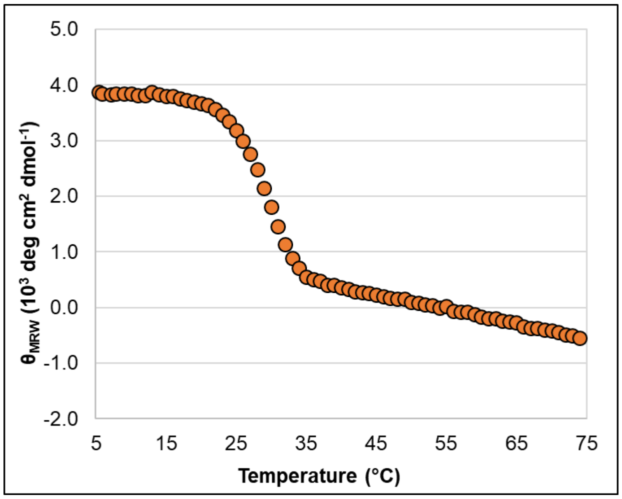

HPLC: 1-25% CH_3CN in 0.1% TFA H_2O over 30 min at 60 ˚C

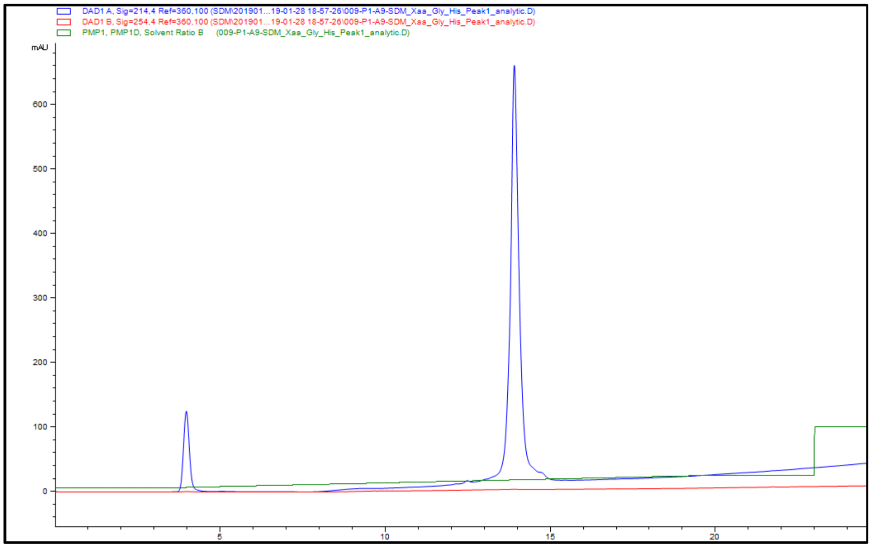

CMP **21b**: H-(Pro-Hyp-Gly)$_3$(Pro-Hyp-**azGly**)(**His**-Hyp-Gly)(Pro-Hyp-Gly)$_3$-NH$_2$

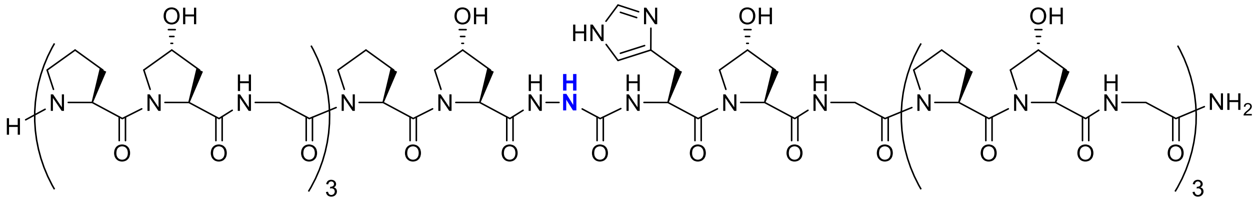

MALDI-TOF MS:

Calculated [M+Na]$^+$: 2218.996

Found: 2219.201

THERMAL DENATURATION CURVE:

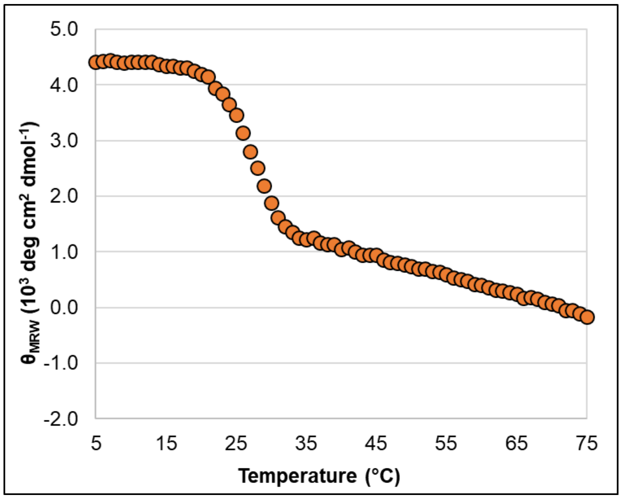

HPLC: 1-25% CH_3CN in 0.1% TFA H_2O over 30 min at 60 ˚C

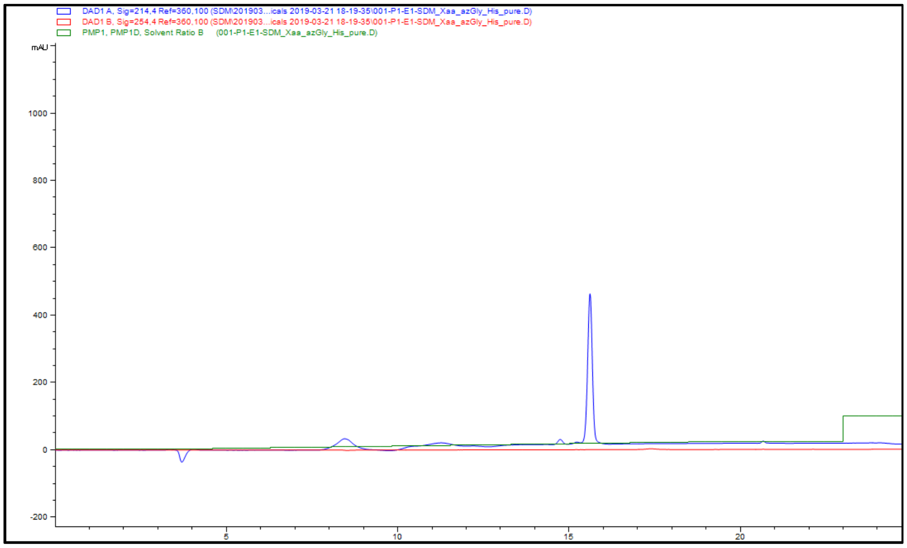

CMP **22a**: H-(Pro-Hyp-Gly)$_3$(Pro-Hyp-Gly)(**NmetG**-Hyp-Gly)(Pro-Hyp-Gly)$_3$-NH$_2$

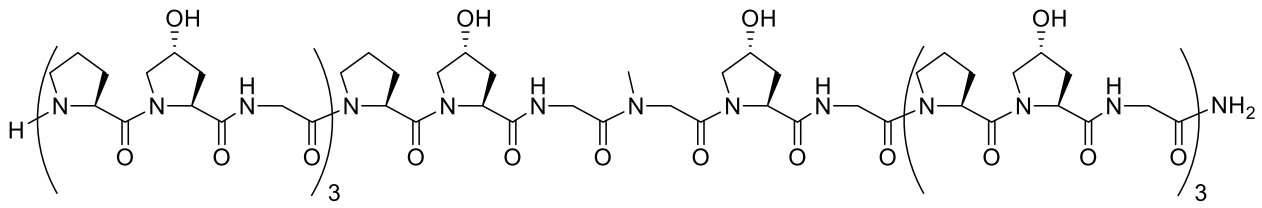

MALDI-TOF MS:

Calculated [M+Na]$^+$: 2151.980

Found: 2151.954

THERMAL DENATURATION CURVE:

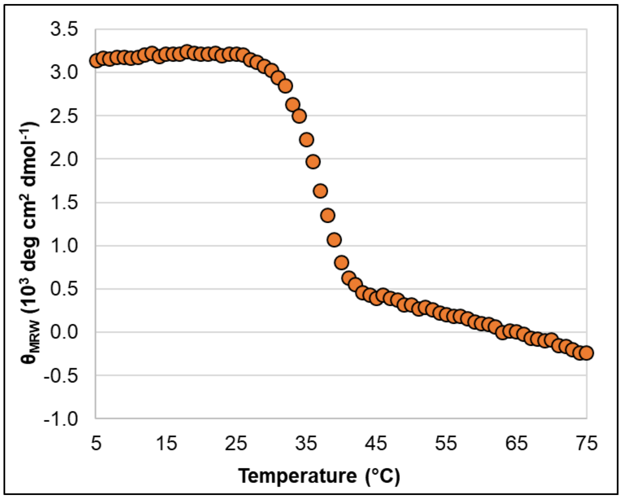

HPLC: 1-25% CH$_3$CN in 0.1% TFA H$_2$O over 30 min at 60 ˚C

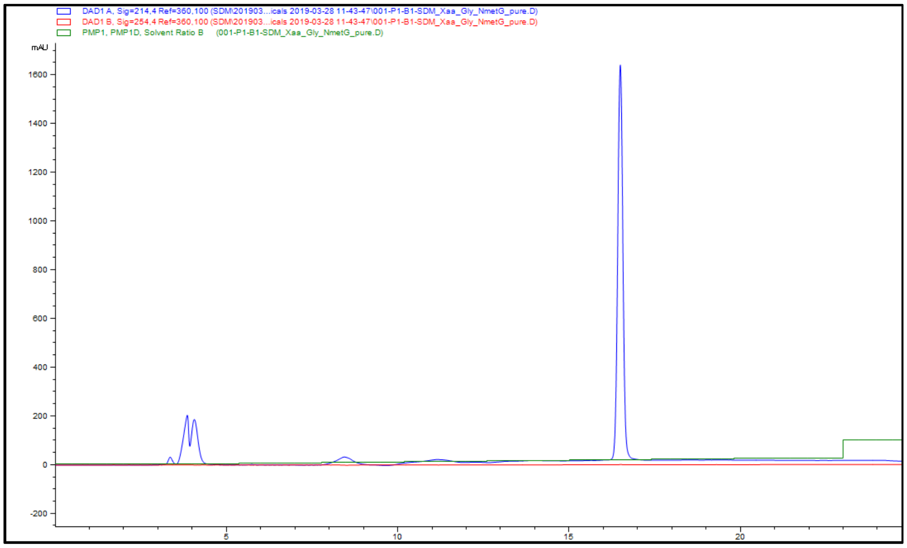

CMP **22b**: H-(Pro-Hyp-Gly)$_3$(Pro-Hyp-**azGly**)(**NmetG**-Hyp-Gly)(Pro-Hyp-Gly)$_3$-NH$_2$

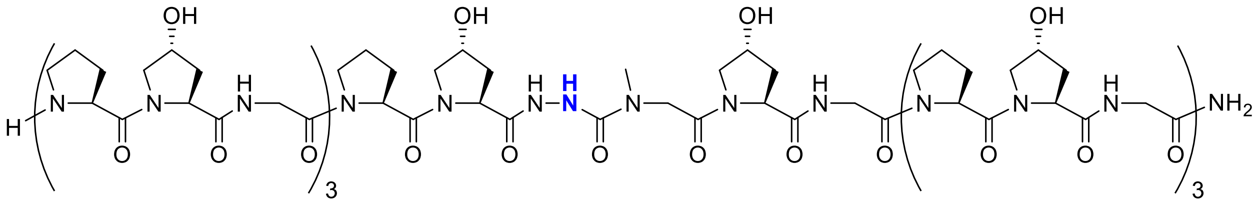

MALDI-TOF MS:

Calculated [M+Na]$^+$:	2152.975
Found:	2153.509

THERMAL DENATURATION CURVE:

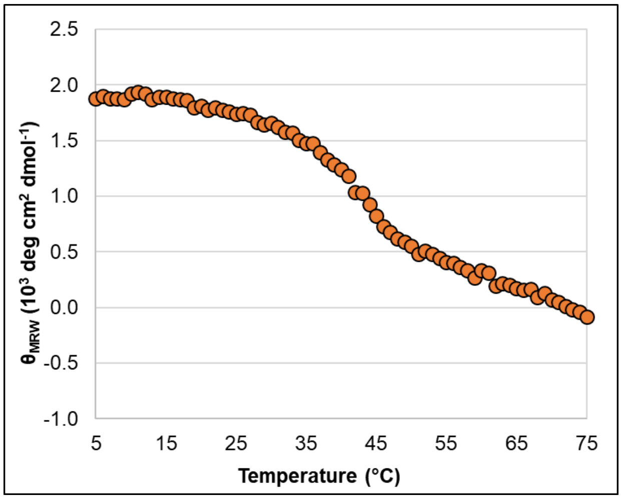

HPLC: 1-25% CH$_3$CN in 0.1% TFA H$_2$O over 30 min at 60 ˚C

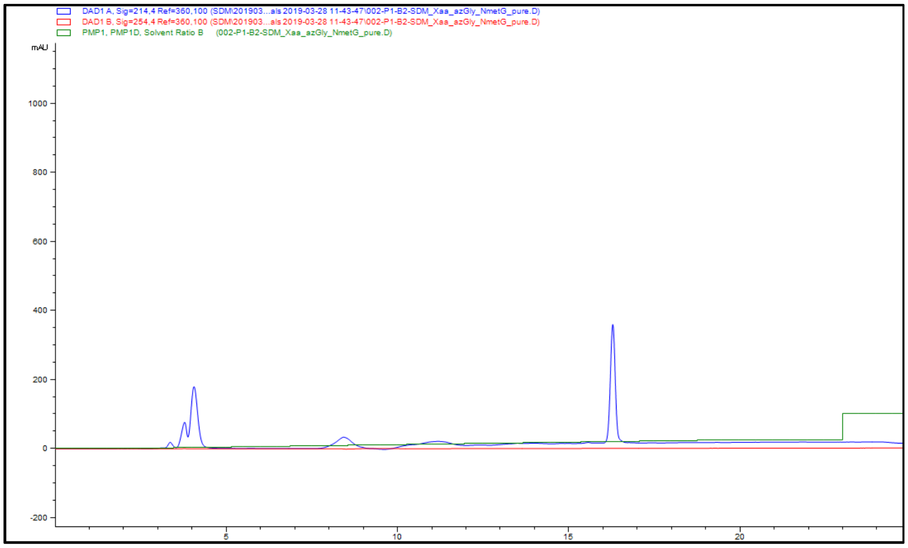

CMP **23a**: H-(Pro-Hyp-Gly)$_3$(Pro-Hyp-Gly)(**NethylG**-Hyp-Gly)(Pro-Hyp-Gly)$_3$-NH$_2$

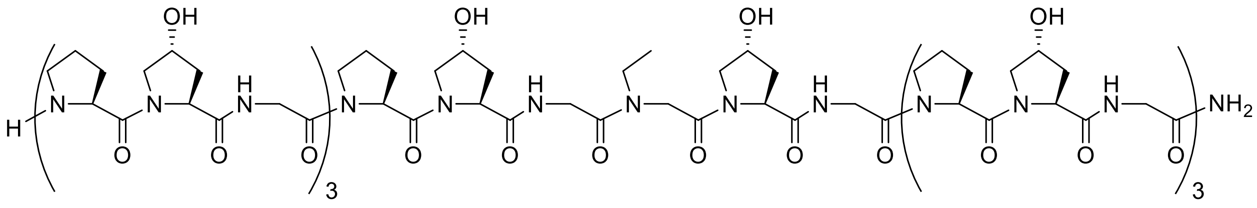

MALDI-TOF MS:

Calculated [M+Na]$^+$: 2165.995

Found: 2165.470

THERMAL DENATURATION CURVE:

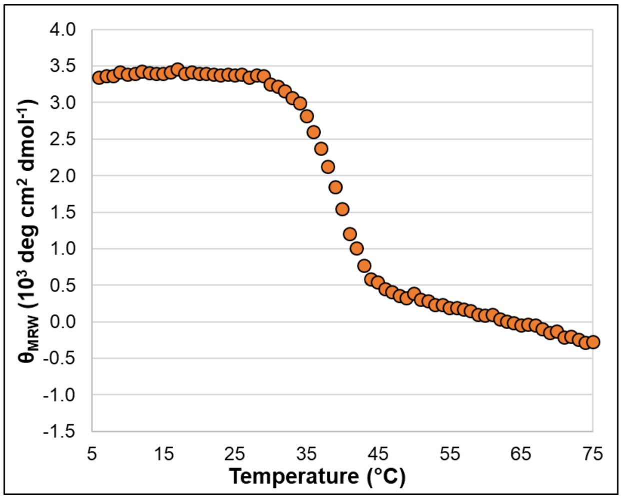

HPLC: 5-35% CH_3CN in 0.1% TFA H_2O over 30 min at 80 ˚C

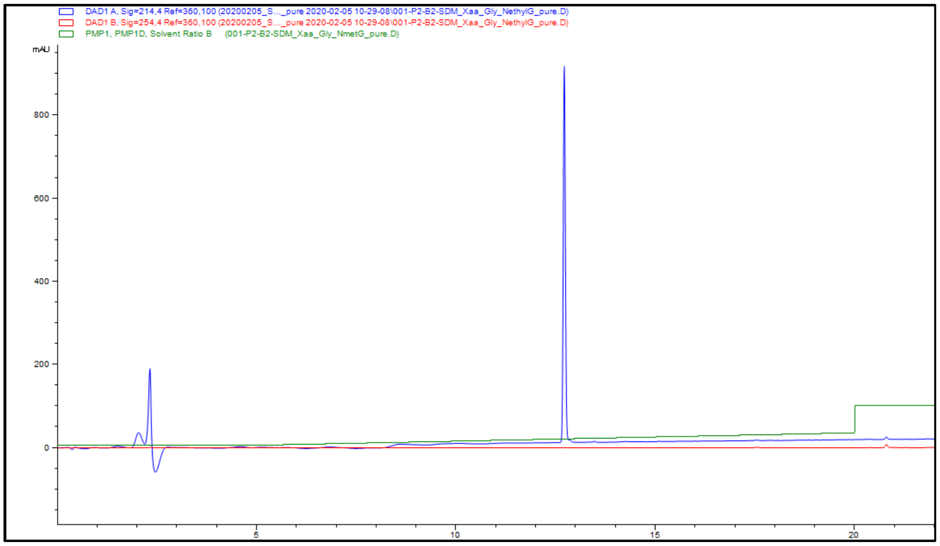

CMP **23b**: H-(Pro-Hyp-Gly)$_3$(Pro-Hyp-**azGly**)(**NethylG**-Hyp-Gly)(Pro-Hyp-Gly)$_3$-NH$_2$

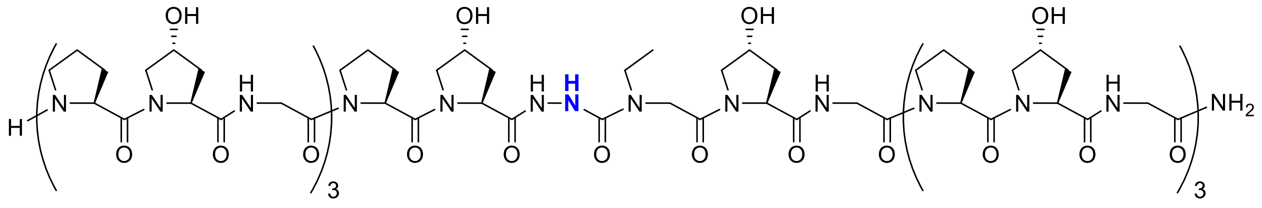

MALDI-TOF MS:

Calculated [M+Na]$^+$:	2166.990
Found:	2167.335

THERMAL DENATURATION CURVE:

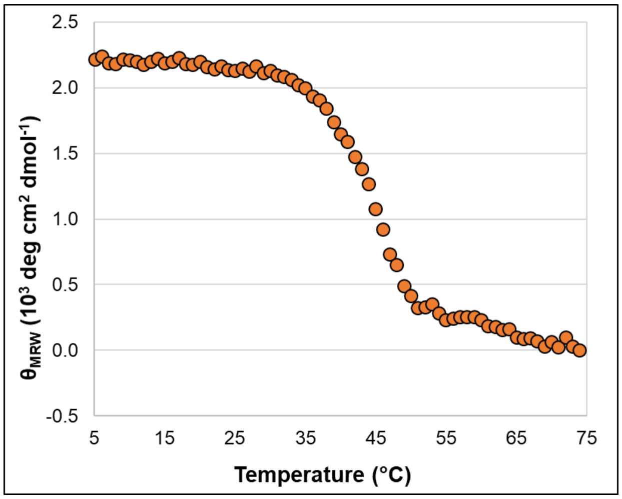

HPLC: 1-25% CH$_3$CN in 0.1% TFA H$_2$O over 30 min at 60 ˚C

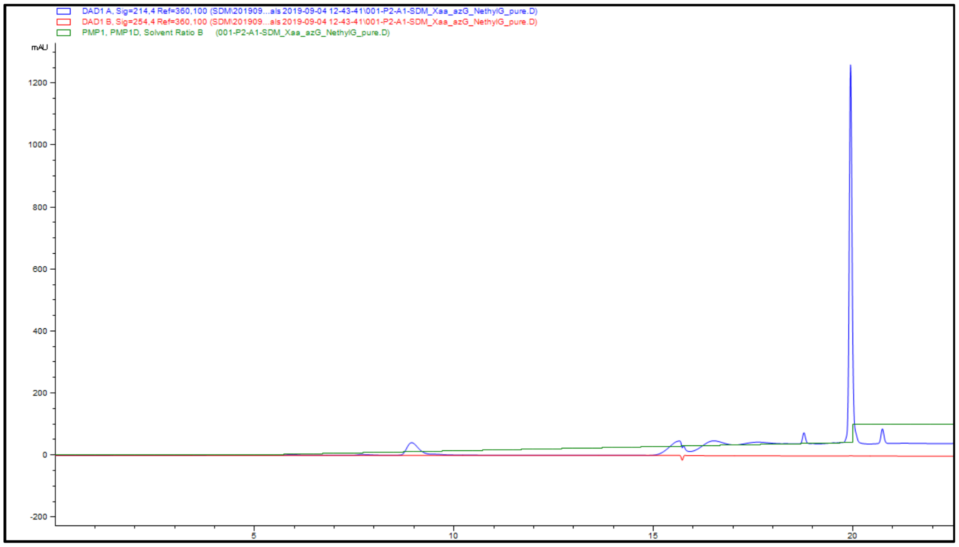

CMP **24**: H-(Pro-Hyp-Gly)$_2$(Pro-Arg-Gly)(Pro-Hyp-Gly)$_2$(**Val**-Hyp-Gly)(Pro-Hyp-Gly)$_2$-(BrF-Hyp-Gly)-(Pro-Hyp-Gly)-NH_2

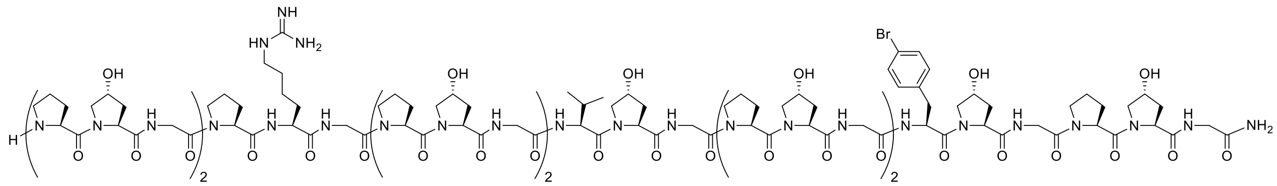

MALDI-TOF MS:

Calculated $[M+Na]^+$: 2887.232

Found: 2887.088

Representative Crystal:

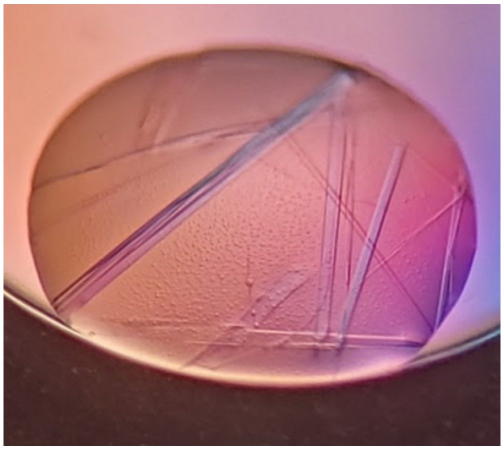

HPLC: 5-35% CH_3CN in 0.1% TFA H_2O over 30 min at 80 ˚C

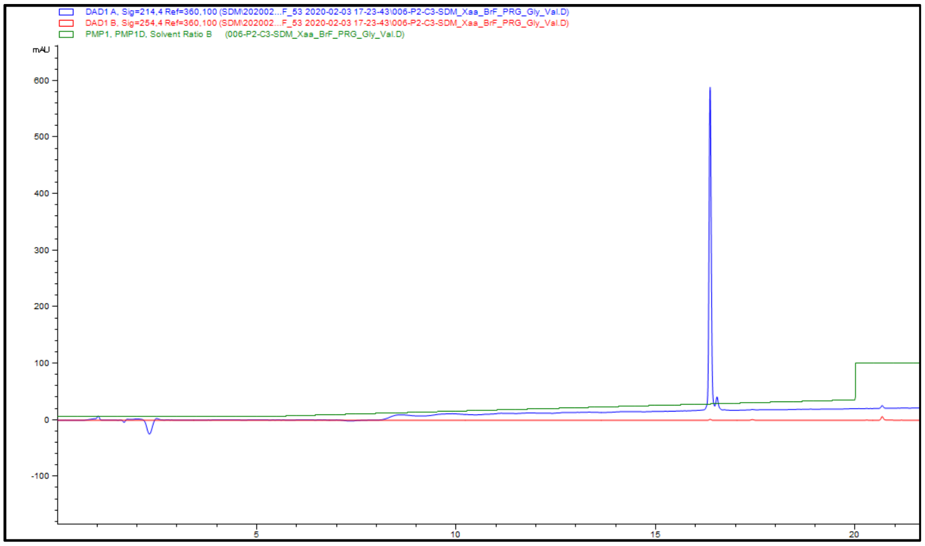

CMP **25**: H-(Pro-Hyp-Gly)$_2$(Pro-Arg-Gly)(Pro-Hyp-Gly)$_3$(**NmetG**-Hyp-Gly)(Pro-Hyp-Gly)$_2$-NH$_2$

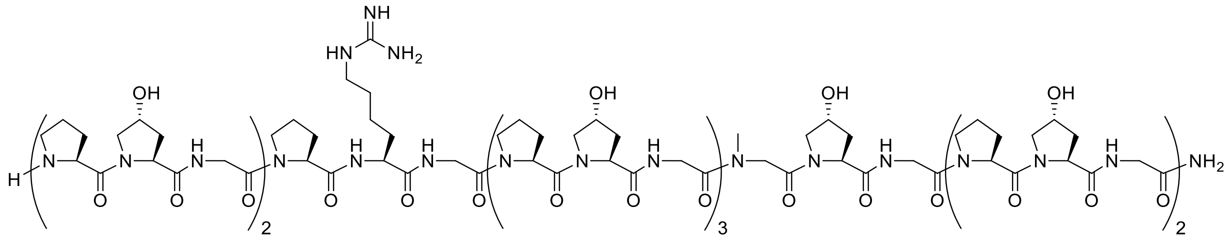

MALDI-TOF MS:

Calculated [M+Na]$^+$: 2462.155

Found: 2462.236

Representative Crystal:

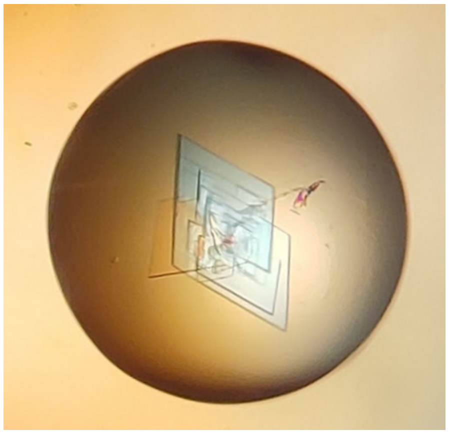

HPLC: 5-35% CH_3CN in 0.1% TFA H_2O over 30 min at 80 ˚C

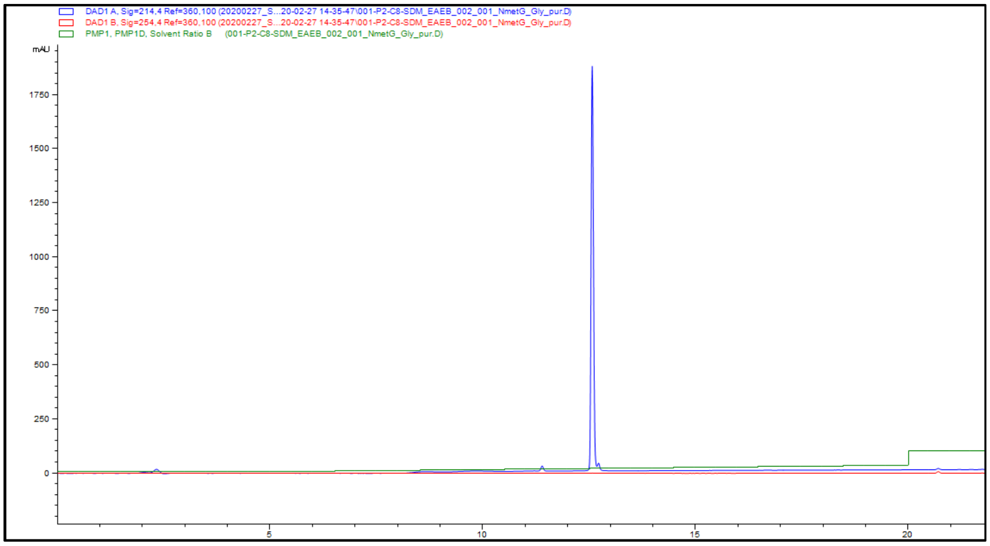

REFERENCES

1. Zhang, Y., Malamakal, R. M. & Chenoweth, D. M. Aza-Glycine Induces Collagen Hyperstability. *J. Am. Chem. Soc.* **137**, 12422–12425 (2015).
2. Zhang, Y., Herling, M. & Chenoweth, D. M. General Solution for Stabilizing Triple Helical Collagen. *J. Am. Chem. Soc.* **138**, 9751–9754 (2016).
3. Engel, J., Chen, H.-T., Prockop, D. J. & Klump, H. The triple helix coil conversion of collagen-like polytripeptides in aqueous and nonaqueous solvents. Comparison of the thermodynamic parameters and the binding of water to (L-Pro-L-Pro-Gly) n and (L-Pro-L-Hyp-Gly) n. *Biopolymers* **16**, 601–622 (1977).
4. Okuyama, K., Haga, M., Noguchi, K. & Tanaka, T. Preferred side-chain conformation of arginine residues in a triple-helical structure: Preferred Side-Chain Conformation of Arg in Collagen Helix. *Biopolymers* **101**, 1000–1009 (2014).
5. Kabsch, W. XDS. *Acta Crystallogr. Sect. D* **66**, 125–132 (2010).
6. Battye, T. G. G., Kontogiannis, L., Johnson, O., Powell, H. R. & Leslie, A. G. W. iMOSFLM: a new graphical interface for diffraction-image processing with ıt MOSFLM. *Acta Crystallogr. Sect. D* **67**, 271–281 (2011).
7. Evans, P. R. & Murshudov, G. N. How good are my data and what is the resolution? *Acta Crystallogr. D Biol. Crystallogr.* **69**, 1204–1214 (2013).
8. Winn, M. D. *et al.* Overview of the ıt CCP4 suite and current developments. *Acta Crystallogr. Sect. D* **67**, 235–242 (2011).
9. McCoy, A. J. *et al.* Phaser crystallographic software. *J. Appl. Crystallogr.* **40**, 658–674 (2007).
10. Moriarty, N. W., Grosse-Kunstleve, R. W. & Adams, P. D. electronic Ligand Builder and Optimization Workbench (ıt eLBOW): a tool for ligand coordinate and restraint generation. *Acta Crystallogr. Sect. D* **65**, 1074–1080 (2009).
11. Adams, P. D. *et al.* PHENIX: a comprehensive Python-based system for macromolecular structure solution. *Acta Crystallogr. Sect. D* **66**, 213–221 (2010).
12. Emsley, P. & Cowtan, K. Coot: model-building tools for molecular graphics. *Acta Crystallogr. Sect. D* **60**, 2126–2132 (2004).
13. Pettersen, E. F. *et al.* UCSF Chimera - A visualization system for exploratory research and analysis. *J. Comput. Chem.* **25**, 1605–1612 (2004).
